# Influence of integrated services on postpartum family planning use: a cross-sectional survey from urban Senegal

**DOI:** 10.1186/1471-2458-13-752

**Published:** 2013-08-14

**Authors:** Ilene S Speizer, Jean Christophe Fotso, Chinelo Okigbo, Cheikh Mbacké Faye, Cheikh Seck

**Affiliations:** 1 Department of Maternal and Child Health, Gillings School of Global Public Health, University of North Carolina, Chapel Hill, NC, USA; 2 Measurement, Learning, and Evaluation Project, Carolina Population Center, University of North Carolina, Chapel Hill, NC, USA; 3 Concern Worldwide, New York, NY, USA; 4 African Population Health and Research Center, Nairobi, Kenya; 5 Initiative Sénégalaise de Santé Urbaine (ISSU), IntraHealth International, Dakar, Senegal

**Keywords:** Postpartum family planning, Senegal, Integrated services, Urban, Maternal, Newborn, and Child health

## Abstract

**Background:**

Although the majority of postpartum women indicate a desire to delay a next birth, family planning (FP) methods are often not offered to, or taken up by, women in the first year postpartum. This study uses data from urban Senegal to examine exposure to FP information and services at the time of delivery and at child immunization appointments and to determine if these points of integration are associated with greater use of postpartum FP.

**Methods:**

A representative, household sample of women, ages 15–49, was surveyed from six cities in Senegal in 2011. This study focuses on women who were within two years postpartum (n = 1879). We also include women who were surveyed through exit interviews after a visit to a high volume health facility in the same six cities; clients included were visiting the health facility for delivery, post-abortion care, postnatal care, and child immunization services (n = 794). Descriptive analyses are presented to examine exposure to FP services among postpartum women and women visiting the health facility. Logistic regression models are used to estimate the effect of integrated services on postpartum FP use in the household sample of women. Analyses were conducted using Stata version 12.

**Results:**

Among exit interview clients, knowledge of integrated services is high but only a few reported receiving FP services. A majority of the women who did not receive FP services indicated an interest in receiving such information and services.

Among the household sample of women up to two-years postpartum, those who received FP information at the time of delivery are more likely to be using modern FP postpartum than their counterparts who also delivered in a facility but did not receive such information. Exposure to FP services at an immunization visit was not significantly related to postpartum FP use. Another key finding is that women with greater self-efficacy are more likely to use a modern FP method.

**Conclusion:**

This study’s findings lend strong support for the need to improve integration of FP services into maternal, newborn, and child health services with the goal of increasing postpartum women’s use of FP methods in urban Senegal.

## Background

The high rate of unintended pregnancy is said to be driving population growth, especially in sub-Saharan Africa where approximately one-half of all pregnancies are reported to be unintended, that is they came too soon or were unwanted [1]. These pregnancies can be prevented with increased access to effective family planning (FP) methods. Research and interventions focus on improving access to FP services especially in the developing world where it is estimated that 222 million women are sexually active, want to delay or avoid a birth, and are not using modern FP (i.e., they have an unmet need for FP) [2]. Women in their first year after childbirth have the highest unmet need for FP as more than two-thirds of these women want to delay their next birth but are not using any method [3,4]. The postpartum period is an important intervention point for improving access to family planning services. This period is critical for two reasons: a) postpartum women have a high need for FP, and b) these women have multiple contacts with the health facility either for postnatal or child immunization visits.

Although the majority of postpartum women indicate a desire to delay a next birth, FP methods are often not offered to, or taken up by, women after delivery or in the first year postpartum [3,5-7]. Based on the Health Belief Model, which has been widely used in the study of contraceptive use, perceived threat (perceived susceptibility and perceived severity), perceived benefits, cues to action, and self-efficacy are known to influence contraceptive behaviors [8-10]. Women must perceive their susceptibility to conception, the consequences of an unintended pregnancy, and the benefits of preventing such pregnancy to trigger their use of FP. The Health Belief Model was developed by Rosenstock in the early 1970s and is a cognitive interpersonal theoretical framework used in understanding the complex multidimensionality of contraceptive use decision-making [10,11]. Earlier studies from sub-Saharan Africa demonstrate that the majority of women lack adequate knowledge of the return of fertility after birth with most of the women, as well as their providers, using resumption of menses as the measure of susceptibility or cue for FP use [4,5,7,12]; these women are at risk of an unwanted or mistimed pregnancy. Seizing the opportunity to offer FP services to women in the postpartum period may result in a steeper increase in the uptake of FP methods and a reduction in unintended pregnancies.

The promotion of FP to delay conception after a recent birth is a best practice that can lead to optimal maternal and child health outcomes. In particular, short inter-pregnancy intervals can result in negative health outcomes such as maternal anemia, low birth weight, and neonatal/infant mortality [13-15]. In addition, short birth intervals are correlated with less breastfeeding of the child prior to the subsequent pregnancy; this may have implications on child health and mother-child bonding [16]. Therefore, the promotion and use of FP for at least two years postpartum will prevent unintended pregnancies and ensure adequate birth intervals.

One way to increase access to FP services is through its integration with maternal and child health (MCH) services. Family planning can be integrated into MCH services at various intervention points. These points include: antenatal care, delivery, postnatal visits, and child immunization visits. To ensure that all postpartum women are reached, no matter their place of delivery, there is a need to integrate FP services into all of these points. Choosing to integrate at one point over the other may result in some women not being reached. For example, integrating only at delivery will miss women who did not deliver at the health facility. That said, comparative research is needed as to where it will be most effective to integrate these services especially for resource-constrained countries. Our study hopes to begin to fill this gap.

This study aims to assess the role of integrated MCH/FP services on use of postpartum family planning by: 1) examining women’s exposure to FP information and services at the time of delivery and at child immunization appointments, and 2) determining if these points of integration are associated with greater use of postpartum FP. Using the Health Belief Model as a theoretical framework [8-11], we assess the factors that influence postpartum FP use in urban cities in Senegal. We examine the association between exposure to FP information and services at the time of delivery and at a child immunization visit with current modern FP use among women who are up to two years postpartum. These MCH points of integration are important as they capture the overwhelming majority of women in urban Senegal where in 2010/2011, 93% of urban births in the five years preceding the survey were delivered in a health facility and 63% of 12–23 month olds were fully vaccinated [17]. Senegal is an important setting for this type of study given that modern contraceptive method use is low overall (12% of married women report modern method use) and in urban areas (20%) [17].

Our focus on urban areas is due to the high rate of urbanization in sub-Saharan Africa which is said to result in concentrated poverty [18,19]. This poverty has created an intra-urban disparity in maternal health care as urban poor women have limited access to maternal health services compared to their wealthier counterparts [18]. The United Nations Population Fund (UNFPA) identifies reduction in rates of unwanted pregnancy as the most effective way to slow the rate of urban growth in the developing world [19]. Assessing contraceptive use among urban postpartum women and investigating the extent of association with exposure to information and advice before, during and/or after delivery and during child immunization services will provide much needed information that will inform programs and policies targeted at increasing access to postpartum FP services and improved health outcomes for mothers and babies. We use recently collected household surveys and exit interviews with women of reproductive age in urban Senegal to assess the effect of integrated services on the prevalence of postpartum FP use.

## Methods

This study uses recently collected data from the Measurement, Learning & Evaluation (MLE) project. In 2011, baseline data were collected for the evaluation of the Initiative Sénégalaise de Santé Urbaine (ISSU) in Senegal. Data were collected from six urban sites: Dakar, Guédiawaye, Pikine, Mbao, Mbour, and Kaolack. In each site, a multi-stage sampling design was used to select a representative sample of women ages 15–49 to include in the study. In the first stage, 32–64 primary sampling units (PSU) were selected with probability proportional to population size of each site; larger sites had more PSUs while smaller sites had fewer PSUs. Prior to selection, PSUs were classified as poor or non-poor based on neighborhood characteristics of the surroundings where the PSUs are located. Half of the selected PSUs in each site were selected from PSUs classified as poor and the other half were selected from PSUs classified as non-poor. This stratification permitted oversampling of poor women; weights were adjusted so that the data were representative of the sites. From selected PSU, a random sample of 21 households was selected and all women aged 15–49 from selected households were eligible for interview. Each woman was asked to give written consent to participate in the study. Prior to the interviews, the household head agreed that the interviewer can approach eligible teenagers to request their participation in the study; this is the standard approach used to survey persons under age 18 in these types of household surveys in Senegal. A total of 9614 women were surveyed from the six urban sites; the response rate was 88.9% [20]. For the analysis of postpartum family planning use, we focus only on women currently in union (i.e. married or living with a partner) who had a birth in the last two years and were not pregnant at the time of the survey (n = 2020). When sample weights are applied to the analysis sample to adjust for the sample size at the different sites and non-response, the weighted analysis sample size is 1879. Additional file 1 contains the questionnaire used for the women’s interviews in the six study sites.

We also collected data from health facilities in the six sites; this included high volume public and private health facilities as well as lower volume public and private health centers. A total of 205 health facilities were included in the study. In high volume public and private facilities (n = 55), we undertook exit interviews with family planning and reproductive health service clients. A total of 2686 clients seeking family planning and reproductive health services were approached in high volume facilities and asked for their written consent to be interviewed. Additional file 2 contains the exit interview questionnaire. Eligible clients for this analysis were visiting for delivery (5%); post-abortion care (1%) (n = 113 delivery/post-abortion); postnatal care (n = 136; 5%); and immunization services^a^ (n = 545; 21%). The remaining clients were predominately visiting for antenatal care (27%), family planning (18%), curative services (20%), child growth monitoring (2%), and sexually transmitted infection counseling and treatment (1%). Clients of all reproductive ages were included; those who were between the ages of 15–17 and visiting for FP or reproductive health purposes were considered to be emancipated minors who could provide consent to participate in the study. For the analysis of postpartum contraceptive use among women in the household sample, the main outcome is current modern FP method use. Modern methods include: sterilization, intrauterine device, injections, implant, pills, male condom, female condom, emergency contraception, lactational amenorrhea, and spermicides. The outcome variable was coded as a binary variable – ‘yes’ for women who reported currently using any of these methods and ‘no’ for those who reported no current use of any of the methods.

The independent variables of interest to examine the role of service integration on current modern FP use are: ‘received information on family planning before or after delivery’ and ‘received information on family planning at an immunization appointment.’ The integration around the time of delivery question had the following response categories: delivered at home; delivered in a facility and did not receive family planning information; and delivered in a facility and received family planning information. The response categories for the integration at immunization question were: did not go for any vaccination visits for youngest child; went for vaccination visits but did not receive FP information; went for vaccination visits and received FP information.

Also included in this analysis are the key constructs from the Health Belief Model including perceived susceptibility, perceived barriers, cues to action, and self-efficacy [9]. We measured perceived susceptibility as whether the woman reported correct knowledge of the fertile period (i.e., risk period is the middle of the cycle). Perceived barrier to family planning use was measured by whether the woman reported that she would be shunned by community members if she used FP. The cue to action measure was created based on the duration since the last birth (<6 months, 6–11 months, 12–17 months, and 18–23 months). Finally, the self-efficacy measure was created based on 8 questions on whether the woman felt confident starting a conversation on FP; convincing her partner to use FP; getting to a place to obtain FP; obtaining a method if she decided to use one; using a method even if her partner did not want to; using a method of FP even if her friends did not; using a method of FP if her religious leader told her not to; and continuing to use a method even if she experienced side effects.

All models adjusted for age, education, religion, type of union, wealth group, number of living children, and study site (see Table 1 for distributions of these variables). Descriptive statistics and multivariate logistic regression models were performed using Stata statistical software version 12, adjusting for clustering of women within sampling units, and using the survey weights. Ethical approval for the study protocol and informed consent process was obtained from the University of North Carolina at Chapel Hill Institutional Review Board and from the Ministry of Health National Ethics Committee in Senegal.

**Table 1:** Client exposure to family planning information at high volume facilities by type of service received on day of visit among clients in high volume facilities in urban Senegal, 2011

	**Delivery/abortion (n = 113**^**a**^**)**	**Postnatal care (n = 136)**	**Immunization for child (n = 545**^**b**^**)**
Using FP at time of visit (%)	(n = 10^c^)	(n = 136)	(n = 545)
Non-user	100.0	97.8	81.3
Traditional method^d^	0.0	0.0	0.7
Modern user	0.0	2.2	18.0
During your visit, did you receive any FP information? (% yes)	8.9	25.7	5.3
Do you know if you can receive FP information or methods in this facility? (% yes, can receive)	67.3	83.8	77.1
Did you receive a FP method, a referral, or a prescription during your visit today? (% yes)	1.8	2.2	2.0
Among clients who did not receive a method or do not use a method:	(n = 111)	(n = 131)	(n = 478)
If a provider had offered you a method of FP today, would you have accepted? (% yes)	42.3	38.2	49.8
Would you be interested in talking to a provider about FP in the unit where you received your principal service today? (% yes)	81.1	74.8	84.9

^a^Of the 113 clients for these services, 10 were for postabortion care.

^b^Includes non-pregnant women (six women dropped from full immunization seeking sample).

^c^Women who were in for delivery were not asked about current family planning use; all other questions include the full sample of delivery/abortion unless noted otherwise.

^d^Traditional methods include billings method (standard days, safe days) and withdrawal method.

## Results

**Figure 1:**
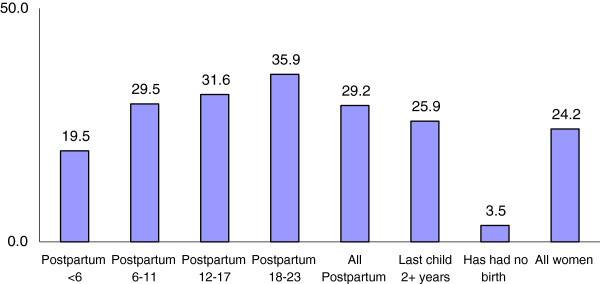
Percentage of women in union using modern contraception by duration postpartum and childbearing experience.

Figure 1 demonstrates that modern family planning (FP) use varies by birth history and postpartum duration. In particular, the women in union who have never had a child in urban Senegal are the least likely to use a modern method of FP (3.5%). Family planning use is highest (36%) among the women who are in the 18–23 months postpartum window. However, this use is low given that 63% of women who are within 2 years postpartum (i.e., 0–23 months postpartum) report that they want to wait another 2 years or more before having another child (not shown). In addition, another 16% want to avoid future childbearing (not shown).

Table 1 presents information from the client exit interviews. Not surprisingly, FP use was highest among clients of immunization services (18%) and low for clients visiting for delivery and postnatal or post-abortion services. All three groups of clients were asked if they received any FP information. Receipt of FP information was highest (26%) among the postnatal care clients and lowest among the immunization clients (5%); nine percent of the delivery/post-abortion clients received information on FP. The majority of clients knew that FP information or methods are available in the facilities they were visiting. Only 2% of clients of any of the services received a FP method, referral or prescription during their visit to the health facility. Notably, among the majority of clients who did not receive a method or referral or prescription, between two-fifths and one-half of the clients reported that if the provider had offered a method of FP they would have accepted and more than three-quarters would have been interested in talking to a provider in the site where they received the service.

Table 2 demonstrates that recently pregnant women in the household sample tend to be young with nearly 80% of the sample under age 34. The women are evenly split by the postpartum duration with about a quarter in each group.

**Table 2:** Descriptive characteristics of married women who had a birth in the last two years among women surveyed in six cities in Senegal, 2011

	Percent*	**Weighted N**	**Unweighted N**
Urban site			
Dakar	36.3	682	289
Guédiawaye	9.0	170	240
Pikine	14.9	280	289
Mbao	25.1	472	232
Mbour	7.2	135	488
Kaolack	7.5	142	482
Age group			
<25	26.8	503	603
25–34	51.4	966	1011
35–39	14.8	278	272
40+	7.1	133	134
Education level			
None	42.7	803	946
Primary	36.2	679	746
Secondary	21.1	397	328
Wealth tertile			
Poorest	38.1	715	864
Medium	33.5	629	704
Highest	28.5	535	452
Religion			
Non-Muslim	5.2	97	63
Muslim	94.8	1,782	1,957
Type of union			
Monogamous	76.2	1,433	1,536
Polygynous	23.8	446	484
Number of children			
0–1	24.6	462	481
2	23.5	442	472
3	20.2	380	390
4	31.7	595	677
Duration postpartum			
<6 months	26.0	488	485
6–11 months	26.4	496	588
12–17 months	23.7	446	436
18–23 months	23.9	449	511
Total	100%	1,879	2,020

*All percentages are weighted.

The key independent variables for the multivariate analysis of modern contraceptive use are presented in Table 3. Overall, among women who are within two years of their last birth, 6% did not deliver in a health facility. About one quarter of women delivered in a health facility and received FP information either before or after delivery (while at the facility). Notably, more than two-thirds of women who had a birth in the last two years delivered in a health facility but did not receive FP information before or after delivery. There is no difference in exposure to FP information before/during delivery by duration postpartum. The majority of women (98%) sought immunization services for their most recent birth; however, 8% of women with a birth within 0–6 months did not seek these services. Among women who obtained immunization services, the overwhelming majority were not offered FP during any of these immunization visits. Only 12% of women sought immunization services and received FP information during at least one of the immunization visits.

**Table 3:** Health belief model key constructs and integration exposure among currently married women by duration postpartum among women in six cities in Senegal, 2011

	**Postpartum women**				
	**<6**	**6 - 11**	**12 - 17**	**18-23**	**Total**
Knowledge of fertile period					
Does not know	79.8	74.9	73.1	75.0	75.8
Knows fertile period	20.2	25.1	26.9	25.0	24.2
Opposition to FP					
No opposition from community	77.5	86.3	78.5	77.3	80.0
Perceived opposition	22.5	13.7	21.5	22.7	20.0
Self-efficacy to use FP (continuous)					
Mean value (1–8)	5.37	5.64	5.72	5.46	5.55
Received FP information before/during delivery					
No	72.4	67.0	70.5	72.8	70.6
Yes	20.1	26.4	26.2	20.9	23.4
Did not deliver in a HF	7.5	6.7	3.3	6.3	6.0
Received FP information during Immunization services					
No	79.1	91.4	88.6	84.6	85.9
Yes	13.0	8.2	10.8	15.1	11.7
Did not seek service	7.9	0.5	0.6	0.3	2.4
N (weighted)	488	496	446	449	1,879

The key health belief model constructs are also presented in Table 3. About one quarter of the women is knowledgeable of their fertile period and there is no difference in this knowledge by the duration postpartum. Only one-fifth of women reported community-level opposition to their potential use of modern FP with no difference by the duration postpartum. In this sample, self-efficacy was moderate with, on average, women reporting confidence that they could do about five of the eight actions.

In Table 4, we present three regression models. Model 1 includes the integration at delivery variable; Model 2 includes the integration at immunization variable, and Model 3 includes both integration variables. Furthermore, Table 4 includes the constructs of the Health Belief Model (HBM). All models adjusted for the demographic variables (not shown). Model 1 demonstrates that women who delivered in a health facility and did not receive FP information are significantly less likely to be using modern FP at the time of the survey than women who delivered in a health facility and received FP information. No difference was found between women who delivered in a facility and received information and women who delivered at home; this may reflect other factors associated with delivering at home (e.g., unexpected delivery and inability to get to facility) and that only a small percentage of women in urban Senegal deliver at home. Model 2 demonstrates that the only integration-related difference is between women who have not visited a facility and women who went to a facility for immunizations (whether or not she received FP information). In particular, women who have not been for immunization visits are significantly less likely to be modern method users than women who went for any immunizations; no difference in contraceptive use is found between women who received FP information during child immunization and those who did not. Model 3 includes both integration variables. The results are similar to what was found in both Models 1 and 2.

**Table 4:** Multivariate logistic regression odds ratios (95% CI) of modern contraceptive use among currently married women who had a birth in the last 2 years and are not currently pregnant, urban Senegal, 2011

	**Model 1**	**Model 2**	**Model 3**
Received FP information before/during delivery			
Delivered at home	0.62 (0.27-1.41)		0.62 (0.26-1.46)
Delivered at facility - no info	0.67 (0.47-0.95)*		0.65 (0.45-0.96)*
Delivered at facility - received information (ref)	1.0		1.0
Received FP information during Immunization services			
Did not seek service		0.07 (0.01-0.31)***	0.08 (0.02-0.38)**
Sought immunization - no info		1.17 (0.76-1.80)	1.33 (0.83-2.14)
Sought immunization - received information (ref)		1.0	1.0
Knowledge of fertile period			
Does not know (ref)	1.0	1.0	1.0
Knows fertile period	1.02 (0.67-1.55)	1.04 (0.69-1.56)	1.03 (0.68-1.57)
Opposition to FP			
No opposition from community	1.0	1.0	1.0
Perceived opposition	0.94 (0.56-1.59)	0.96 (0.57-1.59)	0.94 (0.55-1.59)
Self-efficacy to use FP, mean (SD)	1.51 (1.37-1.67)***	1.54 (1.40-1.71)***	1.53 (1.38-1.69)***
Duration Postpartum			
<6 months	0.28 (0.16-0.49)***	0.31 (0.18-0.53)***	0.31 (0.18-0.53)***
6-11 months	0.44 (0.25-0.77)**	0.44 (0.25-0.77)**	0.42 (0.24-0.74)**
12-17 months	0.56 (0.36-0.89)*	0.57 (0.36-0.91)*	0.55 (0.35-0.88)*
18-23 months (ref)	1.0	1.0	1.0

N = 2020 - unweighted number; 1879 weighted number; Models also adjust for age, education, site, wealth, religion, type of union, and number of children.

+p ≤ 0.10; *p ≤ 0.05; **p ≤ 0.01; ***p ≤ 001.

The results of the constructs of the HBM were consistent across all models shown. Women who had a recent birth (0–17 months) are significantly less likely to be modern method users than women who delivered 18–23 months prior to survey; this is consistent with the pattern found in Figure 1. We also observed that women with higher self-efficacy to use FP are significantly more likely to be using a modern method at the time of the survey. No significant differences are found based on the perceived susceptibility (knowledge of the fertile period) and the perceived barriers (opposition to family planning). The findings for the demographic variables (not shown) are as expected: less educated and poorer women are less likely to use modern family planning whereas women from Guédiawaye and younger women are more likely to use (than women from Dakar and women 35+).

In the analyses presented, women who report lactational amenorrhea method (LAM) as their current contraceptive method are included among the modern method users. Given that LAM is only effective for the first six months if a woman is exclusively breastfeeding, we explored the same models setting the fourteen women (less than 1% of the sample) who were using this method as non-modern method users. In results not shown, the findings are the same with no change in the direction or significance of any of the variables in the model.

## Discussion

This study aims to examine the role of integration of family planning services into MCH services as a way of improving access to postpartum FP use in urban Senegal. We find that delivery and immunization services are missed opportunities for integrated services as the majority of postpartum women (those who delivered in a facility and those who sought child immunization services) did not receive family planning information or counseling and are not using any FP method at the time of these visits. These results emphasize the gap in FP service provision in urban Senegal. Other studies from sub-Saharan Africa (Nigeria and Kenya) have also found similar results [6,21]. Hence, there is evidence for the need to focus resources on integrating FP services into MCH programs as a way to improve access to FP services. Furthermore, the finding that many postnatal and immunization clients would have accepted a contraceptive method if offered at the time of clinic visit shows that women’s need for family planning is not being met. Efforts should be made to ensure that providers identify and fulfill the contraceptive needs of their clients.

The HBM has been previously applied to studies on FP use [8] and consistently explains the importance of self-efficacy in the use of FP [22,23]. Our study results are consistent as we found that higher self-efficacy scores were associated with greater odds of using a modern method at the time of survey. Another construct of the HBM that is statistically significant in our study is cue to action measured by the duration postpartum. We find that the shorter the postpartum period, the less likely the women are to use modern FP. Although previous studies have shown that women who do not know their fertile period are less likely to use FP during the postpartum period, this association is not significant in our study. This finding may reflect low overall knowledge of the postpartum fertile period in this sample. The statistically insignificant association between perceived barriers (measured as opposition to use) and use of modern FP could be due to the fact that the majority of the women did not perceive opposition from the community.

We cannot fail to acknowledge the shortcomings of our study. One such limitation is the potential for recall bias. However, we estimate that the effect of this bias on our estimates will be minimal, if any, as the study is restricted to the woman’s last birth which occurred within the 23 months prior to survey. Another limitation of the study is the fact that it is a cross-sectional study and thus it is not possible to know the direction of causality. For example, some women may adopt a method and then report higher self-efficacy rather than self-efficacy leading to greater contraceptive use. Next, it is possible that some women were surveyed in both the household survey and the facility-level survey. The probability of this is low. Given that the data from these sources are being presented separately and capturing different constructs, the effect of this possible overlap is considered minimal. Finally, the information in the client exit interviews about the willingness to accept a method is hypothetical; it is possible that the women are giving socially acceptable responses and might not actually adopt a method if offered. Future studies that offer family planning information and services to postnatal and immunization clients to estimate the true missed opportunities in these settings are needed.

## Conclusion

Despite these limitations, the study results have important programmatic implications. First, we demonstrate that integrating family planning into delivery/postpartum services in high volume settings is associated with greater postpartum FP use. Family planning programs in urban Senegal should train all delivery/postpartum service providers (doctors, nurses, midwives, matrons, etc.) in counseling and provision of modern FP methods at this important juncture with postpartum women. These providers can offer postpartum FP in health facilities as well as through outreach services. As demonstrated, receipt of this information postpartum is not widely happening and this is a gap in current postpartum service provision. Outreach programs to postpartum women that increase women’s self-efficacy to use modern FP may also lead to increases in modern family planning use, assuming that these women have appropriate methods easily accessible either at a health facility or a pharmacy once they decide to use a method. Future research is needed, perhaps using operations research studies, to determine the feasibility and efficacy of offering FP services to women at immunization visits, postnatal care visits, and child growth monitoring visits. Integration at the time of immunization is not a common practice in urban Senegal but merits further study to determine if it is a cost-effective integration point in this setting. Integrating FP into maternal and child health services is a promising practice but requires additional evidence before being expanded widely. Finding the right integration approaches and strategies can go a long way to ensure that all women in urban Senegal and beyond are able to live healthy lives and raise healthy children.

## Endnotes

^a^The women visiting the facility for immunization services may have children that are more than two-years old since age of the child was not determined in the exit interview.

## Competing interests

The authors declare that they have no competing interests.

## Authors’ contributions

ISS and JCF conceived of the study; CF and JCF developed the recoded data set; ISS performed the analyses; ISS and CO wrote the initial draft; CS provided programmatic implications; all authors reviewed and approved the final draft of the paper.

## Authors’ information

At the time this article was developed JCF was working with the African Population Health and Research Center.

## Pre-publication history

The pre-publication history for this paper can be accessed here:

http://www.biomedcentral.com/1471-2458/13/752/prepub

## Supplementary Material

Additional file 1 Questionnaire Femme. Click here for file

Additional file 2 Questionnaire Cliente A La Sortie Des Structures Sanitaires. Click here for file
