# Supplementary material for: Influence of integrated services on postpartum family planning use: a cross-sectional survey from urban Senegal
Source: BMC Public Health. 2013 Aug 14;13:752. doi: 10.1186/1471-2458-13-752 (PMC3846684; doi:10.1186/1471-2458-13-752)
Supplement: Additional file 1 — Questionnaire Femme. [file 1471-2458-13-752-S1.pdf]


SITE | GRAPPE | N° MEN | N°LIGNE

MLE PROJECT SENEGAL

CRDH
Centre de Recherche pour le Développement Humain

Étude de Base auprès des Ménages et des Points de Prestation de services à l'intention de Mesure, Apprentissage et Évaluation (MLE) et de l'Initiative Sénégalaise en Santé Urbaine (ISSU) dans six villes du Sénégal. 2011

QUESTIONNAIRE FEMME

STRICTEMENT CONFIDENTIEL

MESURE APPRENTISSAGE ET EVALUATION (MLE) DE L'INITIATIVE SENEGALAISE EN SANTE URBAINE (ISSU)
ENQUETE DE BASE (2011)

QUESTIONNAIRE FEMME

SITE	GRAPPE			N° MEN			N°LIGNE	

REPUBLIQUE DU SENEGAL
MINISTERE DE LA SANTE ET DE LA PREVENTION

CRDH

IDENTIFICATION

SITE ________	SITE…………………… ☐
(DAKAR=1, GUEDIAWAYE=2, PIKINE=3, MBAO=4, MBOUR=5, KAOLACK=6)	
DISTRICT SANITAIRE…………………………	DS………………… ☐☐
NOM DU QUARTIER ________	
NUMÉRO DE GRAPPE…………………………	GRAPPE…… ☐☐☐
STATUT DE LA GRAPPE (PAUVRE=1, NON PAUVRE=0)…………………	STATUT……………… ☐
NUMÉRO DE CONCESSION…………………………	N° CONC….. ☐☐☐
NUMÉRO DE MÉNAGE…………………………	N° MÉN……… ☐☐☐
NOM ET NUMÉRO DE LIGNE DE LA FEMME ________	N° LIGNE……… ☐☐

VISITES POUR L'INTERVIEW

	1	2	3	VISITE FINALE
DATE	________	________	________	JOUR .... ☐☐<br>MOIS .... ☐☐<br>ANNÉE 2 0 1 1
RÉSULTAT*	☐	☐	☐	CODE RÉSULTAT……… ☐
NOM DE L'ENQUÊTRICE	________	________	________	
CODE	________	________	________	CODE ENQ... ☐☐☐
PROCHAINE VISITE DATE<br>HEURE	________<br>________	________<br>________		NBRE TOTAL DE VISITES……………. ☐

*CODES RÉSULTAT :
1 REMPLI
2 PAS À LA MAISON
3 DIFFÉRÉ
4 REFUSÉ
5 REMPLI PARTIELLEMENT
6 INCAPACITÉ
7 AUTRE ________ (PRÉCISER)

LANGUE DE QUESTIONNAIRE** ________ 1

LANGUE DE L'INTERVIEW** ________ ☐☐

**CODES LANGUE :
01 FRANÇAIS
02 WOLOF
03 POULAR
04 SERER
05 DIOLA
06 MANDINGUE
96 AUTRES ________

RECOURS A UN INTERPRÈTE : (OUI=1, NON=2) ☐

CONTRÔLEUSE	CHEF D'ÉQUIPE	CONTRÔLE BUREAU	SAISI PAR
NOM ________	NOM ________	NOM ________	NOM ________
CODE………… ☐☐☐	CODE………… ☐☐☐	CODE….. ☐☐☐	CODE. ☐☐☐
DATE ________	DATE ________	DATE ________	DATE ________

SITE | GRAPPE | N° MEN | N°LIGNE

Lettre d'information de la femme dans l'enquête ménage

Nom et prénoms de la femme : ____

Madame,

Mon nom est_______________________ et je travaille comme enquêteur/chef d'équipe dans une étude commanditée par Intrahealth pour le compte du Ministère de la Santé et de la Prévention et réalisée par le CRDH (Centre de Recherche pour le Développement Humain).

Il vous est proposé de participer à une enquête sur la santé maternelle et infantile qui vise à améliorer la qualité de vie des populations urbaines des villes sénégalaises à travers l'augmentation de l'accès et l'utilisation des services de santé de la reproduction. Si vous acceptez de participer, vous ferez partie des 14 000 personnes qui seront interrogées dans le cadre de cette étude Les informations que vous allez nous fournir seront très utiles au gouvernement pour planifier les politiques de santé et répondre aux besoins non satisfaits en santé de la reproduction. Votre localité a été choisie au hasard parmi d'autres de votre région.

Vous serez interviewé par une enquêtrice/enquêteur. Des informations sur l'accès et l'utilisation des services de santé, sur la santé maternelle et infantile, sur votre couple ainsi que des questions d'opinions et d'attitudes vous seront demandées.

Dans le cadre du suivi des résultats de l'étude, il est prévu de s'entretenir avec les mêmes femmes à des intervalles de deux et quatre ans. Pour ce faire, nous allons recueillir pour chaque foyer et chaque femme éligible des informations pertinentes afin de pouvoir les contacter ultérieurement. Ces informations porteront sur les noms, les adresses et les numéros de téléphone (mobile ou fixe) des personnes enquêtées et de leurs proches. Les informations obtenues seront sur un formulaire de suivi qui sera séparé de l'étude après avoir été rempli.

L'entretien prend généralement entre 30 et 90 minutes. Nous avons pris les dispositions idoines pour que toute information que vous nous fournirez reste strictement secrète. L'entretien va être confidentiel et se tiendra en privé. Si les conditions pour que l'entretien se tienne en privé ne sont pas réunies, nous allons arrêter l'interview jusqu'à ce que celles-ci le soient. L'exploitation de l'information sera et restera anonyme.

Vous ne tirerez pas d'avantages directs de cette enquête, mais les informations que vous allez nous fournir aideront le Gouvernement à planifier les actions futures dans le domaine de la santé de la reproduction.

Il n'y a pas d'inconvénients à participer à cette enquête, hormis le fait de rester chez vous et de répondre aux questions posées. Aucun prélèvement ou traitement n'est envisagé dans le cadre de cette enquête. La participation à cette enquête est volontaire; s'il y a une question à laquelle vous ne voulez pas répondre, faites-le savoir et l'enquêteur passera à la question suivante. Vous pouvez également renoncer à l'entretien à tout moment. Nous espérons cependant que vous participerez à cette enquête car votre opinion est particulièrement importante pour nous.

Si vous acceptez de participer, vous devez signer ce document pour signifier que vous avez reçu l'information et marquer votre accord. Une copie du document signé vous sera remise.
Vous pouvez contacter le Directeur du Centre de Recherche pour le Développement Humain, HLM Hann Mariste, Im. A, # 7A/7B. Téléphone : 33 832 63 79, à tout moment si vous avez des questions au sujet de l'enquête.

Vous pouvez contacter Dr. Samba Cor Sarr, Direction de la Santé, MSP, 1 Rue Aimé Césaire, 2ème étage, Dakar Fann. BP 4024, Tél : 33 869 43 13, à tout moment si vous avez des questions au sujet de vos droits en tant que participant à l'enquête.

Votre participation à cette enquête est volontaire, ni votre entourage ni vous-même, ne serez pénalisés, ni ne perdrez aucun de vos droits si vous décidez d'arrêter votre participation.

Fait à_______________, le _______________

Signature de l'enquêteur/trice

SITE | GRAPPE | N° MEN | N°LIGNE

Fiche de consentement libre et éclairé de la femme dans l'enquête ménage

Je soussigné Mme/Mlle ____________________________ atteste avoir reçu la lettre d'information contenant les explications détaillées sur le déroulement de l'enquête et les conditions de ma participation et avoir reçu une réponse satisfaisante à toutes les questions que j'ai posées.

Dans le cadre du suivi des résultats de l'étude, je serai recontactée à des intervalles de deux et quatre ans après cette enquête.

Certifie avoir donné mon accord volontaire et libre de participer à l'enquête.

Je retiens garder mon droit de suspendre ma participation à cette étude à tout moment et sans aucun préjudice.

Si j'ai des questions au sujet de l'enquête, je peux contacter à tout moment le Directeur du Centre de Recherche pour le Développement Humain, Téléphone : 33 832 63 79.

Au sujet des questions sur mes droits en tant que participant à l'enquête, je peux à tout moment contacter Dr. Samba Cor Sarr, Direction de la Santé, MSP, 1 Rue Aimé Césaire, 2ème étage, Dakar Fann. BP 4024, Tél : 33 869 43 13.

En foi de quoi, j'accepte de signer cette fiche de consentement pour servir et valoir ce que de droit.

Fait à_______________, le _______________ Fait à_______________, le _______________

Signature de l'enquêteur/trice Signature Participante

SECTION 1. CARACTÉRISTIQUES SOCIODÉMOGRAPHIQUES DE L'ENQUÊTÉE

N°	QUESTIONS ET FILTRES	CODES	ALLER À
	ENREGISTRER L'HEURE.	HEURE . . . . [ ][ ]<br>MINUTES . . . . [ ][ ]	
	Merci d'avoir accepté de participer à l'enquête. Comme je l'ai mentionné lorsque je vous ai demandé votre consentement, nous cherchons à évaluer vos besoins en santé et en informations. Pour commencer, nous allons vous poser des questions de base sur vous-mêmes.		
Q101	En quel mois et en quelle année êtes-vous née ?	MOIS . . . . [ ][ ]<br>NE CONNAÎT PAS LE MOIS . . . . 98<br>ANNÉE . . . . [ ][ ][ ][ ]<br>NE CONNAÎT PAS L'ANNÉE 9998	
Q102	Quel âge aviez-vous à votre dernier anniversaire ?<br>COMPARER ET CORRIGER 101 ET/OU 102 SI INCOHÉRENT.	ÂGE EN ANNÉES RÉVOLUES [ ][ ]	
Q103	**VERIFIER 101 ET 102: A-T-ELLE ENTRE 15 ET 49 ANS?**<br>OUI: [ ] ↓ NON: [ ] →<br>VERIFIER QUE LE CONSENTEMENT A ETE SIGNE. SINON OBTENIR LE CONSENTEMENT. ↓		FIN INTERVIEW
Q104	Avez-vous déjà fréquenté l'école?	OUI . . . . 1<br>NON 2	 → Q109
Q105	Quel est le plus haut niveau d'études que vous avez atteint : primaire, secondaire ou supérieur ?	PRIMAIRE.. 1<br>SECONDAIRE 1 .... 2<br>SECONDAIRE 2 .... 3<br>SUPERIEUR...................................... 4	
Q106	Quelle est (l'année/classe) la plus élevée que vous avez achevée à ce niveau ?<br>SI MOINS D'UNE ANNEE A ETE ACHEVEE A CE NIVEAU, INSCRIRE '00'.	CLASSE/ANNÉE . . . . [ ][ ]	
Q107	**VÉRIFIER 105:**<br>PRIMAIRE: (Q105=1) [ ] ↓ SECONDAIRE OU SUPERIEUR: (Q105 = 2, 3, OU 4) [ ] →		Q110

Nº	QUESTIONS ET FILTRES	CODES	ALLER À
Q109	Maintenant, je voudrais vous demander de me lire cette phrase.<br><br>MONTRER A L'ENQUETEE UNE PHRASE FIGURANT SUR LA CARTE D'ALPHABETISATION.<br><br>SI L'ENQUETEE NE PEUT LIRE TOUTE LA PHRASE, INSISTER:<br>Pouvez-vous me lire certaines parties de la phrase ?	NE PEUT PAS LIRE DU TOUT . . . . 1<br>PEUT SEULEMENT LIRE DES<br>PARTIES DE LA PHRASE……………. 2<br>PEUT LIRE TOUTE LA PHRASE………… 3<br>PAS DE CARTE DANS<br>LA LANGUE QUI CONVIENT______<br>________________________ 4<br>(PRÉCISER LA LANGUE)<br>AVEUGLE/MALVOYANT…………………. 5	
Q110	Quelle est votre religion?	CATHOLIQUE……………………………. 01<br>AUTRE RELIGION CHRETIENNE…….. 02<br>MUSULMAN…………………………………. 03<br>TRADITIONNELLE………………………. 04<br>AUCUNE RELIGION……………………. 05<br>AUTRE__________________________ 96<br>(PRECISER)	05 → SQ1
Q111	Dans quelle mesure vos croyances religieuses influencent-elles vos décisions en matière de PF ? Diriez vous: Pas du tout, quelques fois, souvent ou toujours?	PAS DU TOUT…………………………….. 1<br>QUELQUEFOIS…………………………… 2<br>SOUVENT……………………………….. 3<br>TOUJOURS………………………………. 4<br>NE CONNAIT PAS LA PF………………… 8	
SQ1	Etes-vous sénégalaise ?	OUI . . . . 1<br>NON 2	2 → Q201
Q112	Quel est votre groupe ethnique ?	WOLOF/LEBOU……………………………. 01<br>POULAR . . . . 02<br>SERER . . . . 03<br>DIOLA……………………………………. 04<br>MANDINGUE……………………………. 05<br>SONINKÉ…………………………………. 06<br>AUTRE ________________________ 96<br>(PRÉCISER)	

SECTION 2. REPRODUCTION

Nº	QUESTIONS ET FILTRES	CODES	ALLER À
	Maintenant, je voudrais vous poser des questions sur toutes les naissances que vous avez eues au cours de votre vie. S'il vous plaît, soyez le plus précis possible et sachez que vos réponses ne seront communiquées à personne d'autre.		
Q201	Avez-vous déjà donné naissance à des enfants ?	OUI . . . . . . 1<br>NON 2	 → Q206
Q202	Y a-t-il des fils ou des filles à qui vous avez donné naissance qui vivent actuellement avec vous?	OUI . . . . . . 1<br>NON 2	 → Q204
Q203	Combien de fils vivent avec vous ?<br>Et combien de filles vivent avec vous?	FILS À LA MAISON . . . . . [ ][ ]<br>FILLES A LA MAISON . . . . . [ ][ ]	
Q204	Y a t-il des fils ou des filles à qui vous avez donné naissance et qui sont toujours en vie, mais qui ne vivent pas avec vous ?	OUI . . . . . . 1<br>NON 2	 → Q206
Q205	Combien de fils sont en vie mais ne vivent pas avec vous?<br>Combien de filles sont en vie mais ne vivent pas avec vous ?	FILS AILLEURS . . . . . [ ][ ]<br>FILLES AILLEURS . . . . . [ ][ ]	
Q206	Avez-vous déjà donné naissance à un fils vivant ou à une fille vivante mais qui est décédé/e plus tard ?<br>SI NON INSISTER : Aucun bébé ayant crié ou montré un signe de vie, mais qui n'a pas survécu ?	OUI . . . . . . 1<br>NON 2	 → Q208
Q207	Combien de fils sont décédés?<br>Et combien de filles sont décédées?	FILS DÉCÉDÉS . . . . . [ ][ ]<br>FILLES DÉCÉDÉES . . . . . [ ][ ]	
Q208	FAIRE LA SOMME DES RÉPONSES À 203, 205, ET 207, ET INSCRIRE LE TOTAL.<br>S'IL N'Y EN A AUCUN, INSCRIRE '00'.	TOTAL DES NAISSANCES..... [ ][ ]	
Q209	**VERIFIER Q208:**<br>Je voudrais tout juste m'assurer que j'ai bien compris: Vous avez eu au TOTAL ------------- naissances dans votre vie.<br>Est-ce bien exact?<br>OUI [ ] ↓ NON [ ] → INSISTER ET CORRIGER 201 A 208 COMME IL SE DOIT.		
Q210	Avez-vous eu une grossesse qui s'est achevée par une fausse couche ou un avortement ? Par fausse couche ou avortement, j'entends une grossesse n'arrivant pas à terme, soit parce que vous avez fait quelque chose pour l'arrêter, soit parce qu'elle s'est arrêtée toute seule (naturellement).	OUI . . . . . . 1<br>NON 2	 → Q212

Q211	Quand est-ce qu'une telle grossesse avait-elle pris fin pour la <u>dernière fois</u>?	MOIS . . . . [ ][ ]<br>NE CONNAIT PAS MOIS……………… 98<br><br>ANNEE…………….. [ ][ ][ ][ ]<br>NE CONNAIT PAS L'ANNEE 9998	
Q212	Avez-vous eu une grossesse qui s'est achevée par un <u>mort-né</u> ? Par mort-né, j'entends un bébé né <u>à terme</u> mais qui n'a donné aucun signe de vie ?	OUI . . . . 1<br>NON 2	 Q214
Q213	Quand est-ce qu'une telle grossesse avait-elle pris fin pour la <u>dernière fois</u>?	MOIS . . . . [ ][ ]<br>NE CONNAIT PAS MOIS……………… 98<br><br>ANNEE…………….. [ ][ ][ ][ ]<br>NE CONNAIT PAS L'ANNEE 9998	
Q214	**VERIFIER Q208, Q210 ET Q212:**<br><br>UNE OU PLUSIEURS NAISSANCES (Q208 >= 1)<br>AVORTEMENTS, FAUSSES COUCHES (Q210=1)<br>OU MORTS-NES (Q212=1): [ ] ↓<br><br>AUCUN :<br>(Q208=0 ET Q210=2 ET Q212=2) [ ]		Q235
Q215	Quel âge aviez-vous à votre <u>toute première</u> grossesse ?	AGE (ANS REVOLUS)……….. [ ][ ]	
Q216	Au moment où vous êtes tombée enceinte pour la première fois, vouliez-vous tomber enceinte à ce moment-là, vouliez-vous attendre plus tard, ou vouliez-vous ne pas avoir d'enfants ?	À CE MOMENT-LÀ ……………………….. 1<br>PLUS TARD ………………………………. 2<br>NE PAS AVOIR D'ENFANT ………………. 3	Q217A<br><br>Q217A
Q217	Combien de temps auriez-vous souhaité attendre ?<br><br>SI LA REPONSE CONTIENT UNE PARTIE DECIMALE POUR ANNEE (2,5 ANS), CONVERTIR EN MOIS.	MOIS ………………………….. **1** [ ][ ]<br>OU BIEN<br>ANNEES……………………….. **2** [ ][ ]<br>OU BIEN<br><br>AUTRE_____________________ 996<br>(PRECISER)<br>OU BIEN<br>NSP……………………………………… 998	
Q217A	**VÉRIFIER 208:**<br>UNE NAISSANCE OU PLUS [ ] ↓<br><br>AUCUNE [ ]		Q235

Maintenant je voudrais faire la liste de toutes vos naissances, qu'elles soient encore en vie ou non, en commençant par la première que vous avez eue.
ENREGISTRER LES NOMS DE TOUTES LES NAISSANCES COMPTES A Q209 DANS Q218. ENREGISTRER LES NOMS DES JUMEAUX ET DES TRIPLETS SUR DES LIGNES SEPAREES (S'IL Y A PLUS DE 14 ENFANTS, UTILISER UN QUESTIONNAIRE SUPPLEMENTAIRE EN COMMENÇANT PAR LA SECONDE LIGNE).

ENCERCLEZ LE NOM DE TOUS LES ENFANTS NES EN 2009 OU APRES.

Q218<br>Quel nom a été donné à votre (premier enfant/ enfant suivant) ?<br>INSCRIRE LE NOM.	Q219<br>(NOM) est-il/elle une naissance simple ou multiple ?	Q220<br>(NOM) est-il/elle un garçon ou une fille?	Q221<br>En quel mois et quelle année (NOM) est-il/elle né(e) ?<br>INSISTER : Quel est son anniversaire? (autres manières d'insister: c'était en quelle saison?)<br>NSP MOIS: " 98"	Q222<br>(NOM) est-il/elle toujours en vie ?	Q223<br>SI EN VIE :<br>Quel âge avait (NOM) à son dernier anniversaire ?<br>INSCRIRE L'ÂGE EN ANNÉES RÉVOLUES. SI MOINS DE 1 AN, ENREGISTRER 00.	Q224<br>SI EN VIE:<br>(NOM) vit-il/elle avec vous ?	Q225<br>SI DÉCÉDÉ :<br>Quel âge avait (NOM) au moment de son décès?<br>De combien de mois était âgé (NOM)?<br>SI MOINS D'1 MOIS ; ENREGISTRER EN JOURS;<br>SI MOINS DE 2 ANS ; ENREGISTRER EN MOIS;<br>AUTRES CAS, EN ANNEES.	Q226<br>Y a t-il eu des naissances vivantes entre (NOM DE L'ENFANT PRECEDENT) et (NOM), y compris d'autres enfants décédés après la naissance ?
01	SIMPLE. 1<br>MULT 2	GARC. 1<br>FILLE. 2	MOIS ☐☐<br>ANNÉE ☐☐☐☐	OUI 1<br>NON 2<br>↓ 225	ÂGE EN ANNÉES ☐☐	OUI . . . 1<br>ENFT SVT ←<br>NON...... 2	JOURS 1<br>MOIS 2<br>ANNÉES 3<br>☐☐	
02	SIMPLE. 1<br>MULT 2	GARC. 1<br>FILLE. 2	MOIS ☐☐<br>ANNÉE ☐☐☐☐	OUI 1<br>NON 2<br>↓ 225	ÂGE EN ANNÉES ☐☐	OUI . . . 1<br>Q226 ←<br>NON...... 2	JOURS 1<br>MOIS 2<br>ANNÉES 3<br>☐☐	OUI . . . 1<br>AJOUTEZ NAISS.<br>NON . . . 2<br>NAISS. SUIVANTE
03	SIMPLE. 1<br>MULT 2	GARC. 1<br>FILLE. 2	MOIS ☐☐<br>ANNÉE ☐☐☐☐	OUI 1<br>NON 2<br>↓ 225	ÂGE EN ANNÉES ☐☐	OUI . . . 1<br>Q226 ←<br>NON...... 2	JOURS 1<br>MOIS 2<br>ANNÉES 3<br>☐☐	OUI . . . 1<br>AJOUTEZ NAISS.<br>NON . . . 2<br>NAISS. SUIVANTE
04	SIMPLE. 1<br>MULT 2	GARC. 1<br>FILLE. 2	MOIS ☐☐<br>ANNÉE ☐☐☐☐	OUI 1<br>NON 2<br>↓ 225	ÂGE EN ANNÉES ☐☐	OUI . . . 1<br>Q226 ←<br>NON...... 2	JOURS 1<br>MOIS 2<br>ANNÉES 3<br>☐☐	OUI . . . 1<br>AJOUTEZ NAISS.<br>NON . . . 2<br>NAISS. SUIVANTE
05	SIMPLE. 1<br>MULT 2	GARC. 1<br>FILLE. 2	MOIS ☐☐<br>ANNÉE ☐☐☐☐	OUI 1<br>NON 2<br>↓ 225	ÂGE EN ANNÉES ☐☐	OUI . . . 1<br>Q226 ←<br>NON...... 2	JOURS 1<br>MOIS 2<br>ANNÉES 3<br>☐☐	OUI . . . 1<br>AJOUTEZ NAISS.<br>NON . . . 2<br>NAISS. SUIVANTE
06	SIMPLE. 1<br>MULT 2	GARC. 1<br>FILLE. 2	MOIS ☐☐<br>ANNÉE ☐☐☐☐	OUI 1<br>NON 2<br>↓ 225	ÂGE EN ANNÉES ☐☐	OUI . . . 1<br>Q226 ←<br>NON...... 2	JOURS 1<br>MOIS 2<br>ANNÉES 3<br>☐☐	OUI . . . 1<br>AJOUTEZ NAISS.<br>NON . . . 2<br>NAISS. SUIVANTE
07	SIMPLE. 1<br>MULT 2	GARC. 1<br>FILLE. 2	MOIS ☐☐<br>ANNÉE ☐☐☐☐	OUI 1<br>NON 2<br>↓ 225	ÂGE EN ANNÉES ☐☐	OUI . . . 1<br>Q226 ←<br>NON...... 2	JOURS 1<br>MOIS 2<br>ANNÉES 3<br>☐☐	OUI .... 1<br>AJOUTEZ NAISS.<br>NON..... ... . . . 2<br>NAISS. SUIVANTE

Maintenant je voudrais faire la liste de toutes vos naissances, qu'elles soient encore en vie ou non, en commençant par la première que vous avez eue.
ENREGISTRER LES NOMS DE TOUTES LES NAISSANCES COMPTES A Q209 DANS Q218. ENREGISTRER LES NOMS DES JUMEAUX ET DES TRIPLETS SUR DES LIGNES SEPAREES (S'IL Y A PLUS DE 14 ENFANTS, UTILISER UN QUESTIONNAIRE SUPPLEMENTAIRE EN COMMENÇANT PAR LA SECONDE LIGNE).

ENCERCLEZ LE NOM DE TOUS LES ENFANTS NES EN 2009 OU APRES.

Q218	Q219	Q220	Q221	Q222	Q223	Q224	Q225	Q226
Quel nom a été donné à votre (premier enfant/ enfant suivant) ?<br><br>INSCRIRE LE NOM.	(NOM) est-il/elle une naissance simple ou multiple ?	(NOM) est-il/elle un garçon ou une fille?	En quel mois et quelle année (NOM) est-il/elle né(e) ?<br><br>INSISTER :<br>Quel est son anniversaire?<br>(autres manières d'insister: c'était en quelle saison?)<br>NSP MOIS: " 98"<br>NSP ANNEES: " 9998	(NOM) est-il/elle toujours en vie ?	SI EN VIE :<br>Quel âge avait (NOM) à son dernier anniversaire ?<br><br>INSCRIRE L'ÂGE EN ANNÉES RÉVOLUES.<br>SI MOINS DE 1 AN, ENREGISTRER 00.	SI EN VIE:<br>(NOM) vit-il/elle avec vous ?	SI DÉCÉDÉ :<br>Quel âge avait (NOM) au moment de son décès?<br><br>De combien de mois était âgé (NOM)?<br><br>SI MOINS D'1 MOIS ; ENREGISTRER EN JOURS;<br><br>SI MOINS DE 2 ANS ; ENREGISTRER EN MOIS;<br><br>AUTRES CAS, EN ANNEES.	Y a t-il eu des naissances vivantes entre (NOM DE L'ENFANT PRECEDENT) et (NOM), y compris d'autres enfants décédés après la naissance ?
08	SIMPLE. 1<br>MULT 2	GARC. 1<br>FILLE. 2	MOIS<br>ANNÉE	OUI 1<br>NON 2<br>225	ÂGE EN ANNÉES	OUI . . . 1<br>Q226<br>NON…… 2	JOURS 1<br>MOIS 2<br>ANNÉES 3	OUI . . . 1<br>AJOUTEZ NAISS.<br>NON . . . 2<br>NAISS. SUIVANTE
09	SIMPLE. 1<br>MULT 2	GARC. 1<br>FILLE. 2	MOIS<br>ANNÉE	OUI 1<br>NON 2<br>225	ÂGE EN ANNÉES	OUI . . . 1<br>Q226<br>NON…… 2	JOURS 1<br>MOIS 2<br>ANNÉES 3	OUI . . . 1<br>AJOUTEZ NAISS.<br>NON . . . 2<br>NAISS. SUIVANTE
10	SIMPLE. 1<br>MULT 2	GARC. 1<br>FILLE. 2	MOIS<br>ANNÉE	OUI 1<br>NON 2<br>225	ÂGE EN ANNÉES	OUI . . . 1<br>Q226<br>NON…… 2	JOURS 1<br>MOIS 2<br>ANNÉES 3	OUI . . . 1<br>AJOUTEZ NAISS.<br>NON . . . 2<br>NAISS. SUIVANTE
11	SIMPLE. 1<br>MULT 2	GARC. 1<br>FILLE. 2	MOIS<br>ANNÉE	OUI 1<br>NON 2<br>225	ÂGE EN ANNÉES	OUI . . . 1<br>Q226<br>NON…… 2	JOURS 1<br>MOIS 2<br>ANNÉES 3	OUI . . . 1<br>AJOUTEZ NAISS.<br>NON . . . 2<br>NAISS. SUIVANTE
12	SIMPLE. 1<br>MULT 2	GARC. 1<br>FILLE. 2	MOIS<br>ANNÉE	OUI 1<br>NON 2<br>225	ÂGE EN ANNÉES	OUI . . . 1<br>Q226<br>NON…… 2	JOURS 1<br>MOIS 2<br>ANNÉES 3	OUI . . . 1<br>AJOUTEZ NAISS.<br>NON . . . 2<br>NAISS. SUIVANTE
13	SIMPLE. 1<br>MULT 2	GARC. 1<br>FILLE. 2	MOIS<br>ANNÉE	OUI 1<br>NON 2<br>225	ÂGE EN ANNÉES	OUI . . . 1<br>Q226<br>NON…… 2	JOURS 1<br>MOIS 2<br>ANNÉES 3	OUI . . . 1<br>AJOUTEZ NAISS.<br>NON . . . 2<br>NAISS. SUIVANTE
14	SIMPLE. 1<br>MULT 2	GARC. 1<br>FILLE. 2	MOIS<br>ANNÉE	OUI 1<br>NON 2<br>225	ÂGE EN ANNÉES	OUI . . . 1<br>Q226<br>NON 2	JOURS 1<br>MOIS 2<br>ANNÉES 3	OUI . . . 1<br>AJOUTEZ NAISS.<br>NON . . . 2<br>NAISS. SUIVANTE

Q227	Avez-vous eues d'autres naissances vivantes depuis la naissance de (NOM DU DERNIER ENFANT) ?<br><br>SI OUI, ENREGISTRER CETTE/CES NAISSANCE/S DANS LE TABLEAU.	OUI .... 1<br>NON... 2	
Q228	Avant la naissance de (NOM DU PREMIER ENFANT), aviez-vous eues d'autres naissances vivantes ?<br><br>SI OUI, ENREGISTRER CETTE/CES NAISSANCE/S DANS LE TABLEAU.	OUI .... 1<br>NON...... 2	
Q229	**COMPARER Q208 AVEC LE NOMBRE DE NAISSANCES DANS LE TABLEAU CI-DESSUS ET MARQUER :**<br><br>MÊMES NOMBRES: ☐ ↓ NOMBRES DIFFERENTS: ☐ → INSISTER ET CORRIGER<br><br>**VERIFIER:**<br>POUR CHAQUE NAISSANCE: L'ANNEE DE NAISSANCE EST ENREGISTREE: ☐<br>POUR CHAQUE ENFANT VIVANT: L'AGE ACTUEL EST ENREGISTRE: ☐<br>POUR CHAQUE ENFANT DECEDE: L'AGE AU MOMENT DU DECES EST ENREGISTRE ☐<br>POUR L'AGE AU MOMENT DU DECES, 12 MOIS OU 1 AN: SONDEZ POUR DETERMINER LE NOMBRE EXACT DE MOIS: ☐		
Q230	**VERIFIER Q221** ET ENREGISTRER LE NOMBRE DE NAISSANCES EN 2009 OU APRES.<br>NBRE NAISS...... ☐<br>S'IL Y EN A AUCUN, ECRIRE '0'.		
Q231	**VERIFIER Q230:**<br>OUI, A EU UNE OU PLUSIEURS NAISSANCES DEPUIS 2009: ☐ ↓<br>AUCUNE NAISSANCE DEPUIS 2009: ☐ → Q235		Q235
Q232	REPORTER LE NOM ET LE NUMERO DE LIGNE DU DERNIER ENFANT MENTIONNES A Q218:<br>NOM_____________________________________ N° DE LIGNE:………….. ☐☐<br><br>AU CAS OU L'ENFANT EST DECEDE, POSER LES QUESTIONS Q233 et Q234 DE MANIERE APPROPRIEE EN TENANT COMPTE DE LA SENSIBILITE.		
Q233	Lorsque vous êtes tombée enceinte de [NOM CI-DESSUS], souhaitiez-vous tomber enceinte à ce moment-là, souhaitiez-vous attendre plus tard ou souhaitiez-vous ne pas avoir un (autre) enfant du tout ?	A CE MOMENT-LA................................. 1<br>PLUS TARD.. 2<br>NE PAS AVOIR D'ENFANT..................... 3	→ Q235<br><br>→ Q235
Q234	Combien de temps auriez-vous souhaité attendre ?<br><br>SI LA REPONSE CONTIENT UNE PARTIE DECIMALE POUR ANNEE (2.5 ANS), CONVERTIR EN MOIS.	MOIS ……………………………. 1 ☐☐<br>OU BIEN<br>ANNEES………………………… 2 ☐☐<br>OU BIEN<br>AUTRE___________________ 996<br>(PRECISER)<br>OU BIEN<br>NSP……………………………………. 998	
Q235	Etes-vous actuellement enceinte?	OUI... 1<br>NON... 2<br>NE SAIT PAS..................................... 8	 → Q243<br>→ Q243
Q236	De combien de mois êtes-vous enceinte ?	MOIS.................................... ☐☐<br>NE SAIT PAS..................................... 98	
Q237	Vous êtes-vous rendue à un moment quelconque pendant cette grossesse à une visite prénatale ?	OUI... 1<br>NON... 2	 → Q241

Q238	Où est-ce que vous êtes allée la dernière fois pour une visite prénatale?<br><br>INSISTER : Quel est le nom de ce lieu?<br>Où se trouve ce lieu?	NOM:________________________<br>________________________<br>ADRESSE:<br>________________________<br>________________________<br>DECRIRE LE LIEU:_______________<br>________________________<br>________________________	
Q239	Quel est le type de ce lieu?<br><br>UNE SEULE REPONSE EST ENREGISTREE.	**SECTEUR PUBLIC**<br>HOPITAL GOUVERNEMENTAL……… 11<br>CENTRE SANTE GOUVERNEMENTA 12<br>POSTE DE SANTE……...…………… 13<br>STRATEGIE AVANCEE/EQUIPE MOBILE…………………………… 14<br>CENTRE CONSEILS ADOS…….…… 15<br>CASE DE SANTE……………………... 16<br>AUTRE PUBLIC……………………….… 17<br><br>**SECTEUR PRIVE FORMEL**<br>HOPITAL/CLINIQUE/CABINET PRIVE 21<br>PHARMACIE…………………..………….. 22<br>DISPENSAIRE RELIGIEUX…………... 23<br>AUTRE MEDICAL PRIVE……………… 24<br><br>**SECTEUR PRIVE INFORMEL**<br>MEDECIN…………………..…………… 31<br>SAGE-FEMME…………..…………………32<br>INFIRMIER/AI……………..…………… 33<br>MATRONE / ASC.............................. 34<br>GUERISSEUR / ACCOUCHEUSE TRADITIONNELLE…..………………… 35<br>**AUTRE**__________________________ 96<br>(PRECISER)	
Q240	L'endroit où vous avez reçu les soins prénatals, est-ce dans cette ville (le site), une autre ville, ou en zone rurale ?	CETTE VILLE (SITE)……….…..……..... 1<br>UNE AUTRE VILLE………...……………… 2<br>ZONE RURALE……..………………………. 3	
Q241	Au moment où vous êtes tombée enceinte, vouliez-vous tomber enceinte en ce moment-là, vouliez-vous attendre plus tard, ou ne vouliez-vous ne plus/ne pas avoir d enfants ?	EN CE MOMENT-LA……...………………… 1<br>PLUS TARD………………..……..………….. 2<br>PAS DU TOUT……….….……………….. 3	→ Q243<br><br>→ Q243

Q242	Combien de temps de plus auriez-vous souhaité attendre ?<br><br>SI LA REPONSE EST UN NOMBRE DECIMAL DE MOIS OU D'ANNEES, CONVERTIR LE TOUT EN MOIS ET REPORTER DANS LA CASE « MOIS »	MOIS ……………………………. 1<br>OU BIEN<br>ANNEES………………………… 2<br>OU BIEN<br>AUTRE__________________ 996<br>(PRECISER)<br>OU BIEN<br>NSP…………………………………… 998	
Q243	Quand est-ce que vos dernières règles avaient-elles commencé ?<br><br>______________________________<br>(DATE, SI DONNEE)<br><br>SI MOINS D'UN JOUR, ENCERCLER "1" ET ENREGISTRE '00'.	NBRE DE JOURS DEPUIS……. **1** 0 0<br>NBRE DE SEMAINES DEPUIS.. **2**<br>NBRE DE MOIS DEPUIS……... **3**<br>NBRE D'ANNEES DEPUIS……. **4**<br><br>EN MENOPAUSE/A EU UNE HYSTERECTOMIE………………… 994<br>AVANT LA DERNIERE NAISSANCE…. 995<br>N'A JAMAIS EU DE REGLES…………. 996<br>NE SE SOUVIENT PAS………………… 998	
Q244	Entre la période des règles et les règles suivantes, y-a-t-il un moment où une femme a plus de chances de tomber enceinte que d'autres, si elle a des rapports sexuels ?	OUI………………………………………… 1<br>NON………………………………………. 2<br>NE SAIT PAS……………………………… 8	 → Q246<br>→ Q246
Q245	A quelle période du cycle menstruel ces jours corresponden t-ils ? Juste avant le début des règles, Pendant les règles, Juste après la fin des règles, Au milieu entre 2 périodes ?<br><br>UNE SEULE REPONSE EST ENREGISTREE.	JUSTE AVANT LES REGLES……………. 1<br>PENDANT LES REGLES…………………. 2<br>TOUT JUSTE APRES LA FIN DES REGLES 3<br>AU MILIEU ENTRE 2 PERIODES………… 4<br>AUTRE__________________________ 6<br>(PRECISER)<br>NE SAIT PAS……………………………… 8	
Q246	Pensez-vous qu'une femme qui allaite son bébé au sein peut tomber enceinte ?	OUI………………………………………… 1<br>NON………………………………………. 2<br>CELA DEPEND……………………………. 3<br>NE SAIT PAS……………………………… 8	 <br><br>→ Q301
Q247	Dans quelles conditions l'allaitement maternel peut-être considéré comme une méthode contraceptive efficace ?<br><br>INSISTER: Quelque chose d'autre?<br><br>ENREGISTRER TOUT CE QUI EST MENTIONNE.	ALLAITEMENT MATERNEL EXCLUSIF… A<br>ALLAITEMENT MATERNEL EXCLUSIF JUSQU'A 6 MOIS………………………… B<br>AVANT LE RETOUR DE COUCHES……. C<br>AUTRE___________________________ X<br>(PRECISER)	

SECTION 3: CONTRACEPTION

VERIFIER LA PRESENCE D'AUTRES PERSONNES. AVANT DE CONTINUER, FAIRE TOUT POUR GARANTIR UN CADRE INTIME.

Maintenant, je voudrais parler de planification familiale c'est-à-dire des divers moyens ou des diverses méthodes qu'un couple peut utiliser pour retarder ou éviter une grossesse.

Quels sont les moyens ou les méthodes de PF dont vous avez entendu parler?

POUR LES METHODES QUI N'ONT PAS ETE MENTIONNEES SPONTANEMENT, DEMANDER:
Avez-vous déjà entendu parler de (METHODE)?.

ENCERCLER '1' POUR CHAQUE METHODE MENTIONNEE SPONTANEMENT. PUIS PROCEDER EN LISANT DANS LA COLONNE EN BAS LE NOM ET LA DESCRIPTION DE CHAQUE METHODE QUI N'A PAS ETE MENTIONNEE SPONTANEMENT. ENCERCLER '2' POUR CHAQUE METHODE RECONNUE APRES ECOUTE DE LA DESCRIPTION. ENCERCLE '3' POUR CHAQUE METHODE QUI N'A PAS ETE MENTIONNEE NI RECONNUE.

		Q301. Avez-vous déjà entendu parler de (METHODE)?			Q302. Avez-vous déjà utilisé (METHODE)?
		OUI : MEN-TIONNEE SPONTA-NEMENT= **1**	OUI: MEN-TIONNEE APRES DES-CRIPTION= **2**	NON= **3**	
01	STERILISATION FEMININE les femmes peuvent subir une opération pour éviter d'avoir d'autres enfants.	1	2	3 ↓ METHODE SUIVANTE	Avez-vous déjà eu une opération pour éviter d'avoir d'autres enfants?<br>OUI............................ 1<br>NON.......................... 2
02	STERILISATION MASCULINE/VASECTOMIE Les hommes peuvent subir une opération pour éviter d'avoir d'autres enfants.	1	2	3 ↓ METHODE SUIVANTE	Votre partenaire a t-il déjà eu une opération pour éviter d'avoir d'autres enfants ?<br>OUI............................ 1<br>NON.......................... 2
03	PILULE JOURNALIERE Les femmes peuvent prendre une pilule chaque jour pour éviter de tomber enceinte.	1	2	3 ↓ METHODE SUIVANTE	OUI............................ 1<br>NON.......................... 2
04	DIU Les femmes peuvent avoir un stérilet que le médecin, la sage-femme ou l'infirmier/ière leur place à l'intérieur de l'uterus.	1	2	3 ↓ METHODE SUIVANTE	OUI............................ 1<br>NON.......................... 2
05	INJECTABLES Les femmes peuvent avoir une injection faite par le personnel de santé pour éviter de tomber enceinte pendant un mois ou plus.	1	2	3 ↓ METHODE SUIVANTE	OUI............................ 1<br>NON.......................... 2
06	IMPLANTS Les femmes peuvent se faire placer sous la peau de la partie supérieure du bras un ou plusieurs petits bâtonnets qui vont les empêcher de tomber enceinte pendant une ou plusieurs années.	1	2	3 ↓ METHODE SUIVANTE	OUI............................ 1<br>NON.......................... 2
07	CONDOM MASCULIN Les hommes peuvent porter un préservatif sur leur pénis avant l'acte sexuel.	1	2	3 ↓ METHODE SUIVANTE	OUI............................ 1<br>NON.......................... 2
08	CONDOM FEMININ Les femmes peuvent placer un étui dans leur vagin avant l'acte sexuel.	1	2	3 ↓ METHODE SUIVANTE	OUI............................ 1<br>NON.......................... 2

09	LA METHODE DU RYTHME chaque mois, pendant qu'elle est sexuellement active, une femme peut éviter de tomber enceinte en n'ayant pas de rapports sexuels les jours du mois pendant lesquels elle court beaucoup de risques de tomber enceinte.	1 2 3 ↓ METHODE SUIVANTE	OUI............................ 1<br>NON.......................... 2
10	LA METHODE DU RETRAIT Les hommes peuvent être prudents et se retirer avant d'éjaculer.	1 2 3 ↓ METHODE SUIVANTE	OUI............................ 1<br>NON.......................... 2
11	CONTRACEPTION D'URGENCE Les femmes peuvent prendre des pilules jusqu'à 5 jours après un rapport sexuel pour éviter de tomber enceinte.	1 2 3 ↓ METHODE SUIVANTE	OUI............................ 1<br>NON.......................... 2
12	METHODE DE L'ALLAITEMENT MATERNEL ET DE L'AMENORRHEE (MAMA) jusqu'à 6 mois après l'accouchement, une femme peut utiliser une méthode qui exige qu'elle allaite fréquemment son bébé, le jour comme la nuit, et à condition qu'elle n'ait pas encore eu le retour de couche.	1 2 3 ↓ METHODE SUIVANTE	OUI............................ 1<br>NON.......................... 2
13	MOUSSE OU GEL Les femmes peuvent introduire un suppositoire, un gel ou de la crème dans leur vagin avant l'acte sexuel.	1 2 3 ↓ METHODE SUIVANTE	OUI............................ 1<br>NON.......................... 2
14	Avez-vous entendu parler d'autres moyens ou d'autres méthodes que les femmes ou les hommes peuvent utiliser pour éviter la grossesse ?<br>*NOTE: SI L'ENQUETEE MENTIONNE L'ABSTINENCE COMME UNE METHODE DE PF, NE PAS LE REPORTER COMME UNE METHODE.*<br>*INSISTER POUR TOUTE AUTRE METHODE CONNUE.*	OUI________________________ 1<br>(PRECISER)<br>NON... 3	OUI............................ 1<br>NON.......................... 2
15		OUI________________________ 1<br>(PRECISER)<br>NON... 3	OUI............................ 1<br>NON.......................... 2

No	QUESTIONS ET FILTRES	CODES	ALLER A
Q303	**VERIFIER Q301 :**<br>SI L'ENQUETEE A ENTENDU PARLER D'AU MOINS UNE METHODE (Q301=1 OU 2) ☐	SI LA REPONSE EST 'NON' (3) A TOUT ☐	Q401
Q304	Au cours de <u>12 derniers mois</u>, où avez-vous vu ou entendu parler de contraception ?<br><br>INSISTER: D'AUTRES SOURCES?<br><br>ENREGISTRER TOUT CE QUI EST MENTIONNE	**MEDIA**<br>RADIO…… AA<br>TELEVISION…… AB<br>JOURNAL…… AC<br>MAGAZINE…… AD<br>PANNEAUX D'AFFICHAGE…… AE<br>THEATRE SUR SCENE…… AF<br>ÉVENEMENTS COMMUNAUTAIRES…… AG<br>**SECTEUR PUBLIC**<br>HOPITAL GOUVERNEMENTAL…… BA<br>CENTRE SANTE GOUVERNEMENTAL…… BB<br>POSTE DE SANTE…… BC<br>STRATEGIE AVANCEE/EQUIPE MOBILE… BD<br>CENTRE CONSEILS ADOS…… BE<br>CASE DE SANTE…… BF<br>AUTRE PUBLIC…… BG<br>**SECTEUR PRIVE FORMEL**<br>HOPITAL/CLINIQUE/CABINET PRIVE…… CA<br>PHARMACIE…… CB<br>DISPENSAIRE RELIGIEUX…… CC<br>AUTRE MEDICAL PRIVE…… CD<br>**SECTEUR PRIVE INFORMEL**<br>MEDECIN…… DA<br>SAGE-FEMME…… DB<br>INFIRMIER/AI…… DC<br>MATRONE / ASC …… DD<br>GUERISSEUR ACCOUCHEUSE TRADITIONNELLE…… DE<br>**AUTRE SOURCE**<br>ÉCOLE…… EA<br>ÉGLISE/MOSQUEE…… EB<br>BAR…… EC<br>PARENTS/AMIS …… ED<br>ONG/OCB…… EF<br>**AUTRE**____________ XX<br>(PRECISER)<br>N'A NI VU, NI ENTENDU…… YY<br>NSP…… ZZ	
Q305	Avez-vous recommandé l'utilisation d'une <u>méthode de contraception</u> pour des raisons de planification familiale/ espacement des naissances à vos amis ou parents ?	OUI…… 1<br>NON…… 2	 Q307

Q306	Quelles sont les méthodes que vous avez recommandées ?<br><br>SI PILULE, INSISTER POUR SAVOIR SI PILULE JOURNALIERE OU CONTRACEPTION D'URGENCE.<br><br>ENCERCLER TOUT CE QUI EST MENTIONNE.	STERILISATION FEMININE .... A<br>STERILISATION MASCULINE............................ B<br>IMPLANT.. C<br>DIU .... D<br>INJECTABLES.. E<br>PILULE.. F<br>CONTRACEPTION D'URGENCE................... G<br>PRESERVATIF MASCULIN................................ H<br>PRESERVATIF FEMININ.................................... I<br>SPERMICIDE/MOUSSE/GEL............................. J<br>METHODES NATURELLES (METHODE DU RYTHME /ABSTINENCE PERIODIQUE /RETRAIT).. K<br>ALLAITEMENT AU SEIN /MAMA...................... L<br>AUTRE________________________________ X<br>(PRECISER)	
Q307	**VERIFIER Q302:**<br><br>A DEJA UTILISE AU MOINS UNE METHODE (AU MOINS UN "1" DANS Q302) : ☐ ↓	N'A JAMAIS UTILISE UNE METHODE (PARTOUT "2" A Q302) : ☐	→ Q347
Q308	Maintenant, je voudrais vous poser des questions sur la <u>première fois</u> que vous avez fait quelque chose ou utilisé une méthode pour éviter de tomber enceinte.<br><br>Combien d'enfants vivants aviez-vous en ce moment?<br>Combien de fils vivants aviez-vous en ce moment?<br>Combien de filles vivants aviez-vous en ce moment?<br><br>S'IL N'Y EN A AUCUN, ENREGISTRER '00'.	NOMBRE D'ENFANTS............. ☐☐<br>NOMBRE DE FILS.................... ☐☐<br>NOMBRE DE FILLES................ ☐☐	
Q309	**VERIFIER Q235 :**<br>PAS ACTUELLEMENT ENCEINTE (Q235=2 OU =8) : ☐ ↓	ACTUELLEMENT ENCEINTE (Q235=1) : ☐	→ Q337
Q310	Vous (ou votre partenaire) faites-vous ou utilisez-vous actuellement une méthode pour retarder ou éviter une grossesse ?	OUI... 1<br>NON.. 2<br>NE PEUT ETRE ENCEINTE........................ 3	 → Q337<br>→ Q337
Q311	Quelle(s) méthode(s) utilisez-vous actuellement?<br><br>SI PILULE, INSISTER POUR SAVOIR SI PILULE JOURNALIERE OU CONTRACEPTION D'URGENCE.<br><br>ENCERCLER TOUT CE QUI EST MENTIONNE.	STERILISATION FEMININE .... A<br>STERILISATION MASCULINE............................ B<br>IMPLANT.. C<br>DIU .... D<br>INJECTABLES.. E<br>PILULE.. F<br>CONTRACEPTION D'URGENCE................... G<br>PRESERVATIF MASCULIN................................ H<br>PRESERVATIF FEMININ.................................... I<br>SPERMICIDE/MOUSSE/GEL............................. J<br>METHODES NATURELLES (METHODE DU RYTHME /ABSTINENCE PERIODIQUE / RETRAIT).. K<br>ALLAITEMENT AU SEIN /MAMA...................... L<br>AUTRE________________________________ X<br>(PRECISER)	

Q312	**VERIFIER Q311 :**<br>SI PLUSIEURS METHODES MENTIONNEES, ENCERCLER DANS CETTE LISTE CELLE DONT LE CODE EST ARRIVE LE PREMIER PAR ORDRE ALPHABETIQUE ET CONTINUER AVEC CELLE-CI POUR LES QUESTIONS SUIVANTES.<br><br>SI UNE SEULE METHODE EST MENTIONNEE, ENCERCLER CETTE MÊME METHODE DANS CETTE LISTE.	STERILISATION FEMININE .... 01<br>STERILISATION MASCULINE............................. 02<br>IMPLANT.. 03<br>DIU .... 04<br>INJECTABLES.. 05<br>PILULE.. 06<br>CONTRACEPTION D'URGENCE........................ 07<br>PRESERVATIF MASCULIN................................ 08<br>PRESERVATIF FEMININ.................................... 09<br>SPERMICIDE/MOUSSE/GEL............................. 10<br>METHODES NATURELLES (METHODE<br>DU RYTHME /ABSTINENCE<br>PERIODIQUE / RETRAIT).......... 11<br>ALLAITEMENT AU SEIN /MAMA....................... 12<br>AUTRE________________________________ 96<br>(PRECISER)	
Q313	Pourquoi avez-vous choisie d'utiliser la (METHODE ENREGISTREE A Q312)?<br><br>INSISTER : Existe-t-il une autre raison ?<br><br>ENREGISTRER TOUT CE QUI EST MENTIONNE.	NE VEUT PAS TOMBER ENCEINTE.................. A<br>PAS D'EFFETS SECONDAIRES........................ B<br>NE VEUT PAS ÊTRE INFECTÉ PAR LE<br>VIH OU LES AUTRES ISTs............................ C<br>FACILE / PRATIQUE À UTILISER....................... D<br>METHODE DISCRETE....................................... E<br>ABORDABLE... F<br>FACILE A OBTENIR.. G<br>BEAUCOUP DE PERSONNES<br>L'UTILISENT... H<br>J'AIME COMME ON LE PREND AU<br>QUOTIDIEN... I<br>J'AIME COMME ON NE LE PREND PAS<br>AU QUOTIDIEN.................................... J<br>FAIT PERDRE DU POIDS.................................. K<br>FAIT PRENDRE DU POIDS................................. L<br>DONNE/FAIT MAINTENIR UN BON TEINT........ M<br>RECOMMANDÉ PAR UN PRESTATAIRE.......... N<br>MON PARTENAIRE LE PRÉFÈRE..................... O<br>JE N'AI PAS À M'OCCUPER DE ÇA; MON<br>PARTENAIRE EST RESPONSABLE<br>DU CHOIX... P<br>AUTRE ________________________________ X<br>(PRÉCISER)	
Q314	Qui décide de quelle méthode contraceptive utiliser ?<br><br>Est ce que c'est vous principalement, votre partenaire principalement, ou vous deux conjointement ?	VOUS PRINCIPALEMENT.............................. 1<br>PARTENAIRE PRINCIPALEMENT................. 2<br>CONJOINTEMENT.. 3<br>AUTRE______________________________ 6<br>(PRECISER)	
Q315	Un agent de santé ou un prestataire de planification familiale vous a t-il déjà parlé des effets indésirables ou des problèmes que vous pourriez avoir suite à l'utilisation de cette méthode de PF ((METHODE ENREGISTREE A Q312)?	OUI.. 1<br>NON... 2<br>NE SE RAPPELLE PAS.................................. 8	 → Q317<br>→ Q317
Q316	Vous a t-on dit ce qu'il faut faire en cas d'effets secondaires ou en cas de problèmes avec cette méthode ?	OUI.. 1<br>NON... 2<br>NE SE RAPPELLE PAS.................................. 8	

Q317	Un agent de santé ou un prestataire de planification familiale vous a-t-il déjà parlé d'autres méthodes de planification familiale que vous pourriez utiliser ?	OUI.. 1<br>NON.. 2<br>NE SE RAPPELLE PAS............................... 8	
Q318	En quel mois et en quelle année avez-vous commencé à utiliser [METHODE ACTUELLE] de manière continue?	MOIS.................................... [ ][ ]<br>NE CONNAIT PAS MOIS...................... 98<br><br>ANNEE..................... [ ][ ][ ][ ]<br>NE CONNAIT PAS L'ANNEE................ 9998	
Q319	**VERIFIER Q312:**<br>SI IMPLANT "03", DIU "04", INJECTABLES "05", PILULE "06", CONTRACEPTION D'URGENCE "07", PRESERVATIF MASCULIN "08",PRESERVATIF FEMININ "09", SPERMICIDE/MOUSSE/GEL "10", OU AUTRE PRODUIT "96" : [ ] ↓	SI STERILISATION FEMININE "01" : [ ]<br><br>SI STERILISATION MASCULINE "02", METHODES NATURELLES "11", MAMA "12", OU AUTRE METHODE NATURELLE "96" (POSTPARTUM ABSTINENCE, ETC.) : [ ]	→ Q330<br><br><br>→ Q334
Q320	Où/ de qui avez-vous obtenue (METHODE ACTUELLE) la dernière fois?<br><br>DEMANDER A CONNAITRE LE NOM ET L'EMPLACEMENT DE LA STRUCTURE/PERSONNE ET ENREGISTRER.	NOM ____________________________<br>ADRESSE ______________________<br>DONNER PLUS DE PRECISION<br>__________________________________	
Q321	Quel est le type de place/personne?<br><br>ENCERCLER LE TYPE DE STRUCTURE APPROPRIE. UNE SEULE REPONSE DOIT ETRE ENREGISTREE.	**SECTEUR PUBLIC**<br>HOPITAL GOUVERNEMENTAL.................. 11<br>CENTRE SANTE GOUVERNEMENTAL....... 12<br>POSTE DE SANTE.................................. 13<br>STRATEGIE AVANCEE/EQUIPE MOBILE.... 14<br>CENTRE CONSEILS ADOS....................... 15<br>CASE DE SANTE.................................... 16<br>AUTRE PUBLIC...................................... 17<br><br>**SECTEUR PRIVE FORMEL**<br>HOPITAL/CLINIQUE/CABINET PRIVE........ 21<br>PHARMACIE... 22<br>DISPENSAIRE RELIGIEUX...................... 23<br>AUTRE MEDICAL PRIVE......................... 24<br><br>**SECTEUR PRIVE INFORMEL**<br>MEDECIN.. 31<br>SAGE-FEMME....................................... 32<br>INFIRMIER/AI....................................... 33<br>MATRONE / ASC .... 34<br>GUERISSEUR /ACCOUCHEUSE<br>TRADITIONNELLE.......................... 35<br><br>**AUTRES SOURCES**<br>CLINIQUE AU LIEU DE TRAVAIL............... 41<br>CENTRE POUR JEUNE.......................... 42<br>MACHINE A VENDRE.............................. 43<br>CENTRE DE CONSEIL/TEST DE VIH......... 44<br>BAR.. 45<br>KIOSQUE/BOUTIQUE/MARCHE................ 46<br>ONG... 47<br>VOLONTAIRES/PAIRS ÉDUCATEURS....... 48<br>**AUTRE**______________________________ 96<br>(PRECISER)<br>NSP.. 98	

Q322	Vous/votre partenaire, avez-vous eu des difficultés à accéder à la [METHODE ACTUELLE] ?	OUI.. 1<br>NON... 2<br>NE SAIT PAS.. 8	 → Q324<br>→ Q324
Q323	Quelles sont les difficultés rencontrées pour accéder à la [METHODE ACTUELLE] ?<br><br>INSISTER : Avez-vous eu d'autres difficultés ?<br><br>ENREGISTRER TOUT CE QUI EST MENTIONNE.	CRAINTE QUE MON PARTENAIRE LE SACHE ; IL S'OPPOSE À L'UTILISATION....... A<br>CRAINTE QUE LES AUTRES PARENTS LE SACHENT ; ILS S'OPPOSENT À L'UTILISATION.. B<br>PAS EN MESURE DE QUITTER LA MAISON/LE TRAVAIL......................... C<br>PERSONNE POUR S'OCCUPER DES ENFANTS.. D<br>COÛT TRANSPORT TROP ÉLEVÉ.................... E<br>LE SERVICE EST SOUVENT FERMÉ................ F<br>COÛT SERVICE TROP ÉLEVÉ........................... G<br>TEMPS D'ATTENTE AU SERVICE TROP LONG... H<br>RUPTURES DE STOCK FRÉQUENTES AU SERVICE.. I<br>PRESTATAIRES SOUVENT ABSENTS............. J<br>PRESTATAIRES PAS ACCUEILLANTS............. K<br>METHODES EXPIREES.............................. L<br>MAUVAISES QUALITES DES SERVICES....... M<br>AUTRES ______________________________ X<br>(PRÉCISER)	
Q324	**VERIFIER Q312 : SI ENCERCLE**<br>PILULE (CODE="'06"): ☐ ↓	PRESERVATIF MASCULIN (CODE="'08") : ☐ →<br><br>IMPLANT "'03", DIU "'04", INJECTABLE "05", CONTRACEPTION D'URGENCE "'07", CONDOM FEMININ "'09", SPERMICIDE "10" OU AUTRE PRODUIT: "96" ☐ →	Q327<br><br>Q328
Q325	POUR LES UTILISATRICES DE PILLULES:<br><br>Quel est le nom de la marque des pilules que vous utilisez ?	PLANYL .... 01<br>PLANOR .... 02<br>OVRETTE.. 03<br>LO FEMENAL . ... 04<br>MINIDRIL... 05<br>MINIPHAS.. 06<br>STEDIRIL .... 07<br>MICROVA... 08<br>ADEPAL .... 09<br>MICROGYNON.. 10<br>NÉOGYNON .... 11<br>DIANE 35 .... 12<br>TRINORDIOL .... 13<br>SECURIL .... 14<br>AUTRE _______________________________ 96<br>(PRECISER)<br>NSP 98	

Q326	POUR CELLES QUI UTILISENT LES PILULES<br><br>Combien de pilules (plaquettes pour un cycle) avez-vous payées / obtenues la dernière fois ?	NBRE PLAQUETTES DE PILULES.. [ ][ ]<br>NE SAIT PAS…………………………………… 98	→ Q328
Q327	POUR LES FEMMES UTILISATRICES (AYANT DES PARTENAIRES UTILISATEURS) DE PRESERVATIFS:<br>Combien de préservatifs avez-vous payés/ obtenus la dernière fois?	NBRE DE PRESERVATIFS……… [ ][ ][ ]<br>NE SAIT PAS…………………………………… 998	
Q328	Quel est le prix que vous payez pour (METHODE ENREGISTREE A Q312)?	PRIX FCFA……….. ……. [ ][ ][ ][ ][ ]<br>GRATUIT……………………………………… 00000 → Q334<br>NE SAIT PAS……………………………………99998 → Q334	
Q329	Pensez-vous que ce prix est bas, normal ou cher?	PRIX BAS ………………………………………… 1<br>PRIX NORMAL …………………………………… 2<br>PRIX ELEVE ………………………………………3	→ Q334
Q330	POUR LES FEMMES QUI ONT ETE STERILISEES.<br><br>En quel mois et en quelle année la stérilisation avait t-elle été réalisée?<br><br>Combien d'enfants aviez-vous à ce moment-là?<br><br>Nombre de fils?<br><br>Nombre de filles ?	MOIS…….………………………………. [ ][ ]<br>NSP MOIS………..……………..…………..……. 98<br>ANNEE…………..………..…… [ ][ ][ ][ ]<br>NSP ANNEE………………………………………9998<br>NOMBRE D'ENFANTS……………….. [ ][ ]<br>FILS………….…………………………… [ ][ ]<br>FILLES……….……………………….. [ ][ ]	
Q331	Où avez-vous effectué l'opération de stérilisation?	**SECTEUR PUBLIC**<br>HOPITAL GOUVERNEMENTAL……………… 11<br>CENTRE SANTE GOUVERNEMENTAL……. 12<br>POSTE DE SANTE……………………………. 13<br>STRATEGIE AVANCEE/EQUIPE MOBILE…. 14<br>CENTRE CONSEILS ADOS…………………. 15<br>CASE DE SANTE………………………………16<br>AUTRE PUBLIC……………………………… 17<br><br>**SECTEUR PRIVE FORMEL**<br>HOPITAL/CLINIQUE/CABINET PRIVE…….. 21<br>PHARMACIE…………………………………. 22<br>DISPENSAIRE RELIGIEUX………………… 23<br>AUTRE MEDICAL PRIVE…………………… 24<br><br>**SECTEUR PRIVE INFORMEL**<br>MEDECIN……………………………………… 31<br>SAGE-FEMME…………………………………32<br>INFIRMIER/AI………………………………… 33<br>MATRONE / ASC .... 34<br>GUERISSEUR /ACCOUCHEUSE<br>TRADITIONNELLE…………….. 35<br><br>**ONG/OCB…………………………………………** 47<br><br>**AUTRE**______________________________ 96<br>(PRECISER)<br>NSP……………………………………………… 98	

Q332	Quel est le prix que vous avez payé pour l'opération de stérilisation ?	PRIX FCFA… [ ][ ][ ][ ][ ][ ][ ]<br>GRATUIT……………………………………0000000<br>NE SAIT PAS………………………………9999998	 →Q334<br>→Q334
Q333	Pensez-vous que ce prix est bas, normal ou cher?	PRIX BAS ………………………………………… 1<br>PRIX NORMAL ………………………………… 2<br>PRIX ELEVE ……………………………………… 3	
Q334	Avant d'utiliser [METHODE ACTUELLE], aviez-vous (ou votre partenaire) utilisé une autre méthode différente ?	OUI ……………………………………………… 1<br>NON ……………………………………………… 2	 → Q354
Q335	Quelle méthode aviez-vous utilisé avant la [METHODE ACTUELLE] ?<br><br>SI PILULE, INSISTER POUR SAVOIR SI PILULE JOURNALIERE OU CONTRACEPTION D'URGENCE.<br><br>UNE SEULE REPONSE DOIT ETRE ENREGISTREE.	STERILISATION MASCULINE............................ 02<br>IMPLANT... 03<br>DIU ………………………………………………...... 04<br>INJECTABLES……………………………………... 05<br>PILULE...……………………………………….…... 06<br>CONTRACEPTION D'URGENCE……………….... 07<br>PRESERVATIF MASCULIN................................ 08<br>PRESERVATIF FEMININ.................................... 09<br>SPERMICIDE/MOUSSE/GEL............................. 10<br>METHODES NATURELLES (METHODE<br>DU RYTHME /ABSTINENCE<br>PERIODIQUE/ RETRAIT)……………………… 11<br>ALLAITEMENT AU SEIN /MAMA……………….. 12<br>AUTRE______________________________ 96<br>(PRECISER)	02–96 → Q354
Q337	A présent, je voudrais vous parler de la dernière fois que vous avez utilisé une méthode ou fait quelque chose pour éviter de tomber enceinte.<br>Combien d'enfants vivants aviez-vous lorsque vous aviez commencé à utiliser / faire, si toutefois vous en aviez ?<br><br>Combien de fils ?<br>Combien de filles ?	NOMBRE D'ENFANTS……………….. [ ][ ]<br><br>FILS………….…………………………… [ ][ ]<br><br>FILLES………...……………………….. [ ][ ]	
Q338	Quelle(s) méthodes aviez-vous utilisé (la dernière fois que vous avez utilisé une méthode)?<br><br>SI PILULE, INSISTER POUR SAVOIR SI PILULE JOURNALIERE OU CONTRACEPTION D'URGENCE.<br><br>ENREGISTRER TOUT CE QUI EST MENTIONNE.	STERILISATION MASCULINE............................ B<br>IMPLANT... C<br>DIU ………………………………………………...... D<br>INJECTABLES……………………………………... E<br>PILULE...……………………………………….…... F<br>CONTRACEPTION D'URGENCE……………….... G<br>PRESERVATIF MASCULIN................................ H<br>PRESERVATIF FEMININ.................................... I<br>SPERMICIDE/MOUSSE/GEL............................. J<br>METHODES NATURELLES (METHODE<br>DU RYTHME /ABSTINENCE<br>PERIODIQUE / RETRAIT)………………… K<br>ALLAITEMENT AU SEIN /MAMA……………….. L<br>AUTRE______________________________ X<br>(PRECISER)	

Q339	**VERIFIER Q338 :**<br>SI PLUSIEURS METHODES MENTIONNEES, ENCERCLER <u>DANS CETTE LISTE CELLE DONT LE CODE EST ARRIVE LE PREMIER PAR ORDRE ALPHABETIQUE</u> ET CONTINUER AVEC CELLE-CI POUR LES QUESTIONS SUIVANTES.<br><br>SI UNE SEULE METHODE EST MENTIONNEE, ENCERCLER CETTE MÊME METHODE DANS CETTE LISTE.	STERILISATION MASCULINE............................ 02<br>IMPLANT.. 03<br>DIU .... 04<br>INJECTABLES.. 05<br>PILULE... 06<br>CONTRACEPTION D'URGENCE.................... 07<br>PRESERVATIF MASCULIN................................ 08<br>PRESERVATIF FEMININ.................................... 09<br>SPERMICIDE/MOUSSE/GEL............................ 10<br>METHODES NATURELLES (METHODE DU RYTHME /ABSTINENCE PERIODIQUE / RETRAIT).......... 11<br>ALLAITEMENT AU SEIN /MAMA...................... 12<br>AUTRE_______________________________ 96<br>(PRECISER)	
Q340	Pourquoi avez-vous arrêté d'utiliser cette méthode [ENCERCLEE A Q339]?<br><br>INSISTER: d'autres raisons?<br><br>ENCERCLER TOUT CE QUI EST MENTIONNE.	SOUHAITAIT TOMBER ENCEINTE................ A<br>ECHEC DE METHODE/ EST TOMBEE ENCEINTE B<br>ABSENCE DE DESIR SEXUEL...................... C<br>PROVOQUE DES PROBLEMES DE CYCLE.... D<br>PROVOQUE DES PROBLEMES DE SANTE.... E<br>RAPPORTS SEXUELS NON FREQUENTS...... F<br>N'EST PAS PRATIQUE A UTILISER............... G<br>DIFFICILE A OBTENIR................................. H<br>FAIT GAGNER DU POIDS............................. I<br>FAIT PERDRE DU POIDS............................. J<br>COUTE TROP CHERE................................. K<br>N'A PAS AIME LA METHODE........................ L<br>MANQUE D'INTIMITE.................................. M<br>PARTENAIRE/ MARI N'APPROUVE PAS .... N<br>MÉNOPAUSE/HYSTÉRECTOMIE .... O<br>PRESTATAIRE A DEMANDE D'ARRETER...... P<br>PEUR DE DEVENIR STERILE....................... Q<br>PEUR DES EFFETS INDESIRABLES .... R<br>AUTRE_______________________________ X<br>(PRECISER)	
Q341	Avez-vous déjà eu des problèmes avec cette méthode (ENCERCLEE A Q339) ?	OUI.. 1<br>NON.. 2	 → Q347
Q342	Quels problèmes avez-vous eu avec cette méthode ?<br><br>INSISTER: d'autres problèmes?<br><br>ENCERCLER TOUT CE QUI EST MENTIONNE.	METHODE A ECHOUE/ TOMBEE ENCEINTE.. A<br>INSATISFACTION SEXUELLE......................... B<br>A PROVOQUE DES PROBLEMES DE CYCLE. C<br>MAL DE DOS... D<br>MAUX DE TËTE.. E<br>NAUSE/VOMISSEMENT................................ F<br>A PROVOQUE DES PROBLEMES DE SANTE. G<br>DIFFICILE A UTILISER............................ ..... H<br>DIFFICILE A OBTENIR.................................. I<br>FAIT GAGNER DU POIDS............................. J<br>FAIT PERDRE DU POIDS............................. K<br>METHODE T'AFFAIBLIE................................ L<br>COUTE TROP CHERE................................. M<br>N'AIMAIT PAS LA METHODE .... N<br>MANQUE D'INTIMITE.................................. O<br>PARTENAIRE/ MARI N'APPROUVE PAS........ P<br>AUTRE_______________________________ X<br>(PRECISER)	

Q343	Avez-vous parlé à quelqu'un de vos problèmes?	OUI…… 1<br>NON…… 2	 → Q345
Q344	A qui avez-vous parlé?<br><br>INSISTER: quelqu'un d'autres?<br><br>ENCERCLER TOUT CE QUI EST MENTIONNE.	**PERSONNEL MEDICAL**<br>MEDECIN …… A<br>SAGE-FEMME…… B<br>INFIRMIER…… C<br>MATRONNE…… D<br>PHARMACIEN…… E<br>**PERSONNEL COMMUNAUTAIRE**<br>ACCOUCHEUSE TRADITIONNELLE…… F<br>GUERISSEUR TRADITIONNEL…… G<br>AGENT DE SANTE COMMUNAUTAIRE.. H<br>PAIR-EDUCATEUR…… I<br>**FAMILLE/PROCHES**<br>AMI(E)S…… J<br>MERE…… K<br>BELLE-MERE…… L<br>SOEUR…… M<br>BELLE SOEUR…… N<br>PARTENAIRES/EPOUX…… O<br>AUTRE PROCHE…… P<br>**AUTRE**________________ X<br>(PRECISER)	
Q345	Avez-vous demandé de l'aide auprès d'un personnel de santé pour résoudre les problèmes?	OUI…… 1<br>NON…… 2	 → Q347
Q346	Où êtes-vous allée demander de l'aide?<br><br>INSISTER: Un autre endroit ou une autre personne?<br><br>ENCERCLER TOUT CE QUI EST MENTIONNE.	**SECTEUR PUBLIC**<br>HOPITAL GOUVERNEMENTAL…… A<br>CENTRE SANTE GOUVERNEMENTAL…… B<br>POSTE DE SANTE…… C<br>STRATEGIE AVANCEE/EQUIPE MOBILE…. D<br>CENTRE CONSEILS ADOS…… E<br>CASE DE SANTE…… F<br>AUTRE PUBLIC…… G<br><br>**SECTEUR PRIVE FORMEL**<br>HOPITAL/CLINIQUE/CABINET PRIVE…… H<br>PHARMACIE…… I<br>DISPENSAIRE RELIGIEUX…… J<br>AUTRE MEDICAL PRIVE…… K<br><br>**SECTEUR PRIVE INFORMEL**<br>MEDECIN…… L<br>SAGE-FEMME…… M<br>INFIRMIER/AI…… N<br>MATRONE / ASC …… O<br>GUERISSEUR /ACCOUCHEUSE<br>TRADITIONNELLE…… P<br><br>**AUTRE**<br>ONG/OCB…… Q<br>VOLONTAIRES/PAIRS ÉDUCATEURS…… R<br><br>**AUTRE**________________ X<br>(PRECISER)	

Q347	Quelle sont les principales raisons pour lesquelles vous n'utilisez pas/plus une méthode de planification familiale pour retarder ou éviter une grossesse?<br><br>INSISTER: Autres raisons?<br><br>ENCERCLER TOUT CE QUI EST MENTIONNE.	**RAISONS DE FECONDITE**<br>RAPPORTS SEXUELS NON FREQUENTS / PAS DE RAPPORTS SEXUELS………… A<br>PARTENAIRE/MARI ABSENT………………. B<br>MENOPAUSE/HYSTERECTOMIE………… C<br>DEJA ENCEINTE……………………………. D<br>ALLAITEMENT……………………………… E<br>NE PEUT PAS AVOIR D'ENFANTS……….. F<br>SOUHAITE AVOIR AUTANT D'ENFANTS QUE POSSIBLE…………………………… G<br>SOUHAITE TOMBER/ESSAIE DE TOMBER ENCEINTE…………………………… H<br>AMENORRHEE POSTPARTUM……………. I<br>**OPPOSITION A L'UTILISATION**<br>L'ENQUETEE EST OPPOSEE……………. J<br>LE PARTENAIRE EST OPPOSE………….. K<br>D'AUTRES PERSONNES SONT OPPOSEES………………………………. L<br>INTERDICTION RELIGIEUSE………………. M<br>**MANQUE DE CONNAISSANCE** :<br>NE SAIT PAS COMMENT UTILISER UNE METHODE…………………………… N<br>NE CONNAIT AUCUNE SOURCE………… O<br>**RAISONS LIEES A LA METHODE**<br>PROBLEMES DE SANTE……………………. P<br>PEUR DES EFFETS SECONDAIRES……… Q<br>MANQUE D'ACCES / TROP ELOIGNE…….. R<br>COUTE TROP CHERE………………………. S<br>PAS PRATIQUE A UTILISER………………. T<br>N'AIME PAS LES METHODES EXISTANT… U<br>EXPERIENCES MALHEUREUSES AVEC LES METHODES EXISTANT……………. V<br>FATALISTE: DEPEND DE DIEU …………… W<br><br>**AUTRE**_______________________________ X<br>(PRECISER)<br>NE SAIT PAS ……………………………………… Z	 <br><br>→Q354 (C)<br><br><br>→Q354 (F)
Q348	Pensez-vous que vous ou votre partenaire utiliserez une méthode pour retarder ou éviter une grossesse au cours des 12 prochains mois ?	OUI……………………………………………… 1<br>NON……………………………………………… 2<br>NE SAIT PAS……………………………………. 8	 →Q354<br>→Q354

Q349	Quelle méthode préfèreriez-vous utiliser LE PLUS, si vous utilisez effectivement une méthode à l'avenir?<br><br>SI PILULE, INSISTER POUR SAVOIR SI PILULE JOURNALIERE OU CONTRACEPTION D'URGENCE.	STERILISATION FEMININE .... 01<br>STERILISATION MASCULINE............................ 02<br>IMPLANT.. 03<br>DIU .... 04<br>INJECTABLES... 05<br>PILULE... 06<br>CONTRACEPTION D'URGENCE.......................... 07<br>PRESERVATIF MASCULIN................................ 08<br>PRESERVATIF FEMININIO................................ 09<br>SPERMICIDE/MOUSSE/GEL............................. 10<br>METHODES NATURELLES (METHODE DU RYTHME /ABSTINENCE PERIODIQUE/ RETRAIT)...................... 11<br>ALLAITEMENT AU SEIN /MAMA.......................... 12<br>AUTRE_______________________________ 96<br>(PRECISER)	 <br><br><br><br><br><br><br><br><br><br><br>→ Q354<br>→ Q354
Q350	Connaissez-vous un endroit ou une personne où vous pouvez obtenir cette méthode (METHODE ENREGISTRER A Q349)?	OUI... 1<br>NON.. 2	 → Q352
Q351	Quel est cet endroit / cette personne ?	**SECTEUR PUBLIC**<br>HOPITAL GOUVERNEMENTAL.................. 11<br>CENTRE SANTE GOUVERNEMENTAL....... 12<br>POSTE DE SANTE.................................. 13<br>STRATEGIE AVANCEE/EQUIPE MOBILE.... 14<br>CENTRE CONSEILS ADOS....................... 15<br>CASE DE SANTE.................................... 16<br>AUTRE PUBLIC..................................... 17<br><br>**SECTEUR PRIVE FORMEL**<br>HOPITAL/CLINIQUE/CABINET PRIVE........ 21<br>PHARMACIE.. 22<br>DISPENSAIRE RELIGIEUX...................... 23<br>AUTRE MEDICAL PRIVE......................... 24<br><br>**SECTEUR PRIVE INFORMEL**<br>MEDECIN... 31<br>SAGE-FEMME....................................... 32<br>INFIRMIER/AI....................................... 33<br>MATRONE / ASC .... 34<br>GUERISSEUR /ACCOUCHEUSE TRADITIONNELLE......................... 35<br><br>**AUTRE**<br>CLINIQUE AU LIEU DE TRAVAIL............... 41<br>CENTRE DE CONSEIL/TEST DE VIH......... 44<br>ONG/OCB... 47<br>VOLONTAIRES/PAIRS ÉDUCATEURS....... 48<br><br>**AUTRE**_______________________________ 96<br>(PRECISER)<br>NSP.. 98	
Q352	Seriez-vous prête à payer pour obtenir cette méthode (METHODE ENREGISTRER A Q349)?	OUI... 1<br>NON.. 2<br>NE SAIT PAS.. 8	 → Q354<br>→ Q354
Q353	Si OUI, combien seriez-vous prête à payer pour obtenir cette méthode (METHODE ENREGISTRER A Q349)?	MONTANT (FCFA). [ ][ ][ ][ ][ ][ ]	

Q354	Maintenant, je voudrais vous poser quelques questions sur ce que vous pensez des méthodes hormonales, comme les pilules et injectables.<br><br>Quels sont les effets secondaires les plus fréquents que vous connaissez pour ces méthodes ?<br><br>INSISTER : Existe-t-il un autre effet?<br><br>ENCERCLER TOUT CE QUI EST MENTIONNE.	SAIGNEMENT OU PROBLÈMES DE MENSTRUE A<br>GAIN DE POIDS.......... B<br>PERTE DE POIDS.......... C<br>MAUX DE TÊTES / CÉPHALÉES.......... D<br>MAUX DE DOS.......... E<br>NAUSÉES / VOMISSEMENTS.......... F<br>INSOMNIE.......... G<br>FATIGUE / FAIBLESSE PHYSIQUE.......... H<br>AUTRE PROBLÈMES DE SANTÉ.......... I<br>INFERTILITE.......... J<br>CANCER.......... K<br>MALFORMATION DES ENFANTS.......... L<br>PERTE DU DÉSIR SEXUEL.......... M<br>AUTRE_______________ X<br>(PRÉCISER)<br>NE CONNAIT PAS DE METHODES HORMONALE Y<br>NE CONNAIT AUCUN EFFET SECONDAIRE OU INCONVÉNIENT.......... Z	
Q372	Maintenant, je voudrais vous poser quelques questions relatives à ce que vous connaissez et pensez des préservatifs.<br><br>Si on utilise correctement un préservatif masculin, pensez-vous qu'il protège contre une grossesse la plupart du temps, seulement souvent,ou pas du tout ?	LA PLUPART DU TEMPS.......... 1<br>SOUVENT.......... 2<br>PAS DU TOUT.......... 3<br>N'A JAMAIS ENTENDU PARLER DE PRESERVATIFS.......... 4	4 → Q376
Q373	Pensez-vous que le fait d'utiliser un préservatif réduit le plaisir sexuel de la femme ?	OUI.......... 1<br>NON.......... 2<br>NE SAIT PAS.......... 8	
Q374	Pensez-vous que le fait d'utiliser un préservatif réduit le plaisir sexuel de l'homme ?	OUI.......... 1<br>NON.......... 2<br>NE SAIT PAS.......... 8	
Q375	Pensez-vous que le fait d'utiliser un préservatif est un signe d'infidélité ?	OUI.......... 1<br>NON.......... 2<br>NE SAIT PAS.......... 8	

	QUALITE DES SOINS – SERVICES ET TRAITEMENT					
	S'il vous plait, pouvez vous me dire si vous êtes totalement d'accord, d'accord, pas d'accord ou totalement contre les arguments suivants:	TOTALEMENT D'ACCORD	D'ACCORD	PAS D'ACCORD	TOTALEMENT CONTRE	
Q376	Les prestataires de services de PF d'ici ne traitent pas bien les clients	4	3	2	1	
Q377	Les femmes n'aiment pas la manière dont elles sont traitées dans les cliniques de PF d'ici	4	3	2	1	
Q378	Les prestataires/vendeurs de produits PF se comportent de manière à ce que que les femmes se sentent mal à l'aise lorsqu'elles viennent se procurer des contraceptifs	4	3	2	1	

	CROYANCES-REPRESENTATIONS					
	S'il vous plait, pouvez vous me dire si vous êtes totalement d'accord, d'accord, pas d'accord ou totalement contre les arguments suivants:	TOTALEMENT D'ACCORD	D'ACCORD	PAS D'ACCORD	TOTALEMENT CONTRE	
Q379	Se faire injecter un produit contraceptif rend la femme stérile pour toujours	4	3	2	1	
Q380	Les personnes qui utilisent des contraceptifs finissent par avoir des problèmes de santé	4	3	2	1	
Q381	Les contraceptifs peuvent faire du mal à l'utérus	4	3	2	1	
Q382	Les contraceptifs réduisent le désir sexuel	4	3	2	1	
Q383	Les contraceptifs peuvent causer le cancer	4	3	2	1	
Q384	Les contraceptifs peuvent vous donner des bébés malformés	4	3	2	1	
Q385	Les contraceptifs sont dangereux pour la santé	4	3	2	1	

SECTION 4: SANTE MATERNELLE ET INFANTILE

Maintenant, je voudrais vous poser des questions relatives à la naissance de votre dernier enfant.

Q401	**VERIFIER Q230:**<br>UNE NAISSANCE OU PLUS EN 2009 OU PLUS TARD : ☐ ↓<br>AUCUNE NAISSANCE EN 2009 OU PLUS TARD : ☐ →		**Q408**
Q402	REPORTER LE NOM ET LE NUMERO DE LIGNE DU DERNIER ENFANT NÉ DEPUIS 2009 ET MENTIONNÉS EN Q218 :<br>_______________<br>NOM	☐☐<br>N° LIGNE	
Q403	A présent, je voudrais vous parler de la naissance de [NOM DU DERNIER ENFANT].<br>Qui vous a assistée lors de la naissance de [NOM DU DERNIER ENFANT]?<br>INSISTER: Quelqu'un d'autres?<br>ENCERCLER TOUT CE QUI EST MENTIONNE.	**PROFESSIONNEL DE SANTÉ**<br>MÉDECIN………… A<br>SAGE-FEMME………… B<br>INFIRMIÈRE/ICP………… C<br>AUTRE PROFESSIONNEL DE SANTE________ D<br>(PRECISER)<br>**AUTRE PERSONNE**<br>MATRONE………… E<br>ACCOUCHEUSE TRADITIONNELLE………… F<br>AMIE………… G<br>PARTENAIRES/ÉPOUX………… H<br>MÈRE………… I<br>BELLE MÈRE………… J<br>SŒUR………… K<br>BELLE SŒUR………… L<br>**AUTRE**________________ X<br>(PRECISER)<br>**PERSONNE…………** Y	
Q404	L'endroit où vous avez accouché votre dernier enfant [NOM DU DERNIER ENFANT], est-ce dans cette ville (le site), une autre ville ou en zone rurale ?	CETTE VILLE (SITE)………… 1<br>UNE AUTRE VILLE………… 2<br>ZONE RURALE………… 3<br>ETRANGER………… 4	
Q405	Où avez-vous accouché (NOM DU DERNIER ENFANT)?<br>_______________<br>(NOM DE LA STRUCTURE)	**SECTEUR PUBLIC**<br>HÔPITAL GOUVERNEMENTAL………… 11<br>CENTRE SANTÉ GOUVERNEMENTAL………… 12<br>POSTE DE SANTÉ………… 13<br>STRATÉGIE AVANCÉE/ÉQUIPE MOBILE………… 14<br>CENTRE CONSEILS ADOS………… 15<br>CASE DE SANTE………… 16<br>AUTRE PUBLIC………… 17<br>**SECTEUR PRIVÉ FORMEL**<br>HÔPITAL/CLINIQUE/CABINET PRIVÉ………… 21<br>PHARMACIE………… 22<br>DISPENSAIRE RELIGIEUX………… 23<br>AUTRE MÉDICAL PRIVÉL………… 24<br>**SECTEUR PRIVÉ INFORMEL**<br>MÉDECIN………… 31<br>SAGE-FEMME………… 32<br>INFIRMIER/AI………… 33<br>MATRONE / ASC ………… 34<br>GUÉRISSEUR/ACCOUCHEUSE TRADITIONNELLE. 35<br>**A DOMICILE**<br>CHEZ MOI………… 51<br>CHEZ PARENTS/AMIS ………… 52<br>CHEZ PARTENAIRE………… 53<br>**AUTRE**________________ 96<br>(PRECISER)	(11–24) **Q407**

Q406	Pourquoi n'avez-vous pas accouché dans une structure de santé?<br><br>INSISTER: D'autres raisons?<br><br>ENCERCLER TOUT CE QUI EST MENTIONNE.	COUTE TROP CHER…… A<br>STRUCTURE DE SANTE NON OUVERTE…… B<br>N'A PAS EU LE TEMPS…… C<br>NE FAIT PAS CONFIANCE AU PERSONNEL…… D<br>PAS NECESSAIRE…… E<br>NE FAIT PAS PARTIE DE NOS COUTUMES…… F<br>TROP ELOIGNE…… G<br>PAS DE MOYEN DE TRANSPORT DISPONIBLE …… H<br>PERSONNE N'ETAIT DISPONIBLE POUR L'ACCOMPAGNER…… I<br>MAUVAISE QUALITE DES SERVICES…… J<br>N'OFFRE PAS LES SERVICES DONT ON A BESOIN…. K<br>PRESTATAIRES SOUVENT ABSENTS…… L<br>N'ACCEPTE PAS D'ASSURANCE…… M<br>PAS DE PRESTATAIRE FEMME…… N<br>PARTENAIRE/FAMILLE N'ACCEPTERONT PAS…… O<br>AUTRE______ X<br>(PRECISER)	→ **SQ15**
Q407	Un agent de santé ou un prestataire de santé vous a-t-il fourni des informations relatives aux méthodes de planification familiale que ce soit avant ou après votre accouchement ?	AVANT L'ACCOUCHEMENT…… 1<br>APRES L'ACCOUCHEMENT…… 2<br>AVANT ET APRES L'ACCOUCHEMENT…… 3<br>N'A REÇU AUCUNE INFORMATION…… 4<br>NE SE RAPPELLE PAS…… 8	
	Maintenant, je voudrais vous poser des questions relatives à l'état de santé de votre dernier né, spécifiquement si il ou elle a reçu des vaccinations.		
SQ15	**VERIFIER 402 POUR LE NOM DU DERNIER ENFANT**<br><br>Quelles vaccinations votre dernier enfant (NOM DU DERNIER ENFANT) a t-il reçues?<br><br>INSISTER: D'autres vaccinations?<br><br>ENCERCLER TOUT CE QUI EST MENTIONNE.<br><br>DEMANDER A CONSULTER LE CARNET DE VACCINATION DE L'ENFANT	BCG…… A<br>PENTAVALENT 1 (CONTIENT POLIO)…… B<br>PENTAVALENT 2…… C<br>PENTAVALENT 3…… D<br>ROUGEOLE…… E<br>FIÈVRE JAUNE…… F<br>AUTRE______ X<br>(PRECISER)<br>AUCUNE…… Y<br>NE SAIT PAS…… Z	Y → **Q408**
SQ16	(NOM) a t-il déjà reçu le vaccin contre la polio, c'est-à-dire des gouttes dans la bouche, y compris le vaccin qui est donné lors d'une campagne 'Bouttez la Polio' ?	OUI…… 1<br>NON…… 2<br>NE SAIT PAS…… 8	2 → **SQ19**<br>8 → **SQ19**
SQ17	Le <u>premier</u> vaccin contre la polio a t-il été reçu au cours des <u>deux premières semaines</u> après la naissance ou plus tard?	2 PREMIERES SEMAINES…… 1<br>PLUS TARD…… 2<br>NE SAIT PAS…… 8	
SQ18	Combien de fois a t-il reçu le vaccin contre la polio?<br><br>SI PLUS DE 7 FOIS, ENREGISTRER '7'	NOMBRE DE FOIS…… [ ]	
SQ19	Ou votre dernier enfant a-t-il recu la majorite de ses vaccins?<br><br>DEMANDER LE NOM ET L'ADRESSE DE CE LIEU<br>DEMANDER LES DETAILS.	______<br>(NOM DE LA STRUCTURE)<br>______<br>ADRESSE (NUMÉRO, NOM DE LA RUE, VILLE)<br>______<br>DESCRIPTION (PRES D'OÙ?)	

SQ20	Quel est le type de place?	**SECTEUR PUBLIC**<br>HÔPITAL GOUVERNEMENTAL…………..…………. 11<br>CENTRE SANTÉ GOUVERNEMENTAL…..…………. 12<br>POSTE DE SANTÉ……………………..……………..…… 13<br>STRATÉGIE AVANCÉE/ÉQUIPE MOBILE…………… 14<br>CENTRE CONSEILS ADOS………………...…………. 15<br>CASE DE SANTE…………………………………………. 16<br>AUTRE PUBLIC…………………………………………… 17<br>**SECTEUR PRIVÉ FORMEL**<br>HÔPITAL/CLINIQUE/CABINET PRIVÉ……...………. 21<br>PHARMACIE………………...……………..……….…… 22<br>DISPENSAIRE RELIGIEUX………………...…………. 23<br>AUTRE MÉDICAL PRIVÉ………………...……………. 24<br>**SECTEUR PRIVÉ INFORMEL**<br>MÉDECIN……………………...……………..…….……. 31<br>SAGE-FEMME……………………...…………….……… 32<br>INFIRMIER/AI………………..………………..………… 33<br>MATRONE / ASC ....………….....……………..…… 34<br>GUÉRISSEUR/ACCOUCHEUSE TRADITIONNELLE. 35<br><br>**AUTRE**______________________________ 96<br>(PRECISER)<br>NSP……………...……………..………...………………….. 98	
SQ21	Au cours des <u>visites pour la vaccination</u>, avez-vous reçu des conseils ou informations sur la PF / l'espacement des naissances ?	OUI………………...……………………………………. 1<br>NON………………...……………………………………. 2<br>NE SAIT PAS………………...……………………………. 8	 → Q408<br>→ Q408
SQ22	Au cours des <u>visites pour la vaccination</u>, avez-vous reçu une méthode ou une consultation pour la PF / l'espacement des naissances/ ?	OUI, J'Ai RECU UNE METHODE…………...……………... 1<br>OUI, J'AI RECU UNE ORDANNANCE…………..………… 2<br>OUI, J'AI RECU UNE REFERENCE…………..………… 3<br>N'A PAS RECU……………..………………..……………. 4<br>NE SE SOUVIENT PAS …………...……………..………... 8	
Q408	**VERIFIER Q218-Q222 (HISTORIQUE DES NAISSANCES):**<br>OUI, A UN OU PLUSIEURS ENFANTS EN VIE : ☐ ↓	NON, N'A AUCUNE NAISSANCE EN VIE: ☐	→ Q416
	Maintenant, je vais vous poser des questions relatives à l'expérience que vous avez eue suite à la demande ou à la réception de conseils médicaux.		
Q409	Au cours des <u>3 derniers mois</u>, êtes vous allé dans un service de santé pour des <u>soins de l'un de vos enfants</u> (ENFANTS DE MOINS DE 10 ANS)?	OUI………………...……………………………………. 1<br>NON………………...……………………………………. 2	 → Q416
Q410	Quels types de services avez vous reçu durant ces dernières visites?<br><br>INSISTER: D'autres services?<br><br>ENREGISTRER TOUT CE QUI EST MENTIONNÉ.	VACCINATION……………………………...……………. A<br>PREVENTION DES MALADIES…………………..………. B<br>SOINS DE L'ENFANT (POUR DIARRHEE,<br>PALUDISME, INFECTION RESPIRATOIRE)……….. C<br>CROISSANCE DE L'ENFANT………………………………. D<br>CONSULTATION ? (BILAN DE SANTE). …………………. E<br>AUTRE_________________________ X<br>(PRECISER)	
Q411	Ou avez vous visité le <u>plus fréquemment</u> pour les <u>soins de l'un de vos enfants</u> ?<br><br>DEMANDER LE NOM ET L'ADRESSE DE CE LIEU<br>DEMANDER LES DETAILS.	____________________________<br>(NOM DE LA STRUCTURE)<br>____________________________<br>ADRESSE (NUMÉRO, NOM DE LA RUE, VILLE)<br>____________________________<br>DESCRIPTION (PRES D'OÙ?)	

Q412	Quel type de service est cette structure ou vous avez visité le plus fréquemment pour les soins de l'enfant ?<br><br>ENCERCLER LE TYPE APPROPRIE DE STRUCTURE DE SANTE.	**SECTEUR PUBLIC**<br>HÔPITAL GOUVERNEMENTAL……………..………… 11<br>CENTRE SANTÉ GOUVERNEMENTAL…..…………… 12<br>POSTE DE SANTÉ…………………....…………….…… 13<br>STRATÉGIE AVANCÉE/ÉQUIPE MOBILE…………… 14<br>CENTRE CONSEILS ADOS…………………..………… 15<br>CASE DE SANTE………………………………………… 16<br>AUTRE PUBLIC………………………………………… 17<br>**SECTEUR PRIVÉ FORMEL**<br>HÔPITAL/CLINIQUE/CABINET PRIVÉ……………….... 21<br>PHARMACIE……………………………....…………….. 22<br>DISPENSAIRE RELIGIEUX………………...…………… 23<br>AUTRE MÉDICAL PRIVÉ…………………….…………. 24<br>**SECTEUR PRIVÉ INFORMEL**<br>MÉDECIN………………....……………..……………….. 31<br>SAGE-FEMME……………....……………..…………….. 32<br>INFIRMIER/AI…………………....………………..………. 33<br>MATRONE / ASC ....………….…....………………. 34<br>GUÉRISSEUR/ACCOUCHEUSE TRADITIONNELLE. 35<br>**AUTRE**_______________________________ 96<br>(PRECISER)<br>NSP………………...……………...……………………….. 98	
Q413	Pourquoi avez-vous choisi cette structure?<br><br>INSITER : D'autres raisons?<br><br>ENREGISTRER TOUT CE QUI EST MENTIONNE.	SERVICES GRATUITS / ABORDABLES…………………. A<br>SERVICE OUVERT / HORAIRES D'OUVERTURES CONVENABLES…………………………………………. B<br>LE PERSONNEL EST POLI ET RESPECTABLE………… C<br>CONVENABLE PAR RAPPORT À MON DOMICILE…….. D<br>CONVENABLE PAR RAPPORT À MON LIEU DE TRAVAIL……………………………………………..... E<br>CONVENABLE PAR RAPPORT A L'ENDROIT OÙ J'EFFECTUE MES ACHATS……………….............. F<br>CONVENABLE PAR RAPPORT LA DISPONIBILITÉ DES TRANSPORTS……………………......…….. G<br>BONNE QUALITÉ DES SERVICES………………………. H<br>OFFRE LES SERVICES REQUIS…………………………. I<br>PRESTATAIRES DISPONIBLES…………………………. J<br>ACCEPTE LES ASSURANCES……………………………. K<br>OFFRE DES FACILITÉS DE CRÉDIT……………………. L<br>LE SERVICE A UNE BONNE RÉPUTATION……………. M<br>IL OFFRE PLUSIEURS TYPES DE SERVICES…………. N<br>J'AI ÉTÉ RÉFÉRÉE LÀ-BAS………………………………. O<br>AUTRE_______________________________ X<br>(PRECISER)	
Q414	Au cours de cette visite, avez vous reçu des informations ou conseils sur la PF ?	OUI………………………………………………………… 1<br>NON………………………………………………………. 2	 → **Q416**
Q415	Au cours de cette visite, avez vous reçu une méthode ou une consultation sur la PF ?	OUI, J'Ai RECU UNE METHODE…………………………. 1<br>OUI, J'AI RECU UNE ORDANNANCE……………………. 2<br>OUI, J'AI RECU UNE REFERENCE……………………... 3<br>N'A PAS RECU…………………………………………… 4<br>NE SE SOUVIENT PAS ……………………………………8	
Q416	Au cours des 12 derniers mois, êtes-vous allée dans un service de santé pour des soins maternelles (ci incluse les soins postnatales, les soins prénatales)?	OUI …………………………………………………… 1<br>NON ………………………………………………….. 2	 → **Q422**
Q417	Où avez vous visité le plus fréquemment pour les soins de santé maternels?<br><br>DEMANDER LE NOM ET L'ADRESSE DE CE LIEU	______________________<br>(NOM DE LA STRUCTURE)<br>______________________<br>ADRESSE (NUMÉRO, NOM DE LA RUE, VILLE)<br>______________________<br>DESCRIPTION (PRES D'OÙ?)	

Q418	Quel type de service est cette structure que vous avez visitée le <u>plus fréquemment</u> pour les <u>soins maternels</u> ?<br><br>ENCERCLER LE TYPE APPROPRIE DE STRUCTURE DE SANTE.	**SECTEUR PUBLIC**<br>HÔPITAL GOUVERNEMENTAL………………..……… 11<br>CENTRE SANTÉ GOUVERNEMENTAL…...………… 12<br>POSTE DE SANTÉ……….……......…...….....………….... 13<br>STRATÉGIE AVANCÉE/ÉQUIPE MOBILE……...…… 14<br>CENTRE CONSEILS ADOS……………..…...………… 15<br>CASE DE SANTE………………………………………. 16<br>AUTRE PUBLIC…………………………………………. 17<br>**SECTEUR PRIVÉ FORMEL**<br>HÔPITAL/CLINIQUE/CABINET PRIVÉ……....……….. 21<br>PHARMACIE………………..……………..……………. 22<br>DISPENSAIRE RELIGIEUX……….….………………. 23<br>AUTRE MÉDICAL PRIVÉ……………...……………......... 24<br>**SECTEUR PRIVÉ INFORMEL**<br>MÉDECIN……………......……....…………....………..... 31<br>SAGE-FEMME…………………………………………..... 32<br>INFIRMIER/AI………………………………...……........... 33<br>MATRONE / ASC .... 34<br>GUÉRISSEUR/ACCOUCHEUSE TRADITIONNELLE. 35<br>**AUTRE**________________________________ 96<br>(PRECISER)<br>NSP……………………………………..……………. 98	
Q419	Pourquoi avez-vous choisi cette structure?<br><br>INSITER : D'autres raisons?<br><br>ENCERCLER TOUT CE QUI EST MENTIONNE.	SERVICES GRATUITS / ABORDABLES………………….. A<br>SERVICE OUVERT / HORAIRES D'OUVERTURES<br>CONVENABLES………………………………………… B<br>LE PERSONNEL EST POLI ET RESPECTABLE………… C<br>CONVENABLE PAR RAPPORT À MON DOMICILE…….. D<br>CONVENABLE PAR RAPPORT À MON LIEU<br>DE TRAVAIL…………………………………………..... E<br>CONVENABLE PAR RAPPORT L'ENDROIT OÙ<br>J'EFFECTUE MES ACHATS……………....……….. F<br>CONVENABLE PAR RAPPORT A LA DISPONIBILITÉ<br>DES TRANSPORTS…………………….....………… G<br>BONNE QUALITÉ DES SERVICES……………………… H<br>OFFRE LES SERVICES REQUIS………………………… I<br>PRESTATAIRES DISPONIBLES…………………………… J<br>ACCEPTE LES ASSURANCES…………………………… K<br>OFFRE DES FACILITÉS DE CRÉDIT……………………… L<br>LE SERVICE A UNE BONNE RÉPUTATION……………. M<br>IL OFFRE PLUSIEURS TYPES DE SERVICES…………. N<br>J'AI ÉTÉ RÉFÉRÉE LÀ-BAS………………………………… O<br>AUTRE________________________________ X<br>(PRECISER)	
Q420	Au cours de cette visite, avez vous reçu des informations ou conseils sur la PF ?	OUI . ………………………………………………………… 1<br>NON …………………………………………………………. 2	 → **Q422**
Q421	Aviez-vous reçu une méthode ou une consultation sur la PF pendant cette visite?	OUI, J'Ai RECU UNE METHODE…………………………… 1<br>OUI, J'AI RECU UNE ORDONNANCE……………………… 2<br>OUI, J'AI RECU UNE REFERENCE . . . . ………………… 3<br>N'A PAS RECU…………………..……………………… 4<br>NE SE SOUVIENT PAS…………………………………….. 8	

Q422	Au cours de 12 derniers mois, êtes vous allé dans un service pour le Dépistage Volontaire du VIH ?	OUI .... 1<br>NON 2	 → Q427
Q423	Où avez vous visité le plus fréquemment pour le Dépistage Volontaire du VIH ?<br><br>DEMANDER LE NOM ET L'ADRESSE DE CE LIEU.<br><br>DEMANDER LES DETAILS.	____________________<br>(NOM DE LA STRUCTURE)<br>____________________<br>ADRESSE (NUMÉRO, NOM DE LA RUE, VILLE)<br>____________________<br>DESCRIPTION (PRES D'OÙ?)	
Q424	Quel type de service est cette structure que vous avez visitéé le plus fréquemment pour le Dépistage Volontaire du VIH ?<br><br>ENCERCLER LE TYPE APPROPRIE DE STRUCTURE DE SANTE.	**SECTEUR PUBLIC**<br>HÔPITAL GOUVERNEMENTAL.......................... 11<br>CENTRE SANTÉ GOUVERNEMENTAL.................. 12<br>POSTE DE SANTÉ.. 13<br>STRATÉGIE AVANCÉE/ÉQUIPE MOBILE.............. 14<br>CENTRE CONSEILS ADOS................................ 15<br>CASE DE SANTE.. 16<br>AUTRE PUBLIC.. 17<br>**SECTEUR PRIVÉ FORMEL**<br>HÔPITAL/CLINIQUE/CABINET PRIVÉ.................. 21<br>PHARMACIE.. 22<br>DISPENSAIRE RELIGIEUX................................ 23<br>AUTRE MÉDICAL PRIVÉ.................................... 24<br>**SECTEUR PRIVÉ INFORMEL**<br>MÉDECIN.. 31<br>SAGE-FEMME.. 32<br>INFIRMIER/AI.. 33<br>MATRONE / ASC .... 34<br>GUÉRISSEUR/ACCOUCHEUSE TRADITIONNELLE. 35<br><br>**CENTRE DE CONSEIL/TEST DE VIH.........................** 44<br>**AUTRE**____________________ 96<br>(PRECISER)<br>NSP.. 98	
Q425	Au cours de cette visite, avez vous reçu des informations ou conseils sur la PF ?	OUI.. 1<br>NON.. 2	 → Q427
Q426	Aviez-vous reçu une méthode ou une consultation sur la PF pendant cette visite?	OUI, J'AI RECU UN PRESERVATIF............................ 1<br>OUI, J'AI RECU UNE AUTRE METHODE DE PF........... 2<br>OUI, J'AI RECU UNE ORDONNANCE......................... 3<br>OUI, J'AI RECU UNE REFERENCE .... 4<br>N'A PAS RECU.. 5<br>NE SE SOUVIENT PAS.. 8	
Q427	Quel est le nom de la pharmacie où vous (ou les membres de ce ménage) allez souvent pour acheter des médicaments ?<br><br>Où est-ce qu'elle est située ?	____________________<br>(NOM DE LA STRUCTURE)<br>____________________<br>ADRESSE (NUMÉRO, NOM DE LA RUE, VILLE)<br>____________________<br>DESCRIPTION (PRES D'OÙ?)	
Q428	Avez-vous dejà reçu de l'information ou conseil au sujet de la PF ou de la contraception au monent de l'achat des medicaments dans cette pharmacie?	OUI.. . ... 1<br>NON.. 2	

SECTION 5: MARIAGE ET ACTIVITES SEXUELLES

No	QUESTIONS ET FILTRES	CODES	ALLER A
	A présent, je voudrais vous poser quelques questions sur votre activité sexuelle. Permettez-moi de vous rappeler encore que vos réponses sont entièrement confidentielles et personne n'en sera informé. Si nous arrivons à une question à laquelle vous ne voudrez pas répondre, vous avez juste à me le faire savoir et nous sauterons pour la question suivante.		
Q501	Maintenant, je dois vous poser des questions sur votre <u>activité sexuelle</u> afin de mieux comprendre certaines questions se rapportant à la vie de famille.<br><br>Quel âge aviez-vous lorsque vous avez eu un rapport sexuel pour la toute <u>première fois</u>?	AGE…………………………………………………. …. [ ][ ]<br>N'A JAMAIS EU DE RAPPORTS SEXUELS…….. **00**	 → Q511
Q502	La <u>première fois</u> que vous avez eu un rapport sexuel, aviez-vous (ou votre partenaire) utilisé une méthode de PF ?	OUI ……………………………………………………… 1<br>NON……………………………………………………… 2<br>NE SE SOUVIENT PAS………………………………… 8	 → Q504<br>→ Q504
Q503	Quelle méthode aviez vous utilisé ?<br><br>ENREGISTRER TOUT CE QUI EST MENTIONNE.<br><br>SI PILULE, INSISTER POUR SAVOIR SI PILULE JOURNALIERE OU CONTRACEPTION D'URGENCE.	STERELISATION FEMININE ……………………………… A<br>STERELISATION MASCULINE……………………… B<br>IMPLANT……………………………………………… C<br>DIU ……………………………………………………… D<br>INJECTABLES…………………………………………… E<br>PILULE…………………………………………………… F<br>CONTRACEPTION D'URGENCE………………………… G<br>PRESERVATIF MASCULIN…………………………… H<br>PRESERVATIF FEMININ……………………………… I<br>SPERMICIDE/MOUSSE/GEL………………………… J<br>METHODE DU RYTHME /ABSTINENCE<br>PERIODIQUE/ RETRAIT……………………………… K<br>AUTRE_______________________________ X<br>(PRECISER)	
Q504	A présent, je voudrais vous poser quelques questions sur votre activité sexuelle récente. Permettez-moi de vous rappeler encore que vos réponses sont entièrement confidentielles et personne n'en sera informé. Si nous arrivons à une question à laquelle vous ne voudrez pas répondre, vous avez juste à me le faire savoir et nous sauterons pour la question suivante.		
Q505	Quand avez-vous eu vos <u>derniers rapports sexuels</u>?<br><br>SI MOINS DE 12 MOIS, LA REPONSE DOIT ETRE ENREGISTREE EN JOURS, SEMAINES OU MOIS.<br>SI 12 MOIS (UNE ANNEE) OU PLUS, LA REPONSE DOIT ETRE ENREGISTREE EN ANNEES.<br>SI MOINS D'UNE JOURNEE, ENREGISTRER « 00 ».	IL Y A DES JOURS……………………………… 1 [ ][ ]<br>IL Y A DES.SEMAINES……………………………… 2 [ ][ ]<br>IL Y A DES MOIS……………………………… 3 [ ][ ]<br>IL Y A DES ANNEES……………………………… 4 [ ][ ]	 <br><br>→ Q511
Q506	Combien de fois avez vous eu des rapports sexuels dans les <u>trois derniers mois</u> ?<br><br>SI MOINS DE 100 FOIS, METTRE '0' A LA PREMIERE CASE.	NOMBRE DE FOIS……………………………… [ ][ ][ ]<br>OU<br>N'A PAS EU DES RAPPORTS SEXUELS…………… 000<br>OU<br>TOUS LES JOURS……………………………………… 991<br>OU<br>1 FOIS TOUTES LES SEMAINES ………………… 992<br>OU<br>1 FOIS TOUS LES MOIS………………………… 993<br>OU<br>AUTRE_______________________________ 996<br>(PRECISER)<br>NE SAIT PAS……………………………………… 998	
Q509	<u>La dernière fois</u> que vous avez eu un rapport sexuel, aviez-vous (ou votre partenaire) utilisé une méthode de PF ?	OUI ………………………………………………… 1<br>NON……………………………………………………… 2<br>NE SE SOUVIENT PAS……………………………… 8	 → Q511<br>→ Q511

Q510	Quelle méthode aviez-vous utilisé ?<br><br>SI PILULE, INSISTER POUR SAVOIR SI PILULE JOURNALIERE OU CONTRACEPTION D'URGENCE.<br><br>ENCERCLER TOUT CE QUI EST MENTIONNE.<br><br>CORRIGER Q302 ET Q303 (ET Q301 SI NÉCESSAIRE).	STERILISATION FEMININE .... A<br>STERILISATION MASCULINE.......... B<br>IMPLANT.......... C<br>DIU .... D<br>INJECTABLES.......... E<br>PILULE.......... F<br>CONTRACEPTION D'URGENCE.......... G<br>PRESERVATIF MASCULIN.......... H<br>PRESERVATIF FEMININ.......... I<br>SPERMICIDE/MOUSSE/GEL.......... J<br>METHODES NATURELLES (METHODE DU RYTHME /ABSTINENCE PERIODIQUE / RETRAIT).......... K<br>ALLAITEMENT AU SEIN /MAMA.......... L<br>AUTRE______________________ X<br>(PRECISER)	
	Maintenant, je voudrais vous poser des questions sur votre situation matrimoniale.<br>Rappelez-vous que vos réponses resteront confidentielles.		
Q511	Avez-vous jamais été marié ou vécu maritalement avec un homme ?	OUI, A ÉTÉ MARIEE.......... 1<br>OUI, A VECU AVEC UN HOMME.......... 2<br>NON.......... 3	 <br>→Q601
Q512	Quel est votre état matrimonial actuel : êtes-vous mariée ou vivez-vous avec un homme, veuve, divorcée, ou séparée?	ACTUELLEMENT MARIEE.......... 1<br>VIT AVEC UN HOMME.......... 2<br>VEUVE.......... 3<br>DIVORCEE.......... 4<br>SEPAREE.......... 5	 <br>}<br>→ Q517
Q513	Votre mari/partenaire vit-il actuellement avec vous, où habite t-il ailleurs?	VIT AVEC ELLE.......... 1<br>HABITE AILLEURS.......... 2	
Q514	A part vous, est-ce que votre mari/partenaire a d'autres épouses ou vit-il avec d'autres femmes comme s'il était marié ?	OUI.......... 1<br>NON .... 2<br>NE SAIT PAS.......... 8	 →Q517<br>→Q517
Q515	Y compris vous , avec combien de femmes ou de partenaires au total votre mari/partenaire vit-il actuellement maritalement ?	NOMBRE TOTAL DE FEMMES ET DE PARTENAIRES AVEC QUI IL VIT MARITALEMENT.......... [ ][ ]<br>NE SAIT PAS.......... 98	
Q516	Etes-vous la première, seconde,……femme/ partenaire ?	RANG .... [ ][ ]	
Q517	Avez-vous été mariée une seule fois ou plus d'une fois/ avez-vous vécu avec un seul homme ou avec plus d'un homme?	SEULEMENT UNE FOIS.......... 1<br>PLUS D'UNE FOIS.......... 2	
Q518	**VERIFIER Q517 :**<br><br>A ÉTÉ MARIEE UNE SEULE FOIS/A VECU AVEC UN SEUL HOMME<br>En quel mois et en quelle année avez-vous commencé a vivre avec votre mari / partenaire ?<br><br>A ÉTÉ MARIEE PLUS D'UNE FOIS/A VECU AVEC PLUS D'UN HOMME<br>Maintenant, je voudrais savoir quand est-ce que vous avez commencé à vivre avec votre premier mari / partenaire. En quel mois et en quelle année était-ce ?	N'A PAS COMMENCE A VIVRE AVEC SON MARI...... 96<br><br>MOIS.......... [ ][ ]<br>NE CONNAIT PAS LE MOIS.......... 98<br><br>ANNEE.......... [ ][ ][ ][ ]<br>NE CONNAIT PAS L'ANNEE.......... 9998	**→Q601**
Q519	Quel âge aviez-vous lorsque vous avez commencé au début à vivre avec lui ?	AGE.......... [ ][ ]<br>NE SAIT PAS.......... 98	

SECTION 6: PREFERENCES EN MATIERE DE FECONDITE

Q601	**VERIFIER *Q311* :**<br>AUCUN DES DEUX N'EST STERILISE : ☐ (NI CODE A, NI CODE B ENCERCLÉ) ↓<br>IL OU ELLE EST STERILISE (E): ☐ (CODE A OU CODE B ENCERCLÉ) → Q607		Q607
Q602	**VERIFIER Q235:**<br>PAS ENCEINTE OU PAS SURE ☐ (Q235=2 OU 8) ↓<br>Maintenant, j'ai des questions concernant l'avenir. Voudriez-vous avoir Un(autre) enfant ou préfèreriez-vous ne pas avoir d(autres) enfants?<br>ENCEINTE (Q235=1) : ☐ ↓<br>Maintenant, j'ai des questions concernant l'avenir. Après l'enfant que vous attendez actuellement, aimeriez-vous avoir un autre enfant, ou d'autres enfants ou préfèreriez-vous ne pas avoir du tout ?	AVOIR (UN / UN AUTRE) ENFANT..................... 1<br>PAS D'AUTRES /AUCUN................................ 2<br>NE PEUT PAS TOMBER ENCEINTE .... 3<br>NE SAIT PAS OU INDECISE............................ 8	 → Q604<br>→ Q607<br>→ Q604
Q603	**VERIFIER Q602:**<br>NON ENCEINTE OU PAS SURE : ☐ (Q235=2 OU 8) ↓<br>Combien de temps voudriez-vous attendre à partir de maintenant avant la naissance d'un (autre) enfant ?<br>ENCEINTE : (Q235=1) ☐ ↓<br>Après la naissance de l'enfant que vous attendez actuellement, combien de temps voudriez-vous attendre avant la naissance d'un autre enfant ?<br>SI LA REPONSE EST UN NOMBRE DECIMAL DE MOIS OU D'ANNEES, CONVERTIR LE TOUT EN MOIS ET REPORTER DANS LA CASE « MOIS ».	MOIS.. 1 ☐☐<br>ANNEES... 2 ☐☐<br>BIENTOT/MAINTENANT.................................. 993<br>APRES LE MARIAGE...................................... 995<br>AUTRE________________________________ 996<br>(PRECISER)<br>NE SAIT PAS... 998	
Q604	**VERIFIER Q603 :**<br>NON ENCEINTE OU PAS SURE: ☐ ↓<br>ENCEINTE : ☐ → Q607		Q607
Q605	Dans les semaines qui viennent, si vous découvriez que vous êtes enceinte, cela serait-il un problème important, un petit problème ou cela ne vous poserait-il aucun problème?	GROS PROBLEME.. 1<br>PETIT PROBLEME.. 2<br>PAS UN PROBLEME....................................... 3<br>PAS DE RAPPORT SEXUEL............................ 4	 <br>→ Q607<br>→ Q607
Q606	Pourquoi est ce que ce serait un problème ?<br>INSISTER: D'autres raisons?<br>ENREGISTRER TOUT CE QUI EST MENTIONNE.	STATUT ECONOMIQUE/TRAVAIL... ....A<br>BEAUCOUP/ASSEZ D'ENFANT........................ B<br>SANTE DE LA MERE...................................... C<br>PAS D'EPOUX/ PARTENAIRE.......................... D<br>A DEJA UN PETIT ENFANT............................. E<br>RAISONS RELIGIEUSES................................. F<br>RAISONS LIEES A L'ECOLE............................ G<br>LE PARTENAIRE NE VEUT PAS....................... H<br>LA FAMILLE DESAPPROUVE.......................... I<br>NE VEUT PAS D'ENFANT................................ J<br>AUTRE________________________________ X<br>(PRECISER)	

Q607	**VERIFIER Q511 ET Q512:**<br>MARIEE OU VIT ACTUELLEMENT AVEC UN HOMME (CODE 1 OU 2 DANS Q512): ☐ ↓<br>PAS EN UNION : ☐ → (Q511=3 OU Q512=3, 4 OU 5)		Q612
Q608	**VERIFIER Q604:**<br>*Parlons maintenant de votre partenaire et de ses préférences* pour l'avenir.<br>NON ENCEINTE OU PAS SURE : ☐ ↓<br>Aimerait-il avoir un (autre) enfant, ou préférerait-il ne pas avoir d'(autres) enfants?<br>ENCEINTE : ☐ ↓<br>Après l'enfant que vous attendez actuellement, votre partenaire aimerait-il avoir un autre enfant, ou préférerait-il ne plus avoir d'autres enfants?	AVOIR (UN / UN AUTRE) ENFANT…………………1<br>PAS PLUS / AUCUN ………………………………….2<br>NE PEUT PAS TOMBER ENCEINTE……………….3<br>NE CONNAIT PAS LE DESIR DE SON PARTENAIRE……………………………….8	 → Q610<br>→ Q610<br>→ Q611
Q609	**VERIFIER Q608:**<br>NON ENCEINTE OU PAS SURE : ☐ ↓<br>Combien de temps aimerait-il attendre à partir de maintenant Jusqu'à la naissance d'un (autre) enfant ?<br>ENCEINTE : ☐ ↓<br>Après la naissance de l'enfant que vous attendez actuellement, combien de temps aimerait-il attendre avant la naissance d'un autre enfant ?	MOIS……………………………………… 1 ☐☐<br>ANNEES…………………………………2 ☐☐<br>BIENTOT/MAINTENANT……………………………. 993<br>NE PEUT PAS TOMBER ENCEINTE………………. 994<br>APRES LE MARIAGE……………………………… 995<br>AUTRE________________________________996<br>(PRECISER)<br>NE SAIT PAS…………………………………………. 998	
Q610	Votre mari/partenaire veut-il le même nombre d'enfants que vous, ou veut-il plus ou moins que vous ?	MEME NOMBRE……………………………………… 1<br>PLUS D'ENFANTS……………………………………2<br>MOINS D'ENFANTS…………………………………3<br>NE SAIT PAS…………………………………………8	
Q611	Qui décide du nombre d'enfants que vous allez avoir -diriez-vous que cette décision vous appartient principalement, appartient principalement à votre mari/partenaire, ou avez-vous décidé tous les deux ensemble ?	PRINCIPALEMENT VOUS…………………………… 1<br>PRINCIPALEMENT VOTRE PARTENAIRE……….2<br>CONJOINTEMENT……………………………………3<br>AUTRE________________________________ 6<br>(PRECISER)	
Q612	**VERIFIER LES NAISSANCES Q218 ET Q222: ENFANTS EN VIE?**<br>Maintenant, parlons encore de vous et de vos préférences dans le domaine de la maternité .<br>A DES ENFANTS EN VIE ☐ ↓<br>Si vous pouviez revenir à l'époque où vous n'aviez pas d'enfants et pouviez choisir exactement le nombre d'enfants à avoir dans toute votre vie, combien auriez-vous voulu en avoir ?<br>PAS D'ENFANTS EN VIE : ☐ ↓<br>Si vous pouviez choisir exactement le nombre d'enfants à avoir dans toute votre vie, combien voudriez-vous en avoir ?<br>INSISTER POUR AVOIR UNE REPONSE NUMERIQUE.	AUCUN……………………………………………… 00<br>NOMBRE…………………………………… ☐☐<br>AUTRE________________________________ 96<br>(PRECISER)	→ Q614<br><br>→ Q614
Q613	Parmi ces enfants, combien souhaiteriez-vous de garçons, combien souhaiteriez-vous de filles, et pour combien d'entre eux, le sexe n'aurait-il pas d'importance ?	NOMBRE GARCONS………………….. ☐☐<br>NOMBRE FILLES……………………….. ☐☐<br>L'UN OU L'AUTRE……………………. ☐☐	

Q614	Selon vous, combien de temps un couple doit il attendre après le mariage pour avoir un enfant?	IMMEDIATEMENT…………………………………… 1<br>MOINS D'UN AN…………………………………… 2<br>ENTRE UN AN ET AVANT 2 ANS APRES<br>MARIAGE……………………………………… 3<br>2 OU PLUSIEURS ANNEES…………………………… 4<br>AUTRE________________________________ 6<br>(PRECISER)	
Q615	Selon vous, quel doit être l'âge idéal (années) pour une femme d'avoir son premier enfant(de tomber enceinte)?	AGE EN ANNEES……………………… … [ ][ ]<br>NE SAIT PAS…………………………………… 98	
Q616	Selon vous, quel est l'intervalle idéal entre deux enfants ?<br><br>SI LA REPONSE EST UN NOMBRE DECIMAL DE MOIS OU D'ANNEES,CONVERTIR LE TOUT EN MOIS ET REPORTER DANS LA CASE « MOIS ».	MOIS………………………………… **1** [ ][ ]<br>ANNEES……………………………… **2** [ ][ ]<br>NE SAIT PAS…………………………………… 998	
Q617	Quels sont les effets (positifs et/ ou négatif) <u>sur la mère</u> si elle utilise une methode de PF?<br><br>INSISTER: D'autres effets?<br><br>ENREGISTRER TOUT CE QUI EST MENTIONNE.	MEILLEUR ETAT NUTRITIONNEL………………… A<br>INCIDENCE D'ANEMIE PLUS FAIBLE……………… B<br>MOINS DE COMPLICATIONS DE GROSSESSE… C<br>PREVENIR IST /VIH………………………………… D<br>PREVENIR GROSSESSES INDESIRABLES……… E<br>MOINS D'ENFANT A EDUQUER…………………… F<br>LA FEMME A PLUS DE TEMPS LIBRE…………… G<br>LA FAMILLE GAGNE PLUS D'ARGENT…………… H<br>ESPRIT TRANQUILLE……………………………… I<br>BONHEUR FAMILIAL /ENTENTE DANS<br>LE COUPLE………………………………… J<br>MEILLEUR SANTE DE LA MERE…………………… K<br>PROBLEMES DE COUPLE/DISPUTES/DIVORCES… L<br>REJET COMMUNAUTE/PROCHES………………… M<br>PROBLEMES DE CYCLE…………………………… N<br>PROBLEMES DE SANTE…………………………… O<br>GAIN DE POIDS………………………………………… P<br>PERTE DE POIDS…………………………………… Q<br>AFFAIBLISSEMENT…………………………………… R<br>AUTRE________________________________ X<br>(PRECISER)<br>AUCUN………………………………………………… Y<br>NSP……………………………………………………… Z	
Q618	Quels sont les effets (positifs et/ ou négatif) <u>sur l'enfant</u> si la mère iutilise une methode de PF?<br><br>INSISTER: D'autres effets?<br><br>NE PAS LIRE LES REPONSES.	MEILLEURE CROISSANCE…………………………… A<br>MEILLEUR ETAT NUTRITIONNEL………………… B<br>MEILLEUR SANTE……………………………………… C<br>MEILLEURE CHANCE DE SURVIE………………… D<br>PLUS GRANDE ATTENTION DE LA MERE……… E<br>MEILLEURE EDUCATION…………………………… F<br>PLUS D'OPPORTUNITES DANS LA VIE………… G<br>PLUS FAIBLE INCIDENCE DES MALADIES……… H<br>MALFORMATIONS……………………………………… I<br>PROBLEMES DE CROISSANCE…………………… J<br>OBESITE………………………………………………… K<br>PROBLEMES NUTRITIONNELS…………………… L<br>MALADIES FREQUENTES…………………………… M<br>NAISSANCES MULTIPLES/JUMEAUX……………… N<br>AUTRE________________________________ X<br>(PRECISER)<br>AUCUN………………………………………………… Y<br>NSP……………………………………………………… Z	

Q619	**VERIFIER Q511 ET Q512:**<br><br>MARIEE OU VIT AVEC UN HOMME ACTUELLEMENT (Q512=1 OU 2) ☐ ↓<br><br>N'EST PAS EN UNION ☐ (Q511=3 OU Q512=3,4 OU 5)		→ Q701
Q620	Votre partenaire vous a-t-il déjà accompagné (ou a-t-il accompagné votre enfant) dans une structure sanitaire ?	OUI .... 1<br>NON 2	 → Q701
Q621	Quelle est la fréquence avec laquelle il vous accompagne (ou votre enfant) ?	RAREMENT........ 1<br>QUELQUEFOIS........ 2<br>SOUVENT........ 3<br>TOUJOURS........ 4	

SECTION 7: COMMUNICATION ENTRE EPOUX ET INTERPERSONNELLE

Q701	Je voudrais vous demander quelques questions sur les discussions que vous auriez eues avec d'autres personnes au sujet des problemes de santé.<br>**VERIFIER Q511 ET Q512:**<br>MARIEE OU VIT ACTUELLEMENT AVEC UN HOMME : ☐ (Q512=1 OU 2) ↓<br>PAS EN UNION : ☐ (Q511=3 OU Q512=3, 4 OU 5)		→ Q709
Q702	Avez-vous, vous et votre époux/partenaire, déjà discuté du nombre d'enfants que vous aimeriez avoir ?	OUI ..... 1<br>NON 2	 → Q704
Q703	Quelle est la fréquence avec laquelle vous avez parlé à votre époux/partenaire de ce sujet au cours des 6 derniers mois?	N'AVONS PAS DISCUTE AU COURS DES SIX DERNIERS MOIS..... 1<br>UNE OU DEUX FOIS..... 2<br>PLUS DE DEUX FOIS..... 3<br>SOUVENT/REGULIEREMENT..... 4<br>AUTRE_____ 6<br>(PRECISER)	
Q704	Avez-vous déjà discuté de PF/l'utilisation de la contraception avec votre époux/partenaire ?	OUI ..... 1<br>NON 2	 → Q708
Q705	Quelle est la fréquence avec laquelle vous avez parlé à votre époux/partenaire de ce sujet au cours des 6 derniers mois?	N'AVONS PAS DISCUTE AU COURS DES SIX DERNIERS MOIS..... 1<br>UNE OU DEUX FOIS..... 2<br>PLUS DE DEUX FOIS..... 3<br>SOUVENT/REGULIEREMENT..... 4<br>AUTRE_____ 6<br>(PRECISER)	
Q706	D'habitude, qui d'entre-vous commence la discussion sur la PF, vous ou votre époux/partenaire ?	MOI-MÊME..... 1<br>EPOUX/PARTENAIRE..... 2<br>L'UN OU L'AUTRE..... 3	
Q707	Quel est le degré de difficulté à commencer une conversation sur l'espacement des naissances avec votre partenaire – très difficile, quelque peu difficile, facile ?	TRES DIFFICILE..... 1<br>QUELQUE PEU DIFFICILE..... 2<br>FACILE..... 3	
Q708	Avez-vous l'intention de parler à votre époux de la contraception au cours des 3 prochains mois ?	OUI ..... 1<br>NON ..... 2<br>NE SAIT PAS..... 8	
Q709	En plus de vous-même (ou de votre époux/partenaire) qui d'autre influence la décision concernant l'utilisation ou non de la contraception ?<br><br>INSISTER: Une autre personne?<br><br>ENREGISTRER TOUT CE QUI EST MENTIONNE.	PERE..... A<br>MERE..... B<br>BEAU-PERE..... C<br>BELLE-MERE..... D<br>SOEUR(S)..... E<br>BELLE(S)-SOEUR(S)..... F<br>AMIES..... G<br>AGENT COMMUNAUTAIRE..... H<br>RESPONSABLE COMMUNAUTAIRE..... I<br>GRAND-MERE..... J<br>TANTE..... K<br>LEADERS RELIGIEUX..... L<br>AUTRES PARENTS..... M<br>AUTRE_____ X<br>(PRECISER)<br>AUCUN..... Y	

Q710	Avez-vous besoin du consentement de votre partenaire ou des membres de votre famille pour utiliser une méthode de planification familiale ?	OUI.......... 1<br>NON. .... 2<br>NE SAIT PAS.......... 8	 → Q712<br>→ Q712
Q711	Quelles sont les personnes dont vous avez besoin d'obtenir le consentement pour utiliser une méthode de PF ?<br><br>INSISTER: Quelqu'un d'autres?<br><br>ENREGISTRER TOUT CE QUI EST MENTIONNE.	EPOUX/PARTENAIRE.......... A<br>MERE.......... B<br>BELLE-MERE.......... C<br>PERE.......... D<br>BEAU-PERE.......... E<br>SŒURS.......... F<br>BELLES-SŒURS.......... G<br>AUTRES PARENTS.......... H<br>AUTRE ____________ X<br>(PRECISER)	
Q712	Pensez-vous que les autorités gouvernementales devraient parler publiquement de PF/Contraception ?	OUI.......... 1<br>NON.......... 2<br>NE SAIT PAS.......... 8	
Q713	Au cours de 12 derniers mois, avez-vous entendu ou vu un responsable gouvernemental parler publiquement contre la planification familiale ?	OUI .... 1<br>NON .... 2<br>NE SE RAPPELLE PAS.......... 8	
Q714	Au cours de 12 derniers mois, avez-vous entendu ou vu un responsable gouvernemental parler publiquement en faveur de la planification familiale?	OUI .... 1<br>NON .... 2<br>NE SE RAPPELLE PAS.......... 8	
Q715	Pensez-vous que les leaders religieux devraient parler publiquement de PF/Contraception ?	OUI.......... 1<br>NON.......... 2<br>NE SAIT PAS.......... 8	
Q716	Au cours de 12 derniers mois, avez-vous entendu ou vu un responsable communautaire ou religieux parler publiquement contre la planification familiale ?	OUI .... 1<br>NON .... 2<br>NE SE RAPPELLE PAS.......... 8	
Q717	Au cours de 12 derniers mois, avez-vous entendu ou vu un responsable communautaire ou religieux parler publiquement en faveur de la planification familiale?	OUI .... 1<br>NON .... 2<br>NE SE RAPPELLE PAS.......... 8	
Q718	Combien parmi vos proches amis/ les membres de votre famille diriez-vous qu'ils utilisent la PF/ Espacement des naissances: aucun, un peu, la plupart ou tous ?	AUCUN.......... 1<br>PEU D'ENTRE-EUX.......... 2<br>LA PLUPART.......... 3<br>TOUS.......... 4<br>NE SAIT PAS.......... 8	
Q719	Pensez-vous qu'il existe des membres de votre communauté qui vous traiteront de mauvaise personne ou fuiront votre compagnie s'ils savaient que vous utilisez une méthode de PF/Espacement des naissances ?	OUI .... 1<br>NON .... 2<br>NE SAIT PAS.......... 8	
Q720	Pensez-vous qu'il existe des membres de votre communauté qui vont vous féliciter, vous encourager ou dire du bien de vous s'ils savaient que vous utilisez une méthode de PF/Espacement des naissances ?	OUI .... 1<br>NON .... 2<br>NE SAIT PAS.......... 8	

	S'il vous plait, pouvez vous me dire si vous êtes totalement d'accord, d'accord, pas d'accord ou totalement contre les arguments suivants:	TOTALEMENT D'ACCORD	D'ACCORD	PAS D'ACCORD	TOTAL-EMENT CONTRE	
Q721	Vous pouvez commencer une conversion sur la PF avec votre partenaire/époux	4	3	2	1	
Q722	Vous pouvez convaincre votre partenaire que vous devez utiliser une méthode de PF	4	3	2	1	
Q723	Vous pouvez aller à un endroit où la PF est vendue/ offerte pour obtenir une méthode si vous décidez d'en avoir une.	4	3	2	1	
Q724	Vous pouvez obtenir une méthode de PF si vous décidez d'en avoir une	4	3	2	1	
Q725	Vous pouvez utiliser une méthode de PF même si votre partenaire ne le veut pas	4	3	2	1	
Q726	Vous pouvez utiliser une méthode de PF même si aucun de vos amis ou voisins n'en utilise	4	3	2	1	
Q727	Vous pouvez utiliser une méthode de PF même si votre leader religieux pense que vous ne devrez pas l'utiliser	4	3	2	1	
Q728	Vous pouvez continuer d'utiliser une méthode de PF même si vous avez eu à subir des effets secondaires	4	3	2	1	

Q729	S'il vous plait, en dehors de votre conjoint, donnez-moi les noms de 3 <u>principales</u> personnes avec qui vous vous sentez à l'aise pour discuter de problèmes personnels.<br><br>SI AUCUNE PERSONNE N'EST MENTIONNEE, ENREGISTRER 'AUCUNE PERSONNE'.	NOM 1 ____________________<br>NOM 2 ____________________<br>NOM 3 ____________________<br><br>AUCUNE PERSONNE: ☐ → Q736	

		Q730	Q731	Q732	Q733	Q734	Q735
N° PERSONNE	RECOPIER LES NOMS DES PERSONNES MENTIONNEES A Q729	Quelle relation entretenez-vous avec (NOM)?	Cette personne habite-t-elle dans cette ville, une autre ville ou en zone rurale (village) ?	Avez-vous discuté de PF avec (NOM) au cours des <u>12 derniers mois</u>?	(NOM) utilise t-elle actuellement uneméthode de PF ?	Quelle méthode (NOM) utilise t-elle ?	Selon vous, cette personne pense-t-elle que vous devrrez utiliser une méthode de PF ou non ?
**1**		MERE…………………… 01<br>BELLE-MERE………… 02<br>SŒUR/BELLE-SŒUR… 03<br>FILLE/BELLE-FILLE….. 04<br>UN AUTRE PARENT…. 05<br>AMIE…………………… 06<br>CONNAISSANCE……… 07<br>AUTRE_____________ 96<br>(PRECISER)	CETTE VILLE……..1<br>AUTRE VILLE……..2<br>ZONE RURALE……3<br>ETRANGER………. 4	OUI……… 1<br>NON……. 2<br>NSP…….. 8	OUI…………1<br>NON………. 2 → Q735<br>NSP………… 8	STERILISATION……… 1<br>PILULES……………… 2<br>DIU……………………. 3<br>INJECTABLES………. 4<br>PRESERVATIFS……… 5<br>AUTRE____________ 6<br>(PRECISER)<br>NE SAIT PAS………… 8	OUI, LE PENSE……… 1<br>NE LE PENSE PAS….. 2<br>NSP……………………. 8
**2**		MERE…………………… 01<br>BELLE-MERE………… 02<br>SŒUR/BELLE-SŒUR… 03<br>FILLE/BELLE-FILLE….. 04<br>UN AUTRE PARENT…. 05<br>AMIE…………………… 06<br>CONNAISSANCE……… 07<br>AUTRE_____________ 96<br>(PRECISER)	CETTE VILLE……..1<br>AUTRE VILLE……..2<br>ZONE RURALE……3<br>ETRANGER………. 4	OUI……… 1<br>NON……. 2<br>NSP…….. 8	OUI…………1<br>NON………. 2 → Q735<br>NSP………… 8	STERILISATION……… 1<br>PILULES……………… 2<br>DIU……………………. 3<br>INJECTABLES………. 4<br>PRESERVATIFS……… 5<br>AUTRE____________ 6<br>(PRECISER)<br>NE SAIT PAS………… 8	OUI, LE PENSE……… 1<br>NE LE PENSE PAS….. 2<br>NSP……………………. 8
**3**		MERE…………………… 01<br>BELLE-MERE………… 02<br>SŒUR/BELLE-SŒUR… 03<br>FILLE/BELLE-FILLE….. 04<br>UN AUTRE PARENT…. 05<br>AMIE…………………… 06<br>CONNAISSANCE……… 07<br>AUTRE_____________ 96<br>(PRECISER)	CETTE VILLE……..1<br>AUTRE VILLE……..2<br>ZONE RURALE……3<br>ETRANGER………. 4	OUI……… 1<br>NON……. 2<br>NSP…….. 8	OUI…………1<br>NON………. 2 → Q735<br>NSP………… 8	STERILISATION……… 1<br>PILULES……………… 2<br>DIU……………………. 3<br>INJECTABLES………. 4<br>PRESERVATIFS……… 5<br>AUTRE____________ 6<br>(PRECISER)<br>NE SAIT PAS………… 8	OUI, LE PENSE……… 1<br>NE LE PENSE PAS….. 2<br>NSP……………………. 8

INSTRUCTIONS: POSER LES QUESTIONS Q736, Q737, Q738 ET Q739 POUR CHAQUE LIGNE AVANT DE PASSER A LA LIGNE SUIVANTE.				
	Q736. Au cours des 12 dernières mois, avez-vous parlé d'espacement des naissances avec votre __________ (LIRE LES CATEGORIES CI-DESSOUS)	Q737. Quelle importance accordez-vous à l'avis de cette personne en ce qui concerne les questions d'espacement des naissances?	Q738. Pensez-vous que cette personne vous appuierait à utiliser une méthode de PF / d'espacement des naissances?	Q739. Avez-vous besoin d'avoir l'accord de cette personne pour utiliser une méthode de PF / Espacement des naissances
a) Mère	OUI....................................1<br>NON..................................2<br>NA………………..……..7 → à b)	PAS DU TOUT…………….1<br>QUELQUE PEU…………..2<br>BEAUCOUP………………..3	OUI....................................1<br>NON..................................2<br>NE SAIT PAS……..……..8	OUI....................................1<br>NON..................................2
b) Belle-mère	OUI....................................1<br>NON..................................2<br>NA……………………..7 → à c)	PAS DU TOUT…………….1<br>QUELQUE PEU…………..2<br>BEAUCOUP………………..3	OUI....................................1<br>NON..................................2<br>NE SAIT PAS……..……..8	OUI....................................1<br>NON..................................2
c) Sœur	OUI....................................1<br>NON..................................2<br>NA……………………..7 → à d)	PAS DU TOUT…………….1<br>QUELQUE PEU…………..2<br>BEAUCOUP………………..3	OUI....................................1<br>NON..................................2<br>NE SAIT PAS……..……..8	OUI....................................1<br>NON..................................2
d) Belle-soeur	OUI....................................1<br>NON..................................2<br>NA……………………..7 → à e)	PAS DU TOUT…………….1<br>QUELQUE PEU…………..2<br>BEAUCOUP………………..3	OUI....................................1<br>NON..................................2<br>NE SAIT PAS……..……..8	OUI....................................1<br>NON..................................2
e) Fille	OUI....................................1<br>NON..................................2<br>NA……………………..7 → à f)	PAS DU TOUT…………….1<br>QUELQUE PEU…………..2<br>BEAUCOUP………………..3	OUI....................................1<br>NON..................................2<br>NE SAIT PAS……..……..8	OUI....................................1<br>NON..................................2
f) Belle-fille	OUI....................................1<br>NON..................................2<br>NA……………………..7 → à g)	PAS DU TOUT…………….1<br>QUELQUE PEU…………..2<br>BEAUCOUP………………..3	OUI....................................1<br>NON..................................2<br>NE SAIT PAS……..……..8	OUI....................................1<br>NON..................................2
g) Amie	OUI....................................1<br>NON..................................2<br>NA……………………..7 → à h)	PAS DU TOUT…………….1<br>QUELQUE PEU…………..2<br>BEAUCOUP………………..3	OUI....................................1<br>NON..................................2<br>NE SAIT PAS……..……..8	OUI....................................1<br>NON..................................2
h) Cousine	OUI....................................1<br>NON..................................2<br>NA……………………..7 → à i)	PAS DU TOUT…………….1<br>QUELQUE PEU…………..2<br>BEAUCOUP………………..3	OUI....................................1<br>NON..................................2<br>NE SAIT PAS……..……..8	OUI....................................1<br>NON..................................2
i) Votre responsable religieux	OUI....................................1<br>NON..................................2<br>NA…………..7 → Q740	PAS DU TOUT…………….1<br>QUELQUE PEU…………..2<br>BEAUCOUP………………..3	OUI....................................1<br>NON..................................2<br>NE SAIT PAS……..……..8	OUI....................................1<br>NON..................................2

Q740	A votre avis, est-il acceptable pour une femme d'utiliser la contraception à l'insu de son mari ou de son partenaire si ?	OUI	NON	NE SAIT PAS
	a. Son mari est contre l'utilisation de la contraception, mais elle insiste pour en utiliser.	1	2	8
	b. Ils ont beaucoup d'enfants.	1	2	8
	c. Son mari est violent envers elle ou ses enfants.	1	2	8
	d. Le couple ne possède pas assez d'argent pour pouvoir s'occuper d'autres enfants.	1	2	8

SECTION 8 MESURES D'INEGALITE BASEE SUR LE SEXE

Maintenant, je voudrais vous poser des questions concernant la manière dont vous et votre partenaire prenez les décisions concernant votre ménage. S'il vous plaît, rappelez-vous que vous devez être le plus honnête possible et que vos réponses resteront confidentielles ; c'est-à-dire, que personne ne verra vos réponses.

Q801	**VERIFIER Q511 ET Q512:**<br><br>MARIEE OU VIT ACTUELLEMENT AVEC UN HOMME : ☐ ↓<br>(Q512=1 OU 2)<br><br>PAS EN UNION: ☐ → Q811<br>(Q511=3 OU Q512=3,4,5)		Q811
Q802	Quel âge avait votre partenaire lors de son dernier anniversaire?	AGE EN ANNEES REVOLUES …………………. [ ][ ]<br>NE SAIT PAS…………………………………… 98	
Q803	Votre (dernier) mari/ partenaire a t-il déjà fréquenté l'école ?	OUI …………………………………………… 1<br>NON …………………………………………… 2	 → Q806
Q804	Quel était le niveau scolaire le plus élevé qu'il avait atteint: primaire, secondaire ou supérieur?	PRIMAIRE…………………………………… 1<br>SECONDAIRE 1…………………………………… 2<br>SECONDAIRE 2…………………………………… 3<br>SUPERIEUR…………………………………… 4<br>NE SAIT PAS…………………………………… 8	 <br><br>→ Q806<br>→ Q806
Q805	Quelle était la classe/l'année la plus élevée de ce niveau qu'il a achevée ?	CLASSE…………………………………… … [ ][ ]<br>NE SAIT PAS…………………………………… 98	
Q806	Quelle est l'occupation de votre partenaire, c'est-à-dire quel type de travail fait-il <u>principalement</u>, <u>lorsqu'il lui arrive de travailler</u> ?	PROFESSIONNEL/TECHNICIENN/<br>ADMINISTRATIF…………………………… 1<br>VENTES ET SERVICES…………………………… 2<br>TRAVAIL MANUEL NON AGRICOLE…………… 3<br>AGRICULTURE…………………………………… 4<br>AUTRE ______________________ 6<br>(A PRECISER)	
Q807	Est-ce que votre partenaire/époux <u>travaille actuellement</u> ?	OUI …………………………………………… 1<br>NON …………………………………………… 2<br>NE SAIT PAS…………………………………… 8	→ Q809
Q808	Au cours des 12 derniers mois, qu'a t-il fait la <u>plupart de son temps</u>?	RIEN…………………………………………… 01<br>A LA RECHERCHE D'UN EMPLOI…………………… 02<br>ALLER A L'ECOLE/ ETUDIER …………………… 03<br>INCAPABLE DE TRAVAILLER/ HANDICAPE……… 04<br>RETRAITE …………………………………… 05<br>TRAVAUX DOMESTIQUES/S'OCCUPER<br>DES ENFANTS …………………………………… 06<br>AUTRE______________________ 96<br>(A PRECISER)	} Q811
Q809	Travaille t-il d'habitude pendant toute l'année, de manière saisonnière ou seulement de temps en temps?	PENDANT TOUTE L'ANNEE…………………………… 1<br>DE MANIERE SAISONNIERE/UNE PARTIE DE L'ANNEE………… 2<br>DE TEMPS EN TEMPS…………………………… 3	
Q810	Est-il payé <u>en espèces</u> pour son travail?	OUI…………………………………………… 1<br>NON…………………………………………… 2<br>NE SAIT PAS…………………………………… 8	

Q811	Quel est votre occupation, c'est-à-dire, quel sorte de travail faites-vous principalement lorsqu'il vous arrive de travailler?	NOM DE L'OCCUPATION ACTUELLE<br>________________<br>________________<br>CODE (A REMPLIR AU BUREAU).... ☐☐	
Q812	Comme vous le savez, certaines femmes exercent des emplois pour lesquels elles sont payées en espèces ou en nature. D'autres vendent des produits ou ont un petit commerce, d'autres travaillent dans le champ familial ou dans le commerce de la famille.<br><br>Au cours des 7 derniers jours, avez-vous fait une de ces choses ou tout autre travail ?	OUI .... 1<br>NON 2	1 → Q814
Q813	Avez-vous travaillé au cours des 12 derniers mois?	OUI .... 1<br>NON 2	2 → Q819
Q814	Travaillez-vous pour un membre de votre famille, pour quelqu'un d'autre ou vous auto-employez-vous ?	POUR UN MEMBRE DE LA FAMILLE.................. 1<br>POUR QUELQU'UN D'AUTRE.................. 2<br>S'AUTO-EMPLOIE.................. 3	3 → Q817
Q815	Est-ce que votre employeur met des services de santé à votre disposition au lieu travail ?	OUI .... 1<br>NON .... 2<br>NE SAIT PAS.................................. 8	
Q816	Pourriez-vous accéder à des services de PF/ contraception si votre employeur les mettait à votre disposition (à votre lieu de travail)?	OUI .... 1<br>NON .... 2<br>NE SAIT PAS.................................. 8	
Q817	Travaillez-vous d'habitude pendant toute l'année, de manière saisonnière, ou seulement de temps en temps?	PENDANT TOUTE L'ANNEE.................. 1<br>DE MANIERE SAISONNIERE/UNE<br>PARTIE DE L'ANNEE.................. 2<br>DE TEMPS EN TEMPS.................. 3	
Q818	Etes- vous payée en espèces ou en nature pour ce travail, ou n'êtes-vous pas payée du tout?	SEULEMENT EN ESPECES.................. 1<br>EN ESPECES ET EN NATURE.................. 2<br>SEULEMENT EN NATURE.................. 3<br>PAS PAYE.................. 4	
Q819	**VERIFIER Q511 ET Q512: DEJA MARIE ET ACTUELLEMENT MARIE**<br>MARIEE OU VIT ACTUELLEMENT AVEC UN HOMME (Q512=1 OU 2) : ☐ ↓	PAS EN UNION (Q511=3 OU Q512=3, 4 OU 5) : ☐	→ Q825
Q820	**VERIFIER Q813 ET Q818:**<br>SI ELLE TRAVAILLE ACTUELLEMENT ET GAGNE DE L'ARGENT (Q818=1 OU 2): ☐ ↓	SI ELLE NE TRAVAILLE PAS ACTUELLEMENT OU NE GAGNE PAS D'ARGENT : (Q813=2 OU Q818=3 OU 4) ☐	→ Q822
Q821	Qui décide de comment utiliser l'argent que vous gagnez : principalement vous, principalement votre partenaire, ou vous et votre partenaire conjointement ?	REPONDANTE .... 1<br>PARTENAIRE.................................. 2<br>REPONDANTE ET EPOUX/PARTENAIRE<br>CONJOINTEMENT.................. 3<br>AUTRE________________ 6<br>(A PRECISER)	

Q822	**VERIFIER Q807 ET Q810:**<br><br>S'IL TRAVAILLE ACTUELLEMENT ET GAGNE DE L'ARGENT : ☐<br>(Q807=1 ET Q810=1) ↓	S'IL NE TRAVAILLE PAS ACTUELLEMENT OU NE GAGNE PAS D'ARGENT : (Q807=2 OU Q810=2, 8) ☐	→Q824
Q823	Qui décide de comment utiliser l'argent que votre partenaire gagne : principalement vous, principalement votre partenaire, ou vous et votre partenaire conjointement ?	REPONDANTE …………………………………………… 1<br>PARTENAIRE……………………………………………… 2<br>REPONDANTE ET EPOUX/PARTENAIRE CONJOINTEMENT…………………………………………… 3<br>AUTRE________________________________ 6<br>(A PRECISER)	

Q824	Parfois dans le mariage ou relation amoureuse, l'homme interdit à la femme de faire certaines choses.<br><br>Votre mari / partenaire vous interdit-il de :		OUI	NON
		a. Travailler en dehors de la maison ?	1	2
		b. Avoir des visites ?	1	2
		c. Rendre visite à vos amis ?	1	2
		d. Rendre visite à votre famille ?	1	2
		e. Utiliser une methode de contraception?	1	2
		f. Utiliser un téléphone mobile ?	1	2

Q825	Avez-vous de l'argent propre et pour lequel vous pouvez décider seule de la manière de le dépenser ?	OUI …………………………………………………………… 1<br>NON …………………………………………………………… 2

Q826	Dans un couple, qui pensez-vous devrait avoir le dernier mot dans chacune des décisions suivantes :	MARI	FEMME	LES DEUX EGALEMENT	NE SAIT PAS/ DEPEND
	a. Effectuer les grandes courses de la maison ?	1	2	3	8
	b. Effectuer les petites courses quotidiennes du ménage ?	1	2	3	8
	c. Décider de quand rendre visite à la famille, aux amis, ou aux parents ?	1	2	3	8
	d. Décider de quand et où chercher des soins médicaux pour la famme?	1	2	3	8

Q827	Souvent, un partenaire est contrarié ou vexé par certaines choses que fait sa femme. A votre avis, est-il justifié qu'un partenaire frappe sa femme dans les situations suivantes?	OUI	NON	NE SAIT PAS
	a. Si elle sort sans lui dire?	1	2	8
	b. Si elle néglige la maison ou les enfants?	1	2	8
	c. Si elle dispute avec lui?	1	2	8
	d. Si elle refuse d'avoir des rapports sexuels avec lui?	1	2	8
	e. Si elle ne prépare pas bien la nourriture?	1	2	8
	f. Si elle le suspecte d'être infidèle ?	1	2	8
	g. Si elle refuse d'avoir un autre enfant?	1	2	8

	Maintenant, je vais vous lire des déclarations concernant les normes basées sur le sexe et les motivations de fécondité. Pour chaque déclaration, SVP, dites-moi si vous approuvez fortement, approuvez quelque peu, désapprouvez quelque peu ou désapprouvez fortement avec ce qui est dit.				
		APPROUVE FORTEMENT	APPROUVE QUELQUE PEU	DESAPPROUVE QUELQUE PEU	DESAPPROUVE FORTEMENT
Q828	Le mari devrait être celui qui décide si le couple devrait utiliser une méthode d'espacement des naissances/de planification familiale.	**4**	**3**	**2**	**1**
Q829	Les couples qui pratiquent l'espacement des naissances/la planification familiale ont une meilleure qualité de vie que ceux qui n'en pratiquent pas.	**4**	**3**	**2**	**1**
Q830	Les maris et les femmes devraient discuter de la contraception/espacement des naissances.	**4**	**3**	**2**	**1**
Q831	Les hommes ne devraient pas permettre à leurs femmes d'utiliser la contraception.	**4**	**3**	**2**	**1**
Q832	Une femme qui utilise la contraception à l'insu de son mari devrait être punie.	**4**	**3**	**2**	**1**
Q833	Une femme qui n'a pas d'enfants n'est pas une femme complète.	**4**	**3**	**2**	**1**
Q834	Un homme qui n'a pas d'enfants n'est pas un homme complet.	**4**	**3**	**2**	**1**
Q835	C'est bien d'avoir beaucoup d'enfants parce que personne ne sait lequel d'entre eux survivra ou sera riche pour s'occuper de ses parents lorsque ceux-ci seront vieux.	**4**	**3**	**2**	**1**
Q836	C'est à Dieu seul de décider du nombre d'enfants qu'un couple aura.	**4**	**3**	**2**	**1**
Q837	Une femme devrait continuer de faire des enfants jusqu'à ce qu'elle ait au moins un garçon.	**4**	**3**	**2**	**1**
Q838	Une femme devrait continuer de faire des enfants jusqu'à ce qu'elle ait au moins une fille.	**4**	**3**	**2**	**1**
SQ23	L'espacement des naissances aide les parents à mieux prendre soin de leurs enfants.	**4**	**3**	**2**	**1**
SQ24	L'utilisation de l'espacement des naissances est permise seulement dans le souci de la santé de la mère et de l'enfant.	**4**	**3**	**2**	**1**
SQ25	Les maris et les femmes devraient discuter du nombre d'enfants qu'ils veulent avoir.	**4**	**3**	**2**	**1**

SECTION 9: EXPOSITION AUX MEDIA

	Maintenant, je voudrais vous parler de vos besoins en informations et des endroits où obtenir des informations sur la PF.		
Q901	Quelles sont vos principales sources d'informations sur la santé ?<br><br>**INSISTER SEPAREMENT POUR :**<br>A. SOURCES MEDIATIQUES<br>B. SOURCES MEDICALES<br>C. SOURCES COMMUNAUTAIRES<br>D. SOURCES INTERPERSONNELLES<br><br>ENREGISTRER TOUT CE QUI EST MENTIONNE.	**SOURCES MEDIATIQUES**<br>RADIO…… AA<br>TV…… AB<br>JOURNAUX…… AC<br>MAGAZINES…… AD<br>PANNEAUX D'AFFICHAGE…… AE<br>PEINTURE MURALE…… AF<br>INTERNET …… AG<br><br>**SOURCES MEDICALES**<br>MEDECIN…… BA<br>SAGE-FEMME…… BB<br>INFIRMIER/AI…… BC<br>MATRONE / ASC …… BD<br>GUERISSEUR / ACCOUCHEUSE TRADITIONNELLE…… BE<br>PHARMACIEN …… BF<br><br>**SOURCES COMMUNAUTAIRES**<br>RELAI COMMUNAUTAIRE …… CA<br>ORGANISATION COMMUNAUTAIRE DE BASE (OCB)…… CB<br>VOLONTAIRES / PAIRS ÉDUCATEURS …… CC<br>ECOLE…… CD<br>TRAVAILLEUR D'ONG…… CE<br><br>**SOURCES INTERPERSONNELLES**<br>PARENTS…… DA<br>BEAUX-PARENTS…… DB<br>EPOUX/PARTENAIRE…… DC<br>FRERES ET SŒURS…… DD<br>BELLES SŒURS/BEAUX-FRERES…… DE<br>AMIS / VOISINS…… DF<br>AUTRES PARENTS/CONNAISSANCES…… DG<br><br>**AUTRE (PRECISER)**__________ XX<br>**AUCUN……** YY<br>**NE SAIT PAS ……** ZZ	
Q902	Lisez-vous des journaux ?	OUI …… 1<br>NON …… 2	 → Q904
Q903	Quel(s) journal (ux) lisez-vous le plus souvent?	L'OBSERVATEUR…… A<br>LE POPULAIRE…… B<br>LE QUOTIDIEN…… C<br>LE SOLEIL…… D<br>SUD QUOTIDIEN…… E<br>WALF QUOTIDIEN…… F<br>L'AS…… G<br>KOTCH…… H<br>LE MESSAGER…… I<br>WALF GRAND PLACE…… J<br>LE MATIN…… K<br>AUTRE JOURNAL __________ X<br>(A PRECISER)	
Q904	Lisez-vous des magazines?	OUI …… 1<br>NON …… 2	 → Q906

Q905	Quel(s) magazine(s) lisez-vous le plus souvent ?	WEEK-END…… A<br>THIOF…… B<br>LISSA…… C<br>ICONE…… D<br>DAKAR LIFE…… E<br>LIFA…… F<br>AUTRE ______ X<br>(A PRECISER)	
Q906	**VERIFIER: Q902 ET 904**<br>SI OUI A L'UNE OU L'AUTRE : ☐ ↓ SI NON AUX DEUX : ☐ →		Q909
Q907	Avez-vous lues des informations sur la PF dans les journaux/magazines au cours des 3 derniers mois ?	OUI …… 1<br>NON …… 2	 → Q909
Q908	Quelles informations avez-vous lues dans les journaux/magazines sur la planification familiale ?<br><br>INSISTER: D'autres informations?<br><br>ENCERCLER TOUT CE QUI EST MENTIONNE.<br><br>SI PILULE, INSISTER POUR SAVOIR SI PILULE JOURNALIERE OU CONTRACEPTION D'URGENCE.	PILULES…… A<br>DIU…… B<br>PRESERVATIFS…… C<br>INJECTABLES …… D<br>IMPLANT…… E<br>CONTRACEPTION D'URGENCE…… F<br>STERILISATION FEMININE…… G<br>STERILISATION MASCULINE…… H<br>ALLAITEMENT MATERNEL…… I<br>METHODE DES JOURS FIXES…… J<br>AGE DU MARIAGE…… K<br>RETARDER L'AGE DU 1ER RAPPORT SEXUEL…… L<br>RETARDER LA PREMIERE NAISSANCE…… M<br>ESPACEMENT DES NAISSANCES…… N<br>LIMITATION DE LA TAILLE DE LA FAMILLE…… O<br>AUTRES______ X<br>(PRECISER)	
Q909	Ecoutez-vous la radio?	OUI …… 1<br>NON …… 2	 → Q916
Q910	Quelles stations de radio écoutez-vous?<br><br>INSISTER: quelles autres stations radio?<br><br>ENCERCLER TOUT CE QUI EST MENTIONNE.	RTS…… A<br>WALF FM…… B<br>SUD FM…… C<br>DUNYA FM…… D<br>RFM…… E<br>ZIK FM…… F<br>OXYJEUNES…… G<br>X-FM…… H<br>ALFAIDA…… I<br>NDEFLENG…… J<br>SOXNA FM…… K<br>DJIDA FM…… L<br>LA COTIERE…… M<br>RFI…… N<br>AUTRE______ X<br>(PRECISER)	
Q911	Combien de jours par semaine écoutez-vous la radio?	NOMBRE DE JOURS PAR SEMAINE…… ☐<br>PAS REGULIEREMENT…… 8	

Q912	En moyenne combien de temps au total écoutez-vous la radio dans un jour normal ?	HEURES PAR JOUR.......................... ....1 [0][ ][ ]<br>OU<br>MINUTES PAR JOUR.......................... .....2 [ ][ ][ ]	
Q913	Habituellement, à quel moment écoutez-vous le plus la radio?	LE MATIN (4H - 12H)..1<br>L'APRES-MIDI (12H - 18H).. 2<br>LE SOIR (18H - 22H).. 3<br>TARD LE SOIR (22H - 4H)..4	
Q914	Avez-vous entendues des informations sur la PF à la radio au cours des trois derniers mois?	OUI.. 1<br>NON.. 2	 →Q916
Q915	Quelles informations avez-vous entendues à la radio sur la planification familiale?<br><br>INSISTER: D'autres informations?<br><br>ENCERCLER TOUT CE QUI EST MENTIONNE.<br><br>SI PILULE, INSISTER POUR SAVOIR SI PILULE JOURNALIERE OU CONTRACEPTION D'URGENCE.	PILULES.. A<br>DIU.. B<br>PRESERVATIFS.. C<br>INJECTABLES .... D<br>IMPLANT.. E<br>CONTRACEPTION D'URGENCE.. F<br>STERILISATION FEMININE.. G<br>STERILISATION MASCULINE.. H<br>ALLAITEMENT MATERNEL.. I<br>METHODE DES JOURS FIXES.. J<br>AGE DU MARIAGE.. K<br>RETARDER L'AGE DU 1ER RAPPORT SEXUEL.. L<br>RETARDER LA PREMIERE NAISSANCE.. M<br>ESPACEMENT DES NAISSANCES.. N<br>LIMITATION DE LA TAILLE DE LA FAMILLE.. O<br>AUTRES ______________________ X<br>(PRECISER)	
Q916	A votre avis, les messages sur les thèmes suivants à la radio sont-ils acceptables ou non ?<br>a. Planification familial/espacement des naissances<br>b. VIH/SIDA<br>c. Santé maternelle (soins prenatals, acouchement,…)<br>d. Santé infantile (vaccination, soins préventifs,…)<br>e. Santé de reproduction (ISTs, infécondité)	ACCEPTABLE / INACCEPTABLE<br>a. 1 2<br>b. 1 2<br>c. 1 2<br>d. 1 2<br>e. 1 2	
Q917	Regardez-vous la télévision?	OUI .... 1<br>NON 2	 → Q926
Q918	Combien de jours par semaine regardez-vous la télévision?	NOMBRE DE JOURS PAR SEMAINE.. [ ]<br>PAS REGULIEREMENT.. 8	
Q919	En moyenne combien de temps au total regardez-vous la télévision dans un jour normal ?	HEURES PAR JOUR.......................... ....1 [0][ ][ ]<br>OU<br>MINUTES PAR JOUR.......................... .....2 [ ][ ][ ]	
Q920	Habituellement à quel moment regardez-vous le plus la télévision?	LE MATIN (4H - 12H)..1<br>L'APRES-MIDI (12H - 18H).. 2<br>LE SOIR (18H - 22H).. 3<br>TARD LE SOIR (22H - 4H)..4	
Q921	En général, où regradez-vous la télé ?	CHEZ MOI .... 1<br>CHEZ LES PARENTS/AMIS/VOISINS.. 2<br>CENTRE CULTUREL / FOYER /ESPACE PUBLIQUE.. 3<br>BAR/RESTAURANT.. 4<br>AUTRE______________________ 6<br>(PRECISER)	

Q922	Quelles chaînes regardez-vous <u>généralement</u> à la télévision?<br><br>INSISTER: D'autres chaines de TV?<br><br>ENREGISTER TOUT CE QUI EST MENTIONNE.	RTS 1 ... A<br>WALF TV ... B<br>SN2 ... C<br>RDV ... D<br>2STV ... E<br>AFRICABLE ... F<br>CANAL INFO ... G<br>CANAL HORIZONS ... H<br>TFM ... I<br>TV5 ... J<br>FRANCE24 ... K<br>AUTRES ___ X<br>(PRECISER)	
Q923	Quels types de programme TV regradez-vous <u>en général</u> ?<br><br>INSISTER: Quels autres type de programme?<br><br>ENCERCLER TOUT CE QUI EST MENTIONNE.	INFOS/ACTUALITES ... A<br>PROGRAMME RELIGIEUX ... B<br>DEBATS POLITIQUES ... C<br>VARIETES ET TALK SHOWS ... D<br>PROGRAMMES NATURE ... E<br>DOCUMENTAIRES ... F<br>THEATRES/FILMS/FEULLETONS ... G<br>DESSINS ANIMES ... H<br>CLIPS MUSIQUE ... I<br>SPORT ... J<br>AUTRE ___ X<br>(PRECISER)	
Q924	Avez-vous vues des informations sur laPF à la télévision au cours des <u>trois derniers mois</u> ?	OUI ... 1<br>NON ... 2	 → Q926
Q925	Quelles informations avez-vous vues à la télévision sur la planification familiale?<br><br>INSISTER: D'autres informations?<br><br>ENCERCLER TOUT CE QUI EST MENTIONNE.<br><br>SI PILULE, INSISTER POUR SAVOIR SI PILULE JOURNALIERE OU CONTRACEPTION D'URGENCE.	PILULES ... A<br>DIU ... B<br>PRESERVATIFS ... C<br>INJECTABLES ... D<br>IMPLANT ... E<br>CONTRACEPTION D'URGENCE ... F<br>STERILISATION FEMININE ... G<br>STERILISATION MASCULINE ... H<br>ALLAITEMENT MATERNEL ... I<br>METHODE DES JOURS FIXES ... J<br>AGE DU MARIAGE ... K<br>RETARDER L'AGE DU 1ER RAPPORT SEXUEL ... L<br>RETARDER LA PREMIERE NAISSANCE ... M<br>ESPACEMENT DES NAISSANCES ... N<br>LIMITATION DE LA TAILLE DE LA FAMILLE ... O<br>AUTRE ___ X<br>(PRECISER)	

Q926 A votre avis, les messages sur les thèmes suivants à la télévision sont-ils acceptables ou non ?

		ACCEPTABLE	INACCEPTABLE
a. Planification familial/espacement des naissances	a.	1	2
b.VIH/SIDA	b.	1	2
c. Santé maternelle (soins prenatals, acouchement,…)	c.	1	2
d. Santé infantile (vaccination, soins préventifs,…)	d.	1	2
e. Santé de reproduction (ISTs, infécondité)	e.	1	2

Q927	Allez-vous au cinéma?	OUI ... 1<br>NON ... 2	 → Q930

Q928	Selon quelle fréquence allez-vous au cinéma ?	PLUS D'UNE FOIS PAR SEMAINE………………………………… 1<br>UNE FOIS PAR SEMAINE……………………………………… 2<br>QUELQUES FOIS PAR MOIS……………………………………… 3<br>AU MOINS UNE FOIS PAR MOIS………………………………… 4<br>AU MOINS UNE FOIS PAR AN……………………………………… 5<br>RAREMENT………………………………………………………… 6	
Q930	Avez-vous personnellement un téléphone portable à votre propre usage?	OUI ……………………………………………………………… 1<br>NON ……………………………………………………………… 2<br>NE CONNAIT PAS TELEPHONE PORTABLE……………… 8	→ Q932<br><br>→ Q936b
Q931	Avez-vous accès au téléphone portable?	OUI ……………………………………………………………… 1<br>NON ……………………………………………………………… 2	
Q932	Avez-vous jamais eu une conversation téléphonique sur la PF ?	OUI ……………………………………………………………… 1<br>NON ……………………………………………………………… 2	 → Q934
Q933	Est-ce que la/les personne(s) à qui vous avez parlé de PF par téléphone est/sont dans cette ville (site), dans une autre ville, en zone rurale ou un autre pays ?<br><br>ENCERCLER TOUT CE QUI EST MENTIONNE.	CETTE VILLE (SITE)……………………………………………… A<br>UNE AUTRE VILLE………………………………………………… B<br>ZONE RURALE……………………………………………………… C<br>AUTRE PAYS ETRANGER…………………………………………… D<br>NE SAIT PAS………………………………………………………… Z	
Q934	Seriez-vous à l'aise en recevant des messages sur la PF /Contraception ou la santé par SMS ?	OUI ……………………………………………………………… 1<br>NON ……………………………………………………………… 2<br>NE CONNAIT PAS SMS……………………………………………… 8	 <br>→ Q936a
Q935	Au cours des <u>3 derniers mois</u>, avez-vous reçu des messages SMS sur la PF/Contraception ?	OUI ……………………………………………………………… 1<br>NON ……………………………………………………………… 2	
Q936	Au cours des <u>3 derniers mois</u>, avez-vous accédé à internet,web, ou email au moins par l'un des moyens suivants :<br>a. Téléphone portable	OUI ……………………………………………………………… 1<br>NON ……………………………………………………………… 2<br>NE CONNAIT PAS INTERNET………………………………… 8	 <br>→ Q937
	b. Ordinateur	OUI ……………………………………………………………… 1<br>NON ……………………………………………………………… 2	
Q937	Etes-vous membre d'une association, un groupe, une organisation?	OUI ……………………………………………………………… 1<br>NON ……………………………………………………………… 2	 → Q1001

938<br>Quels sont les noms et types d'organisations dont vous êtes membre ?	939<br>Depuis combien de temps êtes-vous membre ?	940<br>A quelle périodicité cette organisation se réunit-elle ?	*941*<br>A quelle fréquence discutez-vous avec des membres de cette organisation en dehors de vos rencontres au sein de l'organisation ?	942<br>A quelle fréquence assistez-vous aux réunions de l'organisation ?	943<br>Avez-vous jamais vu ou entendu des information sur la PF au cours des rencontres de l'organisation?	944<br>Quelles informations sur la PF avez-vous vu/entendu au cours des rencontres de l'organisation ?
1)<br>________________<br>NOM ORGANISATION<br>________________<br>TYPE D'ORGANISATION<br>(Groupement de femmes,<br>Organisation sportive,<br>Groupe de théâtre,<br>Association religieuse, etc.)<br>A CODER AU BUREAU	< 1 ANS .... 1<br>1 - 2 ANS ...... 2<br>3 - 5 ANS ...... 3<br>6 ANS ET PLUS. 4<br>NSP.................. 8	CHAQUE JOUR....... 1<br>CHAQUE SEMAINE. 2<br>CHAQUE MOIS .... 3<br>CHAQUE ANNEE .... 4<br>PAS REGULIER ...... 5<br>AUTRE__________ 6<br>(PRECISER)	CHAQUE JOUR...... 1<br>CHAQUE SEMAINE. 2<br>CHAQUE MOIS ...... 3<br>CHAQUE ANNEE ... 4<br>PAS REGULIER ..... 5<br>AUTRE_________. 6<br>(PRECISER)	TOUJOURS..... 1<br>SOUVENT....... 2<br>PARFOIS......... 3<br>RAREMENT..... 4<br>JAMAIS........... 5	OUI .... 1<br>NON................... 2<br>LIGNE SUIV. ←<br>NSP.................... 8	PILULES.. A<br>DIU.. B<br>PRESERVATIFS...................................... C<br>INJECTABLES .... D<br>IMPLANT.. E<br>CONTRACEPTION D'URGENCE.............. F<br>STERILISATION FEMININE..................... G<br>STERILISATION MASCULINE.................. H<br>ALLAITEMENT MATERNEL...................... I<br>METHODE DES JOURS FIXES................ J<br>AGE DU MARIAGE................................ K<br>RETARDER L'AGE DU 1ER RAPPORT SEXI L<br>RETARDER LA PREMIERE NAISSANCE.... M<br>ESPACEMENT DES NAISSANCES........... N<br>LIMITATION DE LA TAILLE DE LA FAMILLE O<br>AUTRES__________________________ X<br>(PRECISER)
2)<br>________________<br>NOM ORGANISATION<br>________________<br>TYPE D'ORGANISATION<br>(Groupement de femmes,<br>Organisation sportive,<br>Groupe de théâtre,<br>Association religieuse, etc.)<br>A CODER AU BUREAU	< 1 ANS .... 1<br>1 - 2 ANS ...... 2<br>3 - 5 ANS ...... 3<br>6 ANS ET PLUS. 4<br>NSP.................. 8	CHAQUE JOUR....... 1<br>CHAQUE SEMAINE. 2<br>CHAQUE MOIS .... 3<br>CHAQUE ANNEE .... 4<br>PAS REGULIER ...... 5<br>AUTR___________ 6<br>(PRECISER)	CHAQUE JOUR...... 1<br>CHAQUE SEMAINE. 2<br>CHAQUE MOIS ...... 3<br>CHAQUE ANNEE ... 4<br>PAS REGULIER ..... 5<br>AUTRE_________. 6<br>(PRECISER)	TOUJOURS..... 1<br>SOUVENT....... 2<br>PARFOIS......... 3<br>RAREMENT..... 4<br>JAMAIS........... 5	OUI .... 1<br>NON................... 2<br>LIGNE SUIV. ←<br>NSP.................... 8	PILULES.. A<br>DIU.. B<br>PRESERVATIFS...................................... C<br>INJECTABLES .... D<br>IMPLANT.. E<br>CONTRACEPTION D'URGENCE.............. F<br>STERILISATION FEMININE..................... G<br>STERILISATION MASCULINE.................. H<br>ALLAITEMENT MATERNEL...................... I<br>METHODE DES JOURS FIXES................ J<br>AGE DU MARIAGE................................ K<br>RETARDER L'AGE DU 1ER RAPPORT SEXI L<br>RETARDER LA PREMIERE NAISSANCE.... M<br>ESPACEMENT DES NAISSANCES........... N<br>LIMITATION DE LA TAILLE DE LA FAMILLE O<br>AUTRES__________________________ X<br>(PRECISER)

**938**<br>Quels sont les noms et types d'organisations dont vous êtes membre ?	**939**<br>Depuis combien de temps êtes-vous membre ?	**940**<br>A quelle périodicité cette organisation se réunit-elle ?	***941***<br>A quelle fréquence discutez-vous avec des membres de cette organisation en dehors de vos rencontres au sein de l'organisation ?	**942**<br>A quelle fréquence assistez-vous aux réunions de l'organisation ?	**943**<br>Avez-vous jamais vu ou entendu des information sur la PF au cours des rencontres de l'organisation?	**944**<br>Quelles informations sur la PF avez-vous vu/entendu au cours des rencontres de l'organisation ?
3)<br>_______________<br>NOM ORGANISATION<br>_______________<br>TYPE D'ORGANISATION<br>(Groupement de femmes,<br>Organisation sportive,<br>Groupe de théâtre,<br>Association religieuse, etc.)<br>A CODER AU BUREAU<br>[ ][ ]	< 1 ANS .... 1<br>1 - 2 ANS ...... 2<br>3 - 5 ANS ...... 3<br>6 ANS ET PLUS. 4<br>NSP................. 8	CHAQUE JOUR....... 1<br>CHAQUE SEMAINE. 2<br>CHAQUE MOIS .... 3<br>CHAQUE ANNEE .... 4<br>PAS REGULIER ...... 5<br>AUTR___________ 6<br>(PRECISER)	CHAQUE JOUR...... 1<br>CHAQUE SEMAINE. 2<br>CHAQUE MOIS ...... 3<br>CHAQUE ANNEE ... 4<br>PAS REGULIER ..... 5<br>AUTRE_________. 6<br>(PRECISER)	TOUJOURS..... 1<br>SOUVENT....... 2<br>PARFOIS......... 3<br>RAREMENT..... 4<br>JAMAIS........... 5	OUI .... 1<br>NON.................. 2<br>LIGNE SUIV. ←<br>NSP.................... 8	PILULES.. A<br>DIU... B<br>PRESERVATIFS.................................... C<br>INJECTABLES .... D<br>IMPLANT.. E<br>CONTRACEPTION D'URGENCE.............. F<br>STERILISATION FEMININE...................... G<br>STERILISATION MASCULINE.................. H<br>ALLAITEMENT MATERNEL...................... I<br>METHODE DES JOURS FIXES................ J<br>AGE DU MARIAGE................................ K<br>RETARDER L'AGE DU 1ER RAPPORT SEXI L<br>RETARDER LA PREMIERE NAISSANCE.... M<br>ESPACEMENT DES NAISSANCES........... N<br>LIMITATION DE LA TAILLE DE LA FAMILLE O<br>AUTRES___________________________ X<br>(PRECISER)
4)<br>_______________<br>NOM ORGANISATION<br>_______________<br>TYPE D'ORGANISATION<br>(Groupement de femmes,<br>Organisation sportive,<br>Groupe de théâtre,<br>Association religieuse, etc.)<br>A CODER AU BUREAU<br>[ ][ ]	< 1 ANS .... 1<br>1 - 2 ANS ...... 2<br>3 - 5 ANS ...... 3<br>6 ANS ET PLUS. 4<br>NSP................. 8	CHAQUE JOUR....... 1<br>CHAQUE SEMAINE. 2<br>CHAQUE MOIS .... 3<br>CHAQUE ANNEE .... 4<br>PAS REGULIER ...... 5<br>AUTR___________ 6<br>(PRECISER)	CHAQUE JOUR...... 1<br>CHAQUE SEMAINE. 2<br>CHAQUE MOIS ...... 3<br>CHAQUE ANNEE ... 4<br>PAS REGULIER ..... 5<br>AUTRE_________. 6<br>(PRECISER)	TOUJOURS..... 1<br>SOUVENT....... 2<br>PARFOIS......... 3<br>RAREMENT..... 4<br>JAMAIS........... 5	OUI .... 1<br>NON.................. 2<br>LIGNE SUIV. ←<br>NSP.................... 8	PILULES.. A<br>DIU... B<br>PRESERVATIFS.................................... C<br>INJECTABLES .... D<br>IMPLANT.. E<br>CONTRACEPTION D'URGENCE.............. F<br>STERILISATION FEMININE...................... G<br>STERILISATION MASCULINE.................. H<br>ALLAITEMENT MATERNEL...................... I<br>METHODE DES JOURS FIXES................ J<br>AGE DU MARIAGE................................ K<br>RETARDER L'AGE DU 1ER RAPPORT SEXI L<br>RETARDER LA PREMIERE NAISSANCE.... M<br>ESPACEMENT DES NAISSANCES........... N<br>LIMITATION DE LA TAILLE DE LA FAMILLE O<br>AUTRES___________________________ X<br>(PRECISER)

SECTION 10: MOUVEMENTS MIGRATOIRES			
	Maintenant, je voudrais vous demander depuis combien de temps vous vivez ici et d'où vous venez et à quelle fréquence vous visitez d'autres régions.		
Q1001	Depuis combien de temps vivez-vous de manière continue dans cette maison ou cet appartement?<br><br>SI DE QUELQUES MOIS A 11 MOIS, ENREGISTRER 00-11. AUTREMENT, ENREGISTRER NOMBRE D'ANNEES.	MOIS………… 1 [ ][ ]<br>ANNEES………… 2 [ ][ ]<br>DEPUIS TOUJOURS………… 995<br>VISITEUR………… 996	 <br>→ Q1004<br>→ Q1005
Q1002	Juste avant d'habiter dans cette maison/cet appartement, viviez-vous à Mbao, Guédiawaye, Pikine, Dakar, Mbour, Kaolack, ou ailleurs?<br><br>SI LA REPONSE EST LE NOM D'UNE REGION, DEMANDER S'IL S'AGIT D'UNE ZONE RURALE OU D'UNE ZONE URBAINE. SI LA REPONSE EST KAOLACK OU MBOUR, ENCERCLER LES CODES '06' OU '05' SI SEULEMENT IL S'AGIT DES COMMUNES CORRESPONDANTES.	MBAO………… 01<br>GUEDIAWAYE………… 02<br>PIKINE………… 03<br>DAKAR………… 04<br>MBOUR………… 05<br>KAOLACK………… 06<br>AUTRE VILLE………… 07<br>ZONE RURALE………… 08<br>ÉTRANGER………… 09	(01–06) → Q1004<br><br><br><br><br><br><br><br>→ Q1004
Q1003	Quel est le nom et l'emplacement de ce lieu?	NOM VILLE/VILLAGE ________<br><br>NOM DEPARTEMENT ________ [ ][ ]<br>A CODIFIER AU BUREAU<br>NOM REGION ________ [ ][ ]<br>A CODIFIER AU BUREAU	
Q1004	Depuis que vous êtes venue dans cette communauté, avez-vous vécu ailleurs pendant 6 mois ou plus?	OUI………… 1<br>NON………… 2	
Q1005	Au cours des 12 derniers mois, vous est-il arrivé d'aller dans une autre ville du Sénégal pour visiter vos parents ou amis?	OUI………… 1<br>NON………… 2	 → Q1015
Q1006	A quelle ville êtes-vous allé le plus souvent pour rendre visite à vos parents ou amis au cours des 12 derniers mois?	NOM VILLE ________<br><br>NOM DEPARTEMENT ________ [ ][ ]<br>A CODIFIER AU BUREAU<br>NOM REGION ________ [ ][ ]<br>A CODIFIER AU BUREAU	
Q1007	Au cours des 12 derniers mois, à quelle fréquence avez-vous visité [LIEU MENTIONNE A Q1006]?	PAR SEMAINE………… 1 [ ][ ]<br>PAR.MOIS………… 2 [ ][ ]<br>PAR.AN………… 3 [ ][ ]	
Q1008	Combien de temps restez-vous habituellement lorsque vous allez là-bas ?	MINUTES………… 1 [ ][ ]<br>HEURES………… 2 [ ][ ]<br>JOURS………… 3 [ ][ ]<br>SEMAINES………… 4 [ ][ ]<br>MOIS………… 5 [ ][ ]	
Q1009	Au cours de 12 derniers mois, combien de temps avez- vous passé à [NOM ENDROIT A Q1006] ?	HEURES………… 1 [ ][ ]<br>JOURS………… 2 [ ][ ]<br>SEMAINES………… 3 [ ][ ]<br>MOIS………… 4 [ ][ ]	
Q1010	Au cours de vos visites, vous arrive-t-il de discuter de Contraception ou de l'espacement des naissances avec quelqu'un là-bas?	OUI………… 1<br>NON………… 2	

Q1011	Au cours de vos visites, vous arrive-t-il de recourir à des services de PF ou d'espacement des naissances là-bas?	OUI .... 1<br>NON 2	
Q1012	Arrive-t-il que vos amis ou votre famille de cette ville viennent vous rendre visite?	OUI .... 1<br>NON 2	→ Q1015
Q1013	Vous arrive-t-il de parler de PF/Contraception lorsqu'ils vous rendent visite?	OUI .... 1<br>NON 2	
Q1014	Au cours de leurs visites ici, arrive-t-il que vos visteurs recourent à des services de PF ou d'espacement des naissances?	OUI .... 1<br>NON .... 2<br>NE SAIT PAS 8	
Q1015	Au cours des <u>12 derniers mois</u>, vous est-il arrivé d'aller dans une zone rurale du Sénégal pour visiter vos parents ou amis?	OUI .... 1<br>NON 2	→ SECT. 11
Q1016	A quelle zone eurale êtes-vous allé <u>le plus souvent</u> pour rendre visite à vos parents ou amis au cours des <u>12 derniersmois</u>?	NOM VILLAGE______________________________<br>NOM DEPARTEMENT __________________ [ ][ ]<br>A CODIFIER AU BUREAU<br>NOM REGION ________________________ [ ][ ]<br>A CODIFIER AU BUREAU	
Q1017	Au cours des <u>12 derniers mois</u>, à quelle fréquence avez-vous visité [LIEU MENTIONNE A Q1016]?	PAR SEMAINE.......... 1<br>PAR.MOIS.......... 2<br>PAR.AN.......... 3	
Q1018	Combien de temps restez-vous <u>habituellement</u> lorsque vous allez là-bas ?	MINUTES.......... 1<br>HEURES.......... 2<br>JOURS .... 3<br>SEMAINES.......... 4<br>MOIS.......... 5	
Q1019	Au cours des <u>12 derniersmois</u>, combien de temps avez-vous passé à [NOM VILLAGE] ?	HEURES.......... 1<br>JOURS .... 2<br>SEMAINES.......... 3<br>MOIS.......... 4	
Q1020	Au cours de vos visites, vous arrive-t-il de discuter de Contraception ou de l'espacement des naissances avec quelqu'un là-bas?	OUI .... 1<br>NON 2	
Q1021	Au cours de vos visites, vous arrive-t-il de recourir à des services de PF ou d'espacement des naissances là-bas?	OUI .... 1<br>NON 2	
Q1022	Arrive-t-il que vos amis ou votre famille de cette zone rurale viennent vous rendre visite?	OUI .... 1<br>NON 2	→ SECT. 11
Q1023	Vous arrive-t-il de parler de PF/Contraception lorsqu'ils vous rendent visite?	OUI .... 1<br>NON 2	
Q1024	Au cours de leurs visites ici, arrive-t-il que vos visteurs recourent à des services de PF ou d'espacement des naissances?	OUI .... 1<br>NON .... 2<br>NE SAIT PAS 8	
	ENREGISTREZ L'HEURE:	HEURE.......... [ ][ ]<br>MINUTES.......... [ ][ ]	

OBSERVATIONS DE L'ENQUÊTRICE

À REMPLIR APRÈS AVOIR TERMINÉ L'INTERVIEW

COMMENTAIRES SUR L'ENQUÊTÉE :

COMMENTAIRES SUR DES QUESTIONS PARTICULIÈRES :

OBSERVATIONS DU CHEF D'ÉQUIPE

NOM DU CHEF D'ÉQUIPE : ____________________ DATE : ____________________

OBSERVATIONS DU SUPERVISEUR

NOM DU SUPERVISEUR ______________________________ DATE : ______________

SITE	GRAPPE	N° MEN	N°LIGNE

SECTION 11: CONTACTS TELEPHONIQUES

	Questions et filtres	Codes
Q1100	PRENOMS DU REPONDANT : ______________________________	
Q1101	NOM DU REPONDANT :______________________________	
Q1103	ADDRESSE/DESCRIPTIONDU MENAGE :______________________________	
Comme je l'ai mentionné plus tôt, nous aimerions procéder à un suivi avec vous dans 2 ans et puis dans 2 autres années encore pour voir ce qui, si quelque chose, a changé concernant vos besoins en matière de santé et d'informations. Pour cette raison, je vais vous demander les contacts de gens qui pourront nous dire où vous êtes dans 2 ans et dans 4 ans au cas où vous aurez déménagé. Souvenez-vous, vous êtes libre de sauter les questions auxquelles vous êtes mal à l'aise de fournir des réponses. Ces informations seront conservées séparément de vos réponses dans un meuble verrouillé dans un bureau sûr auquel seul le chercheur principal a accès.		
Q1104	Quels sont les autres noms qu'on vous attribue généralement dans votre communauté ?	______________________<br>______________________
Q1105	Avez-vous un ou des numéros de téléphone portable et/ou fixe **personnels** sur lesquels nous pourrons vous joindre quand nous voudrions revenir vous rendre visite ?	OUI..........................1<br>NON ....2 → **Q1108**
Q1106	S'il vous plaît, pouvez-vous me communiquer votre numéro de téléphone portable principal, de téléphone portable secondaire et de votre téléphone fixe?	NUMERO PORTABLE PRINCIPAL<br>|_|_|_|_|_|_|_|_|_| NUMERO PORTABLE SECONDAIRE<br>|_|_|_|_|_|_|_|_|_| NUMERO FIXE<br>|_|_|_|_|_|_|_|_|_|
Q1108	Où est ce qu'on peut vous trouver dans 2 ans?	NOM ENDROIT______________________ 1<br>MÊME MENAGE... 2<br>UN AUTRE MENAGE...................................... 3<br>NE SAIT PAS.. 8
Q1109	SI REPONDANT N'EST PAS LE CHEF DE MENAGE<br>Quelle est le nom et le numéro de téléphone du CM?	NOM ______________________________<br>NUMERO DE PORTABLE :<br>|_|_|_|_|_|_|_|_|_|
Q1110	S'il vous plaît, pouvez-vous me communiquer le nom et le numéro de téléphone portable de la personne qui est la plus proche de vous dans votre ménage ?<br>Quel est votre lien de parenté avec cette personne?	NOM ______________________________<br>LIEN PARENTE AVEC LE CM:________________<br>NUMERO DE PORTABLE :<br>|_|_|_|_|_|_|_|_|_| LIEN AVEC L'ENQUETEE_____________________
Q1111	S'il vous plaît, pouvez-vous me communiquer le nom et le numéro de téléphone d'une personne qui vous est proche/intime en dehors de votre ménage?<br>Quel est votre lien de parenté avec cette personne?	NOM 1______________________________<br>LIEN PARENTE AVEC LE CM:________________<br>NUMERO DE PORTABLE :<br>|_|_|_|_|_|_|_|_|_| LIEN AVEC L'ENQUETEE_____________________
Q1112	S'il vous plaît, pouvez-vous me communiquer le nom et le numéro de téléphone d'une autre personne qui vous est proche/intime en dehors de votre ménage?<br>Quel est votre lien de parenté avec cette personne?	NOM 2 ______________________________<br>LIEN PARENTE AVEC LE CM:________________<br>NUMERO DE PORTABLE :<br>|_|_|_|_|_|_|_|_|_| LIEN AVEC L'ENQUETEE_____________________
Q1113	En dehors d'ici (ce ménage), quelle autre ménage fréquentez-vous souvent?	ADREESE______________________________<br>PERSONNE DE CONTACT ____________________<br>N° TELEPHONE PERSONNE DE CONTACT :<br>|_|_|_|_|_|_|_|_|_|

S		GR		MEN		LIG

OBSERVATIONS ET COMMENTAIRES PARTICULIERS SUR LES CONTACTS DE L'ENQUETEE
