# Supplementary material for: Influence of integrated services on postpartum family planning use: a cross-sectional survey from urban Senegal
Source: BMC Public Health. 2013 Aug 14;13:752. doi: 10.1186/1471-2458-13-752 (PMC3846684; doi:10.1186/1471-2458-13-752)
Supplement: Additional file 2 — Questionnaire Cliente A La Sortie Des Structures Sanitaires. [file 1471-2458-13-752-S2.pdf]

MESURE APPRENTISSAGE ET ÉVALUATION (MLE) DE L'INITIATIVE SÉNÉGALAISE DE SANTE URBAINE (ISSU)
ENQUÊTE DE BASE (2011)

QUESTIONNAIRE CLIENTE A LA SORTIE DES STRUCTURES SANITAIRES

NUMÉRO QUESTIONNAIRE…. [ ][ ] [ ][ ][ ] [ ][ ]
SITE TYPE NUM STRUCT NUM REP

SITE (DAKAR=1, GUEDIAWAYE=2, PIKINE=3, MBAO=4, MBOUR=5, KAOLACK=6)…………………	SITE……………………………[ ]
DISTRICT SANITAIRE…………………………………………………………………………	DS……………………………[ ][ ]
COMMUNE / COMMUNE D'ARRONDISSEMENT ________________________	C / C A……………………[ ][ ]
TYPE DE STRUCTURE…………………………………………………………………………	TYPE**…………………………[ ]
NOM ET CODE DE LA STRUCTURE SANITAIRE________________________	NUM STRUCTURE….[ ][ ][ ]
NUMÉRO DE LA RÉPONDANTE (CODAGE BUREAU)……………………………………	NUM RÉPONDANTE…….[ ][ ]

TYPE DE STRUCTURE DE SANTÉ
SECTEUR PUBLIC
HÔPITAL ……………………………………………………11
CENTRE DE SANTE ………………………………………12
POSTE DE SANTE…………………………………………13
CASE DE SANTE……………………………………………14
AUTRE PUBLIC ______________________________16
(PRÉCISER)
SECTEUR PRIVE FORMEL
CLINIQUE PRIVÉE……………………………………………21
CABINET PRIVE ……………………………………………22
DISPENSAIRE CONFESSIONNEL…………………………23
AUTRE MÉDICAL PRIVE______________________26
(PRÉCISER)
ONG
CLINIQUE D'ONG……………………………………………31
AUTRE STRUCTURE D'ONG____________________36
(PRÉCISER)

AUTRE_____________________________________96
(PRÉCISER)

CODE TYPE**
HAUT VOLUME PUBLIC…………………………………1
HAUT VOLUME PRIVÉ…………………………………2

	FRANÇAIS	WOLOF	AUTRE
LANGUE DE L'ENTRETIEN………..	1	2	6
SI AUTRE (PRÉCISER)________________			
LANGUE MATERNELLE DE L'ENQUÊTEE…………………….	1	2	6
SI AUTRE (PRÉCISER)________________			

	OUI	NON
INTERPRÈTE?	1	2

VISITES ET RÉSULTATS DE L'ENQUÊTRICE

**N° visite**	**1**	**2**	**3**	**VISITE FINALE**
**DATE**	JOUR/MOIS/ANNÉE [___/____/_**11**_]	JOUR/MOIS/ANNÉE [___/____/_**11**_]	JOUR/MOIS/ANNÉE [___/____/_**11**_]	JOUR [___|___] MOIS [___|___] ANNÉE [_**2**_|_**0**_|_**1**_|_**1**_]
**NOM ENQUÊTEUR**	____________	____________	____________	____________
**CODE ENQUÊTEUR**	[ ][ ]	[ ][ ]	[ ][ ]	[ ][ ]
**RÉSULTAT***	[ ]	[ ]	[ ]	[ ]
**PROCHAINE VISITE : DATE:**	[___/____/11_]	[___/____/11_]	[___/____/11_]	TOTAL NO. DE VISITES
**HORAIRE :**	[ ][ ] H H [ ][ ] M M	[ ][ ] H H [ ][ ] M M	[ ][ ] H H [ ][ ] M M	[ ]

***CODES RÉSULTATS :**
1. REMPLI 3. REFUS
2. PARTIELLEMENT REMPLI 6. AUTRE ______________________
(PRÉCISER)

**SUPERVISEUR**	**CONTROLE DE BUREAU**	**SAISI PAR**
NOM ____________________	NOM ____________________	NOM ____________________
CODE……………………………[ ][ ]	CODE……………………………[ ][ ]	CODE……………………………[ ][ ]
DATE …………………[__|__] [__|__][__|__] JJ MM AA	DATE …………………[__|__] [__|__][__|__] JJ MM AA	DATE …………………[__|__] [__|__][__|__] JJ MM AA

Lettre d’information de la femme pour l’interview à la sortie des points de prestations de services

Nom et prénoms de la femme : ____

Madame,

Mon nom est_______________________ et je travaille comme enquêteur/chef d’équipe dans une recherche commanditée par Intrahealth pour le compte du Ministère de la Santé et de la Prévention et réalisée par le CRDH (Centre de Recherche pour le Développement Humain).

Il vous est proposé de participer à une recherche sur la santé maternelle et infantile qui vise à améliorer la qualité de vie des populations urbaines des villes sénégalaises à travers l’augmentation de l’accès et l’utilisation des services de santé de la reproduction. Si vous acceptez de participer, vous ferez partie des 13 000 femmes qui seront interrogées dans le cadre de cette recherche. Les informations que vous allez nous fournir seront très utiles au gouvernement pour planifier les politiques de santé et répondre aux besoins non satisfaits en santé de la reproduction. Vous avez été choisie au hasard parmi les clientes dans ce service.

Vous serez interviewé par une enquêtrice/enquêteur. Des informations sur l’accès et l’utilisation des services de santé, sur la santé maternelle et infantile, sur votre satisfaction des services reçus dans ce service et sur votre niveau de vie vous seront demandées.

L'entretien prend généralement entre 15 et 30 minutes. Nous avons pris les dispositions idoines pour que toute information que vous nous fournirez reste strictement secrète. L'entretien va être confidentiel et se tiendra en privé. Si les conditions pour que l’entretien se tienne en privé ne sont pas réunies, nous allons arrêter l’interview jusqu’à ce que celles-ci le soient. L'exploitation de l'information sera et restera anonyme.

Vous ne tirerez pas d'avantages directs de cette enquête, mais les informations que vous allez nous fournir aideront le Gouvernement à planifier les actions futures dans le domaine de la santé de la reproduction.

Il n’y a pas d’inconvénients à participer à cette enquête, sauf le temps que vous nous accorder pour répondre aux questions posées. Aucun prélèvement ou traitement n’est envisagé dans le cadre de cette enquête. La participation à cette enquête est volontaire; s'il y a une question à laquelle vous ne voulez pas répondre, faites-le savoir et l'enquêteur passera à la question suivante. Vous pouvez également renoncer à l'entretien à tout moment. Nous espérons cependant que vous participerez à cette enquête car votre opinion est particulièrement importante pour nous.

Si vous acceptez de participer, vous devez signer ce document pour signifier que vous avez reçu l’information et marquer votre accord. Une copie du document signé vous sera remise.

Vous pouvez contacter le Directeur du Centre de Recherche pour le Développement Humain, Téléphone : 33 832 63 79, à tout moment si vous avez des questions au sujet de l’enquête.

Vous pouvez contacter Dr. Samba Cor Sarr, Direction de la Santé, 1 Rue Aimé Césaire, 2ème étage Dakar Fann, BP 4024, Tél : 33 869 43 13, à tout moment si vous avez des questions au sujet de vos droits en tant que participant à l’enquête.

Votre participation à cette enquête est volontaire, ni votre entourage ni vous-même, ne serez pénalisés, ni ne perdrez aucun de vos droits si vous décidez d’arrêter votre participation.

Acceptez-vous de participer à l’enquête?

OUI……………… ☐ NON……… ☐ → **FIN INTERVIEW**

↓

(SIGNATURE FICHE DE CONSENTEMENT)

**ÉLIGIBILITÉ DES PARTICIPANT/QUESTIONS DE SÉLECTION**			
**No.**	**Questions**	**/ Codification**	**Saut**
**Q1.**	*ENREGISTREZ L’HEURE DE DÉBUT*	[__|__] : [__|__]	
**Q2.**	Avez-vous vu un agent de santé aujourd’hui pour des soins?	OUI……………………………………..1<br>NON…………………………………….2 →	**FIN DE L’ENTRETIEN**
**Q3.**	Quel âge aviez-vous à votre dernier anniversaire ?	AGE EN ANNÉES RÉVOLUES…….[__|__]	**Arrêter, si l’enquêtée a moins de 15 ans ou plus de 49 ans**

**INFORMATIONS RELATIVES A LA VISITE**			
A présent, je voudrais que nous échangions sur les services de santé de la reproduction pour lesquels vous êtes venue dans ces installations aujourd'hui.			
**Q4.**	Pour quel type de service êtes-vous **principalement** venue ici aujourd'hui ?	PLANIFICATION FAMILIALE-------------------------------01<br><br>CONSULTATION PRÉNATALE--------------------------02<br>SERVICES D'ACCOUCHEMENT -----------------------03<br>CONSULTATION POSTNATALE------------------------04<br>SOINS APRÈS AVORTEMENT---------------------------05<br>SUIVI DE LA CROISSANCE DES ENFANTS--------- 06<br>VACCINATION DE L'ENFANT----------------------------07<br>CONSULTATION IST--08<br>CONSULTATION VIH/ SIDA ----------------------------- 09<br>SERVICES CURATIFS ----------------------------------- 10<br>CONSEILS DÉPISTAGE VOLONTAIRE--------------- 11<br>AUTRE ___________________________________ 96<br>(PRÉCISER)	02–96 → **Q19**

**Q5.**	Avant cette visite, qu'aviez-vous fait /quelles méthodes aviez-vous utilisées pour éviter de tomber enceinte?<br><br>**RÉPONSES MULTIPLES POSSIBLES. ENCERCLER TOUT CE QUI EST MENTIONNE.**	PILULES… ….………………………………..….A<br>PRESERVATIF MASCULIN ……….……….....… B<br>PRESERVATIF FEMININ …. . . ……………......…C<br>DIU………... . . . . …. ………………… D<br>INJECTABLES.. . . . . ……………….…E<br>IMPLANT... . . . . ………………….F<br>MÉTHODES NATURELLES (MÉTHODE DU RYTHME /ABSTINENCE PÉRIODIQUE / RETRAIT)……………………………………….G<br>ALLAITEMENT AU SEIN /MAMA…………......….H<br>STÉRILISATION FÉMININE ....I<br>STÉRILISATION MASCULINE............................J<br>CONTRACEPTION D'URGENCE……...………..K<br>AUTRE ________________________________X<br>(PRÉCISER)<br>RIEN……………………………………………..….Y	Y → **Q13**
**Q6.**	Avez-vous utilisé une méthode de PF la dernière fois que vous avez eu un rapport sexuel?	OUI.................................. ……………..1<br>NON 2	
**Q7**	Avant votre visite d'aujourd'hui, quelle était la dernière fois que vous avez utilisé une méthode de PF pour éviter une grossesse ?	UTILISATRICE ACTUELLE DE MÉTHODE………..1<br>AU COURS DE LA SEMAINE DERNIÈRE…………2<br>IL Y A 1-3 SEMAINES………………………………..3<br><br>IL Y A 1-3 MOIS……………………………………….4<br>IL Y A 4-6 MOIS……………………………………….5<br>IL Y A 7 MOIS – 11 MOIS…………………….........6<br>IL Y A D'UN AN OU PLUS ………………………….7	4–7 → Q9
**Q8.**	Quelle(s) méthode(s) aviez-vous utilisé à ce moment?<br><br>**RÉPONSES MULTIPLES POSSIBLES. ENCERCLER TOUT CE QUI EST MENTIONNÉ.**	PILULES… ….………………………………..….A<br>PRESERVATIF MASCULIN ……….……….......B<br>PRESERVATIF FEMININ …. . . ……………......…C<br>DIU………... . . . . …. …………………D<br>INJECTABLES.. . . . . ……………….…E<br>IMPLANT... . . . . ………………….F<br>MÉTHODES NATURELLES (MÉTHODE DU RYTHME /ABSTINENCE PÉRIODIQUE / RETRAIT)……………………………………..G<br>ALLAITEMENT AU SEIN /MAMA…………......….H<br>STÉRILISATION FÉMININE ....I<br>STÉRILISATION MASCULINE............................J<br>CONTRACEPTION D'URGENCE……...………..K<br>AUTRE ________________________________X<br>(PRÉCISER)	

Q9.	Quelle était votre principal objectif en venant aujourd'hui pour une visite de planification familiale?<br><br>**NOTER UNE SEULE RÉPONSE**<br><br>**SI L'ENQUÊTÉE NE MENTIONNE PAS DE FAÇON EXPLICITE UNE DES OPTIONS LISTÉES, POURSUIVRE L'INTERVIEW EN ÉNUMÉRANT CES OPTIONS ET EN LUI DEMANDANT CELLE QUI CORRESPOND AU MIEUX AUX RAISONS DE SA VISITE AUJOURD'HUI.**<br>**N'ENCERCLER QU'UNE SEULE RÉPONSE**.	RÉAPPROVISIONNEMENT CONTRACEPTIFS….01<br>CHANGER POUR UNE AUTRE MÉTHODE………02<br>ARRÊTER LA CONTRACEPTION…………………..03<br>SUIVI DE ROUTINE ………………………………….04<br>SUIVI A LA SUITE DE PROBLÈMES………………05<br>RECOMMENCER LA PF……………………………06 →<br>AUTRE ________________________________96<br>(PRÉCISER)	 <br><br><br><br>**Q13**

**ANCIENNES UTILISATRICES**					
**Q11.**	Au cours de votre consultation d'aujourd'hui, l'agent vous a –t-il …..	**OUI**	**NON**	**NSP/ NE SE SOUVIENT PAS**	**N A**
	a. Demandé le motif de votre visite?	1	2	8	7
	b. Posé des questions sur les difficultés que vous auriez avec la méthode actuelle avant cette visite?	1	2	8	7
	c. Suggéré une quelconque action pour surmonter ce problème ?	1	2	8	7
	d. Posé des questions sur votre but/objectif en termes de procréation?	1	2	8	7
	e. Donné des informations relatives aux différentes méthodes de la PF?	1	2	8	7
	f. Interrogé sur votre préférence pour une autre méthode de PF	1	2	8	7
	g. Parlé des possibles effets secondaires de l'actuelle méthode que vous utilisez?	1	2	8	7
	h. Parlé de ce que vous pouvez faire en cas de problèmes avec la méthode?	1	2	8	7
	i. Donné un rendez-vous pour le suivi?	1	2	8	7
**Q12.**	Quelles ont été les conclusions de la visite? Avez-vous décidé de continuer avec la même méthode, d'arrêter ou changer de méthodes ?	CONTINUER AVEC LA MÊME MÉTHODE …………..1 →<br>CHANGER DE MÉTHODE... ………………………..… 2 →<br>ARRÊTER LA MÉTHODE (SUITE À DES PROBLÈMES)……………………………….……..…3<br>ARRÊTER LA MÉTHODE (AUCUNE RAISON ÉVOQUÉE – PAS DE PROBLÈMES)…………..…...4<br>AUTRES (PRÉCISER)________________________6			**Q16**<br>**Q13e**<br><br>**Q35**

**NOUVELLE UTILISATRICE ET/OU N'UTILISE PLUS AU MOMENT DE LA CONSULTATION**					
**Q13.**	Au cours de votre consultation d'aujourd'hui, l'agent vous a –t-il …..	**OUI**	**NON**	**NSP/ NE SE SOUVIENT PAS**	**N A**
	a. Demandé le motif de votre visite?	1	2	8	7
	b. Posé des questions sur votre but/objectif en termes de procréation?	1	2	8	7
	c. Donné des informations relatives aux différentes méthodes de PF?	1	2	8	7
	d. Interrogé sur votre préférence pour une méthode de PF	1	2	8	7
	e. Aidé à choisir une méthode de PF ?	1	2	8	7
	f. Expliqué comment utiliser la méthode choisie ?	1	2	8	7
	g. Parlé des possibles effets secondaires de cette méthode?	1	2	8	7
	h. Expliqué ce qu'il faut faire en cas de problème avec cette méthode?	1	2	8	7
	i. Donné un rendez-vous pour le suivi?	1	2	8	7
**Q14.**	Avant de venir dans cette structure aujourd'hui, saviez-vous quelle(s) méthode(s) de PF vous voudriez utiliser ?	OUI…………………………………………………1<br>NON……………………………………………..2 →			 **Q16**

**Q15.**	Quelle(s) était (aient) cette (ces) méthode(s)?<br><br>**RÉPONSES MULTIPLES POSSIBLES. ENCERCLER TOUT CE QUI EST MENTIONNE.**	PILULES… ….………………………………..…A<br>PRESERVATIF MASCULIN ……………….…...... B<br>PRESERVATIF FEMININ …. . . …………..……C<br>DIU………... . . . . …. ………………… D<br>INJECTABLES.. . . . . …………………E<br>IMPLANT... . . . . ……………….....F<br>MÉTHODES NATURELLES (MÉTHODE DU RYTHME /ABSTINENCE PÉRIODIQUE / RETRAIT)……………………………………..G<br>ALLAITEMENT AU SEIN /MAMA…………....…..H<br>STÉRILISATION FÉMININE ....I<br>STÉRILISATION MASCULINE...........................J<br>CONTRACEPTION D'URGENCE……...……..….K<br>AUTRE ______________________________X<br>(PRÉCISER)	
**Q16.**	Avez-vous reçu l'une de ces méthodes contraceptives aujourd'hui?	OUI……………………………………………….1<br>NON……………………………………………….2	OUI → **Q18**
**Q17.**	Avez-vous reçu une référence, ou une prescription pour une méthode de planification familiale aujourd'hui?	OUI, RÉFÉRENCE REÇUE ……………………..1<br>OUI, PRESCRIPTION REÇUE …………………2<br>NON, N'A RIEN REÇU……………………………3<br>UTILISE DÉJÀ…………………………………….4	3 → **Q35**<br>4 → **Q35**
**Q18.**	(Pour) Quelle(s) méthode(s)?<br><br>**RÉPONSES MULTIPLES POSSIBLES. ENCERCLER TOUT CE QUI EST MENTIONNE.**	PILULES… ….………………………………..…A<br>PRESERVATIF MASCULIN ……………….…...... B<br>PRESERVATIF FEMININ …. . . …………..……C<br>DIU………... . . . . …. ………………… D<br>INJECTABLES.. . . . . …………………E<br>IMPLANT... . . . . ………………….F<br>MÉTHODES NATURELLES (MÉTHODE DU RYTHME /ABSTINENCE PÉRIODIQUE / RETRAIT)………………………………………G<br>ALLAITEMENT AU SEIN /MAMA……………....…H<br>STÉRILISATION FÉMININE ....I<br>STÉRILISATION MASCULINE.........................J<br>CONTRACEPTION D'URGENCE……...……..….K<br>AUTRE ______________________________X<br>(PRÉCISER)	A–X → **Q35**

UTILISATRICES POTENTIELLES D'INTÉGRATION

**Q19.**	Y aurait-t-il d'autres préoccupations liées à la santé sur lesquelles vous aviez souhaité en savoir davantage aujourd'hui et qui n'auraient pas été abordées ?	OUI. . . . .…..…. . . . . . .1<br>NON. . . . .…..…... 2	NON → **Q21**
**Q20.**	A quoi étaient liées ces préoccupations sanitaires ?<br><br>**NE PAS LIRE LES ÉLÉMENTS DE LA LISTE**<br><br>**RÉPONSES MULTIPLES POSSIBLES. ENCERCLER TOUT CE QUI EST MENTIONNE.**	CONSULTATION PRÉNATALE……………….A<br>SERVICES ACCOUCHEMENTS.……………..B<br>SOINS POST NATALS…………………………C<br>SUIVI DE LA CROISSANCE DES ENFANTS..D<br>CONSULTATION IST. . . . ….. . ……………. E<br>CONSULTATION VIH.. . . . . ……………….…..F<br>CONSULTATION GÉNÉRALE. . ……………...G<br>SERVICES DE NUTRITION……………..……..H<br>VACCINATION ENFANTS…………………..…..I<br>SOINS APRÈS-AVORTEMENT……….……….J<br>CONSEIL DÉPISTAGE VOLONTAIRE………..K<br>PLANIFICATION FAMILIALE.…….……………..L<br>AUTRES SERVICES__________________X<br>(PRÉCISER)	

**Q21.**	**VÉRIFIER Q4:**<br><br>SI N'IMPORTE QUEL TYPE DE VISITE, INCLUANT :<br><br>SUIVI DE LA CROISSANCE DES ENFANTS **(Q4=06)**, OU VACCINATION ENFANTS **(Q4=07)**, OU CONSULTATION IST **(Q4=08)**, OU CONSULTATION VIH/SIDA **(Q4=09)**, OU SERVICES CURATIFS **(Q4=10)**, CDV **(Q4=11)** OU AUTRES **(Q4=96)** ☐ ↓	VISITE PRÉNATALE OU ACCOUCHEMENT **(Q4=2 OU 3)** ☐<br><br>SOINS POST NATALS OU SOINS APRÈS AVORTEMENT **(Q4=04 OU 05)** ☐	→ **Q26**<br><br>→ **Q23**

**Q22.**	Êtes-vous actuellement enceinte?	OUI…………………………….1<br>NON……………………..………2<br>INCERTAINE………………….8	→ **Q26** (OUI)
**Q23.**	Faites –vous (prenez-vous) présentement quelque chose pour éviter de tomber enceinte ?	OUI . . . .….1<br>NON . . . .…..2	→ **Q25** (OUI)

**Q24.**	Pourquoi n'utilisez-vous pas une méthode de planification familial/espacement des naissances pour éviter de tomber enceinte ?<br><br>**RÉPONSES MULTIPLES POSSIBLES.**<br><br>**NE PAS LIRE LES RÉPONSES, ENCERCLER TOUT CE QUI EST MENTIONNE.**	**RAISONS LIEES A LA FECONDITE**<br>PEU/ PAS DE RAPPORTS SEXUELS………..………..A<br>MARI ABSENT……………………..…………..……....B<br>MENOPAUSE/HYSTÉRECTOMIE……………...………C<br>ALLAITEMENT MATERNEL…………………..…..……D<br>NE PEUT PAS AVOIR D' ENFANT……..………..………E<br>VEUT AUTANT D'ENFANTS QUE POSSIBLE ……………F<br>VEUT PRENDRE /TENTE DE PRENDRE UNE GROSSESSE…………………………………..…………...G<br>AMÉNORRHÉE POST-PARTUM……………..…….………H<br>**REFUS D'UTILISATION**<br>L'ENQUÊTÉE S'Y OPPOSE…..………………..……………..….I<br>LE PARTENAIRE S'Y OPPOSE . ……………………………J<br>LES AUTRES S'YOPPOSENT ………………………………K<br>INTERDICTION RELIGIEUSE …………………………..…….L<br>HONTE, CRAINTE DE STIGMATISATION…………………M<br>**L'IGNORANCE**<br>NE CONNAÎT AUCUNE MÉTHODE. . . . .…….N<br>NE SAIT PAS COMMENT UTILISER UNE MÉTHODE.. . ..O<br>NE CONNAÎT AUCUNE SOURCE. . . . .……………P<br>**RAISONS LIÉES AUX METHODES**<br>PROBLEMES DE SANTE……... ….. . . . .………….Q<br>PEUR DES EFFETS SÉCONDAIRES. . . . . . ……………….R<br>MANQUE D'ACCESS / TROP DISTANT. . . ……………….S<br>COÛT TROP ÉLEVÉ. . . . .…………..…………...T<br>PEU COMMODE À L'UTILISATION . . . .………..U<br>N'AIME PAS LES MÉTHODES EXISTANTES…………….V<br>MAUVAISE EXPÉRIENCE AVEC LES MÉTHODES EXISTANTES……...……………………………………...W<br>**FATALISTE**<br>TOUT DÉPEND DE DIEU……..………………………....Y<br>AUTRE ________________________________X<br>NE SAIT PAS. . . . . ……... ………………..Z	→ **Q26**
**Q25.**	Quelle méthode utilisez-vous **actuellement**?<br><br>**RÉPONSES MULTIPLES POSSIBLES.**<br>**ENCERCLER TOUT CE QUI EST MENTIONNE.**	PILULES… …...……………………………..….A<br>PRESERVATIF MASCULIN ………………...…. B<br>PRESERVATIF FEMININ …. . . ………...........C<br>DIU………... . . . . …. ………………… D<br>INJECTABLES.. . . . .………..……….E<br>IMPLANT... . . . .…………………..F<br>MÉTHODES NATURELLES (MÉTHODE DU RYTHME /ABSTINENCE PÉRIODIQUE / RETRAIT)………………………………………G<br>ALLAITEMENT AU SEIN /MAMA……………..….H<br>STÉRILISATION FÉMININE ....I<br>STÉRILISATION MASCULINE..........................J<br>CONTRACEPTION D'URGENCE……...……..….K<br>AUTRE ____________________________X<br>(PRÉCISER)	
**Q26.**	Au cours de cette visite, avez-vous reçu ou vu une information sur la PF ?	OUI . . . .….. 1<br>NON. . . . . ……..2	→ **Q29** (NON)
**Q27.**	Comment avez-vous eu l'information?<br><br>**RÉPONSES MULTIPLES POSSIBLES.**<br>**ENCERCLER TOUT CE QUI EST MENTIONNE.**	PAR LE BIAIS DE L'AGENT DE PF………………………..…..A<br>PAR LE BIAIS D'UNE VIDÉO……………………………………B<br>EN PARTICIPANT À UN GROUPE DE DISCUSSIONS………………………………….…....…………..C<br>GRACE À DES DOCUMENTS ÉCRITS (AFFICHES, DÉPLIANTS, ETC.)…………………………….……………D<br>EN POSANT DES QUESTIONS SUR LA PF………………...E<br>AUTRE ________________________________X	

**Q28.**	De quels sujets a-t-on parlé selon les informations que vous avez reçues ou vues ?<br><br>**RÉPONSES MULTIPLES POSSIBLES.**<br>**NE PAS LIRE LES RÉPONSES, ENCERCLER TOUT CE QUI EST MENTIONNÉ.**	MÉTHODES PF..A<br>IMPLICATION DES HOMMES DANS LA PF........................B<br>BIENFAITS DE L'ESPACEMENT DES GROSSESSES........C<br>ALLAITEMENT AU SEIN / MAMA...D<br>BUTS/OBJECTIFS DE LA SR..E<br>EFFETS SECONDAIRES DES MÉTHODES DE PF............F<br>AUTRE____________________________________X<br>PRÉCISER	
**Q29.**	Savez-vous si vous pouvez obtenir des méthodes ou services de PF dans cette structure ?	OUI, PEUT RECEVOIR PF ICI ……………1<br>NON, NE PEUT PAS RECEVOIR PF ICI…..2 →<br>NE SAIT PAS.………………………….……8	**Q32**
**Q30.**	Avez-vous reçu une méthode de planification familiale, une référence, ou une prescription pour l'espacement des naissances aujourd'hui?	OUI, MÉTHODE REÇUE. . . . . ……………………………1<br>OUI, RÉFÉRENCE REÇUE…………...................................2<br>OUI, PRESCRIPTION REÇUE……………………………….3<br>OUI, MÉTHODE ET RÉFÉRENCE/PRESCRIPTION REÇUE…4<br>NON, N'A RIEN REÇU POUR PF . . . …………………………..5 → **Q32**<br>UTILISE DÉJÀ ……………………….……………………….6 → **Q37**<br>FEMME ENCEINTE…………………………………………….7 → **Q33**	
**Q31.**	(Pour) quelle(s) méthode(s)<br><br>**RÉPONSES MULTIPLES POSSIBLES. ENCERCLER TOUT CE QUI EST MENTIONNE.**	PILULES… ….....…………………………..….A<br>PRESERVATIF MASCULIN …………………....... B<br>PRESERVATIF FEMININ …. . . …………..........C<br>DIU……….... . . . . …. ………………… D<br>INJECTABLES.. . . . . ……………….…E<br>IMPLANT... . . . . ….………………F<br>MÉTHODES NATURELLES (MÉTHODE DU RYTHME /ABSTINENCE PÉRIODIQUE / RETRAIT)………………………………………G<br>ALLAITEMENT AU SEIN /MAMA……………..….H<br>STÉRILISATION FÉMININE ....I<br>STÉRILISATION MASCULINE..........................J<br>CONTRACEPTION D'URGENCE……...……..…K<br>AUTRE ________________________________X<br>(PRÉCISER)	**Q37**
**Q32.**	Si un prestataire dans ce service vous avait offert une méthode de PF aujourd'hui, auriez-vous accepté ?	OUI . . . . ……………. 1<br>NON . . . . …………… 2	
**Q33.**	Seriez-vous intéressé à discuter de PF avec un prestataire au niveau de l'unité où vous avez reçu votre principal service aujourd'hui (MENTIONNÉ À Q4)?	OUI . . . . ………………. 1 →<br>NON . . . . ………………. 2<br>NE SAIT PAS . . . . ……………….. 8	**Q37**
**Q34.**	Pourquoi vous ne seriez pas intéressée à avoir cette discussion ?<br><br>**RÉPONSES MULTIPLES POSSIBLES. ENCERCLER TOUT CE QUI EST MENTIONNE.**	PAS LE BON MOMENT POUR DISCUTER………………….. .. . A<br>PAS LE BON MOMENT, ENFANTS PRÉSENTS……………...B<br>PAS A L'AISE AVEC L'AGENT…………………………………. C<br>N'A PAS LE TEMPS . . . ……………………………….………..D<br>VEUT PLUS D'ENFANTS. . ……………………………………....E<br>N'Y A JAMAIS PENSÉ. . . . .F<br>LE MARI S'Y OPPOSERAIT. …………………………………..…G<br>DÉSAPPROUVE LA PF……………………………………….…..H<br>PRÉSENTEMENT ENCEINTE…………………………………...I<br>INTERDICTION RELIGIEUSE…………………………………..…J<br>ALLAITEMENT AU SEIN…………………………………….…....K<br>AMÉNORRHÉE POST-PARTUM ………………………………….L<br>PEU/PAS DE RAPPORTS SEXUELS……………………….....M<br>ABSENCE DU MARI ……………………………………………….N<br>MÉNOPAUSE/HYSTÉRECTOMIE…………………………......O<br>NE PEUT PAS AVOIR D'ENFANT ……………………..…....P<br>PROBLÈMES DE SANTÉ………………………………………….Q<br>CRAINTE DES EFFETS SECONDAIRES………………………..R<br>TROP COUTEUX.……………..……………….......................S<br>PAS D'ACCÈS RÉGULIER AUX MÉTHODES…………………..T<br>PEU COMMODE A L'UTILISATION……………………………….U<br>N'AIME PAS LES MÉTHODES EXISTANTES…………………..V<br>MAUVAISE EXPÉRIENCE AVEC MÉTHODES EXISTANTES..W<br>MÉTHODES QUI L'INTÉRESSENT NE SONT DISPONIBLES..Y<br>AUTRE (PRÉCISER)________________________________X	**Q37**

INFORMATION CONCERNANT LA SATISFACTION DE LA CLIENTE			
Je voudrais commencer par vous poser des questions concernant les services que vous avez reçus aujourd'hui. S'il vous plaît, parlez-nous de l'agent qui vous a fourni la majorité des informations pendant votre visite. L'agent ne saura rien de vos réponses, par conséquent, soyez francs s'il vous plaît. Ces informations permettront d'améliorer les services de planification familiale.			
**N°**	**QUESTIONS**	***CODAGE***	***SAUT***
**Q35.**	En plus des services de PF que vous avez reçus aujourd'hui, avez-vous reçu d'autres services auprès du prestataire de santé ?	OUI . . . .1<br>NON . . . .…… 2	 **Q37**
**Q36.**	De quel(s) autre(s) service(s) avez-vous bénéficié (s)?<br><br>**NE PAS LIRE LA LISTE**<br><br>**RÉPONSES MULTIPLES POSSIBLES. ENCERCLER TOUT CE QUI EST MENTIONNE.**	CONSULTATION PRÉNATALE……………….A<br>SERVICES ACCOUCHEMENT………………..B<br>SOINS POST NATALS…………………………C<br>SUIVI DE LA CROISSANCE DES ENFANTS...D<br>CONSULTATION IST. . . . …... . ……..……….E<br>CONSULTATION VIH.. . . . . …….…………..F<br>SERVICES CURATIFS . . . . …...……...G<br>SERVICES DE NUTRITION………………..……..H<br>VACCINATION ENFANTS……………….……..I<br>SOINS APRÈS-AVORTEMENT……..………J<br>CONSEIL DÉPISTAGE VOLONTAIRE……….K<br>AUTRES SERVICES ___________________X<br>(PRÉCISER)	
**Q37.**	Environ combien de temps avez-vous attendu entre le moment où vous êtes arrivée au niveau de la formation sanitaire et le moment où vous avez vu un membre du personnel pour une consultation ?	<=15 MINUTES . . . . …….. . . . .1<br>16-30 MINUTES . . . . …….. . . . …. ……..2<br>31-45 MINUTES . . . . …….. . . . .…… 3<br>46-60 MINUTES . . . . …….. . . . . . ….…. 4<br>61 MINUTES- 90 MINUTES . . .. …. 5<br>91 MINUTES - 2H ……….. . . . ……... . . . . .6<br>PLUS DE 2H ……………. ……….…. 7	
**Q38.**	Estimez-vous que votre temps d'attente était raisonnable ou trop long?	PAS DE TEMPS D'ATTENTE…………….1<br>RAISONNABLE……………………………..2<br>TROP LONG………………………………..3<br>NE SAIT PAS. . . . .………..…8	
**Q39.**	Pendant votre entrevue avec le prestataire au cours de votre visite, pensez-vous que d'autres clientes pouvaient vous voir/apercevoir?	OUI . . . .….1<br>NON. . . . . ….2	
**Q40.**	Pendant votre entrevue avec le prestataire au cours de votre visite, pensez-vous que d'autres clientes pouvaient entendre ce que vous disiez?	OUI . . . . ….……….1<br>NON. . . . .……...2<br>NSP……………………………………………8	
**Q41.**	Étiez-vous à l'aise pour poser des questions pendant cette visite?	OUI . . . . ………….1<br>NON . . . . ……. ….2	
**Q42.**	Est-ce que le prestataire vous a demandé si vous aviez d'autres questions ?	OUI . . . . ….……….1<br>NON . . . .…….. 2	
**Q43.**	Est-ce que le prestataire a répondu à toutes vos questions?	OUI . . . . ….……….1<br>NON . . . .…….. 2	
**Q44.**	Avez-vous le sentiment que les informations vous concernant que vous avez partagées avec le prestataire seront gardées confidentielles?	OUI . . . . ….……….1<br>NON . . . .…….. 2<br>NE SAIT PAS . . . . ……………8	
**Q45.**	Pendant votre visite, comment avez-vous été traitée **par l'agent**? Diriez-vous que vous avez été « très bien traitée », « bien traitée », ou « pas très bien/mal traitée » ?	TRÈS BIEN………………………………….1<br>BIEN…………………………………………..2<br>PAS TRÈS BIEN/MAL……………………..3	
**Q46.**	Pendant votre visite, comment avez-vous été traitée **par les autres membres du personnel** ? Diriez-vous que vous avez été « très bien », « bien », ou « pas très bien/mal traitée » ?	TRÈS BIEN………………………………….1<br>BIEN…………………………………………..2<br>PAS TRÈS BIEN/MAL……………………..3<br>IL N'Y AVAIT AUCUN AUTRE AGENT……4	
**Q47.**	Avez-vous eu le sentiment que les informations qui vous ont été données pendant votre visite aujourd'hui étaient trop peu, beaucoup trop, juste ce qu'il faut?	TROP PEU. . . . . …..……..…1<br>CE QU'IL FAUT. . . . . ……….…...2<br>BEAUCOUP . . . . ….……...3<br>NE SAIT PAS………………………………..8	

**Q48.**	Étiez-vous très satisfaite, satisfaite, quelque peu satisfaite, ou pas du tout satisfaite de votre visite d'aujourd'hui dans la structure?	TRÈS SATISFAITE. . . . . .1<br>SATISFAITE. . . . .2<br>QUELQUE PEU SATISFAITE . . . .3<br>PAS DU TOUT……………..………………....4	
**Q49.**	Reviendrez-vous vers cette structure pour vos besoins sanitaires à l'avenir ?	OUI . . . .1<br>NON . . . .2<br>NE SAIT PAS. 8	
**Q50.**	Recommanderez-vous à d'autres personnes de votre famille/des amies/voisines cette structure en vue d'obtenir des services de sante?	OUI . . . .1<br>NON . . . .2<br>NE SAIT PAS. . . . .…………..8	

**Q51.**	**VÉRIFIEZ Q4 et Q26 :**<br><br>SI **Q4 = 01** POUR PF OU **Q26= 1** (A REÇU DES INFORMATIONS EN PF) ☐ ↓	SI **Q4 = DIFFERENT DE 01** ET **Q26 = 2** (N'A PAS REÇU D'INFO EN PF) ☐	→ **Q54**
**Q52.**	L'agent vous a t-il montré des boites à images, des affiches, dépliants ou des échantillons de méthodes pendant votre discussion?	OUI . . . . 1<br>NON 2	
**Q53.**	Pendant la visite, vous a-t-on donné des supports IEC (dépliants, prospectus, affiches, etc.) sur la PF à emporter avec vous ?	OUI . . . . 1<br>NON 2	
**Q54.**	À présent, je voudrais vous poser des questions sur le coût des services de PF que vous avez reçus aujourd'hui dans cette structure<br><br>Quelle est la somme totale que vous avez déboursée pour toutes ces prestations et services ?<br><br>N'omettez pas d'y inclure toute autre dépense faite pour les tests en laboratoire et pour la consultation.<br><br>*[**NA** = clientes d'intégration n'ayant pas reçu de services PF]*	N'A RIEN PAYÉ (GRATUIT)…………… …00000<br><br>NE SAIT PAS……………………………….99998<br><br>NON APPLICABLE…………………………99993<br><br>**MONTANT TOTAL (CFA)** ☐☐☐☐☐	→ **Q56**<br><br>→ **Q56**<br><br>→ **Q56**
**Q55.**	Pensez-vous que ce prix est cher ou pas cher?	CHER………………………………….1<br>PAS CHER……………………………2<br>NE PEUT PAS DIRE…………………8	
**Q56.**	Avez-vous une assurance ou une institution (franchises) qui paie pour tous ou une partie des services que vous avez reçus au niveau de cette structure de santé ?	OUI . . . . 1<br>NON . . . . .2<br>NE SAIT PAS…... . . ……………8	

INFORMATIONS RELATIVES A LA STRUCTURE DE SANTE<br>*À présent, je voudrais vous poser des questions sur le transport et l'accès aux services de santé.*			
**N°**	**QUESTIONS**	***CODAGE***	***SAUT***
**Q57.**	Combien de temps avez-vous mis pour venir ici aujourd'hui ?	TEMPS MIS EN MINUTES ☐☐☐<br><br>NE SAIT PAS……………………………… 998	
**Q58.**	Quel a été le <u>**principal moyen**</u> de transport que vous avez utilisé pour arriver ici?	A PIED……………………………….………01<br>CHARRETTE/CALÈCHE………………..02<br>BUS/MINIBUS/CAR RAPIDE……………03<br>TAXI………………………………………04<br>BICYCLETTE…………………….………05<br>MOTOCYCLETTE/SCOOTER…………06<br>VOITURE PRIVÉE……………….……...07<br>AUTRE _______________________ 96<br>(PRÉCISEZ)	

**Q59.**	Pour quelles raisons avez-vous choisi cette structure aujourd'hui?<br><br>**RÉPONSES MULTIPLES POSSIBLES. ENCERCLER TOUT CE QUI EST MENTIONNE.**	PAS LOIN DE CHEZ MOI………………………….A<br>CONVENABLE PAR RAPPORT A MON LIEU DE TRAVAIL…………………………………….…..……B<br>CONVENABLE PAR RAPPORT A MES HORAIRES………………………………. . . ..……...C<br>VOUS POUVEZ PASSER INAPERÇU/ANONYME...D<br>BONNE RÉPUTATION . . . . ..…………... . . ..……. E<br>LE PERSONNEL EST DISCRET/ CONFIDENTIALITÉ GARANTIE………………………………………….F<br>C'EST PLUS ABORDABLE/MOINS CHER. ……….G<br>A ETE REFEREE A CETTE STRUCTURE . ……….H<br>CETTE STRUCTURE EST LOIN DE MON DOMICILE……………………………………………..I<br>OFFRE DES SERVICES DE QUALITÉ..………..….J<br>OFFRE LES SERVICES DÉSIRÉS …….……….….K<br>LA STRUCTURE ACCEPTE LES ASSURANCES/L'IPM………………………………..L<br>LES PRESTATAIRES TRAITENT BIEN LES CLIENTS……………………………………..…..…....M<br>HABITUDE/TOUTE LA FAMILLE Y VA.…………….N<br>AUTRES (PRÉCISER)______________________X<br>NE SAIT PAS…….. . ………………… . . . . ……...Z	

**Q60.**	Cette structure de santé est-elle la plus proche de votre lieu de travail?	OUI. . . . .…………1<br>NON. . . . .…..…2<br>NE TRAVAILLE PAS………………………3<br>NE SAIT PAS………………………………..8	
**Q61.**	Cette structure de santé est-elle la plus proche de votre domicile ?	OUI . . . .…………1<br>NON . . . .………2<br>NE SAIT PAS . . . .…..…………8	→ **Q64**<br><br>→ **Q64**
**Q62.**	Quel est le type de structure le plus proche de votre domicile?	**TYPE DE STRUCTURE DE SANTÉ**<br>**SECTEUR PUBLIC**<br>HÔPITAL ……………………………...…..………..….11<br>CENTRE SANTE ………..…………………….…….12<br>POSTE DE SANTE………………….…..…….…….13<br>CASE DE SANTE……………………….……………14<br>AUTRE PUBLIC______________________________16<br>(PRÉCISER)<br>**SECTEUR PRIVE FORMEL**<br>CLINIQUE PRIVÉE………...…………………….……21<br>CABINET PRIVE …………………………………..…22<br>DISPENSAIRE CONFESSIONNEL……..………..……23<br>AUTRE MÉDICAL PRIVE ______________________26<br>(PRÉCISER)<br>**ONG**<br>CLINIQUE D'ONG………………….….…………...….31<br>AUTRE STRUCTURE D'ONG_________________36<br>(PRÉCISER)<br><br>**AUTRE**_______________________________________96	
**Q63.**	Quelle était **<u>la principale raison</u>** pour laquelle vous n'êtes pas allée dans la structure la plus proche de votre domicile?<br><br>**NOTER UNE SEULE RÉPONSE.**	HEURES D'OUVERTURE NON CONVENABLES...01<br>MAUVAISE RÉPUTATION . . . .… . …...02<br>N'AIME PAS LE PERSONNEL. . . . .… . ..….03<br>PAS DE MÉDICAMENTS. . . . . ……..…. …...04<br>PRÉFÈRE RESTER ANONYME . . . .… . …...05<br>ELLE EST PLUS CHÈRE. . . . .…… … ……06<br>RÉFÉRENCE. . . . . …………………. .. ……07<br>PRÉFÈRE UNE STRUCTURE DE SANTE QUI EST PLUS PROCHE DU LIEU DE TRAVAIL…………08<br>SERVICE NON OUVERT…………………..……..09<br>PAS D'AGENTS FÉMININS…………………….……10<br>SERVICE DE MAUVAISE QUALITÉ……………..…11<br>N'OFFRE PAS LES SERVICES REQUIS………….12<br>PRESTATAIRES SOUVENT ABSENTS……….…..13<br>LE SERVICE N'ACCEPTE PAS L'IPM……………..14<br>NOTRE FAMILLE N'Y VA PAS………………………15<br><br>AUTRE___________________________ 96<br>(PRÉCISEZ)<br>NE SAIT PAS…………………………………….…98	

Q64.	Utilisez-vous cette structure sanitaire (la plus proche de votre domicile) pour d'autres services de santé?	OUI……………………………………………………1<br>NON…………………………………………………..2	→ Q66
Q65.	Pour quels autres services de santé allez-vous à la structure de santé la plus proche?<br><br>**RÉPONSES MULTIPLES POSSIBLES.**<br>**NE PAS LIRE LES RÉPONSES, ENCERCLER TOUT CE QUI EST MENTIONNE.**	CONSULTATION PRÉNATALE………………..A<br>SERVICES ACCOUCHEMENT………………..B<br>CONSULTATION POST NATALE..……………C<br>SUIVI DE LA CROISSANCE DES ENFANTS…D<br>CONSULTATION IST. . . . ….. . ……..……. E<br>CONSULTATION VIH.. . . . . ….…..…………. F<br>SERVICES CURATIFS . . . . …..…..……. .G<br>SERVICES DE NUTRITION………………..……..H<br>VACCINATION ENFANTS……………….……..I<br>SOINS APRÈS-AVORTEMENT……….………J<br>CONSEIL DÉPISTAGE VOLONTAIRE………K<br>PLANIFICATION FAMILIALE………...…………L<br><br>AUTRES SERVICES ___________________X<br>(PRÉCISER)	

EXPOSITION AUX MEDIA<br>***Maintenant, je voudrais vous poser des questions sur votre exposition aux media***			
**N°**	**QUESTIONS**	**CODAGE**	**SAUT**
**Q66.**	Avez-vous reçu des informations relatives à la planification familiale au cours de ces **trois derniers mois ?**	OUI……………………………………………………1<br>NON…………………………………………………..2<br>NE SE SOUVIENT PAS …………………………8	→ **Q68**<br>→ **Q68**
**Q67.**	Quels sont vos principales sources d'information relatives à la planification familiale ?<br><br>DEMANDER SÉPARÉMENT DES PRÉCISIONS SUR:<br>A. Sources médiatiques<br>B. Sources médicales<br>C. Sources communautaires<br>D. Sources interpersonnelles<br><br>**INSISTER**: Autre source ? **(POUR CHAQUE CATÉGORIE)**<br><br>**RÉPONSES MULTIPLES POSSIBLES. ENCERCLER TOUT CE QUI EST MENTIONNE.**	**SOURCES MÉDIATIQUES**<br>RADIO……………………………………………..AA<br>TV……………………..…………………………..AB<br>JOURNAUX/LIVRES…..…………………………AC<br>MAGAZINES…………………..…………………AD<br>PANNEAUX D'AFFICHAGE…………………..…AE<br>PEINTURE MURALE……………………….…….AF<br>INTERNET ……………………….………………..AG<br><br>**SOURCES MÉDICALES**<br>MÉDECIN………………..……………….……….BA<br>SAGE-FEMME..………………….…..…..……..BB<br>INFIRMIER/AI………………………………...………BC<br>MATRONE / ASC ..……………..........................BD<br>GUÉRISSEUR / ACCOUCHEUSE TRAD…..…BE<br>PHARMACIEN ……………….…………..……...BF<br><br>**SOURCES COMMUNAUTAIRES**<br>RELAIS COMMUNAUTAIRE …………………..…CA<br>ORGANISATION COMMUNAUTAIRES……….CB<br>VOLONTAIRES / PAIRS ÉDUCATEURS ……..CC<br>ÉCOLE……………………………………………..CD<br>TRAVAILLEUR D'ONG....………………..….CE<br>SOURCES RELIGIEUSES………………………CF<br>GROUPEMENTS FÉMININS……………………CG<br><br>**SOURCES INTERPERSONNELLES**<br>PARENTS……………………..…..……………..DA<br>BEAUX-PARENTS………………………………DB<br>ÉPOUX/PARTENAIRE……………………..……….DC<br>FRÈRES ET SŒURS……………………..…………..DD<br>BELLES SŒURS/BEAUX-FRÈRES……………DE<br>AMIS / VOISINS…………………...……………..DF<br>AUTRES PARENTS/CONNAISSANCES……….DG<br><br>**AUTRE (PRÉCISER)**______________________XX<br>**AUCUN……………………...……………………..YY**<br>**NE SAIT PAS ………………………………………..ZZ**	

CARACTÉRISTIQUES PERSONNELLES DE LA CLIENTE<br>*A présent, je vais vous poser des questions vous concernant.*			
**N°**	**QUESTIONS**	**CODAGE**	**SAUT**
**Q68.**	Avez-vous déjà fréquenté l'école?	OUI…………………………………………………1<br>NON ………………………………………………2	 →**Q71**
**Q69.**	Quel est le plus haut niveau d'études que vous avez atteint : primaire, secondaire ou supérieur ?	CORANIQUE SEULEMENT…………………….1<br>PRIMAIRE…………………………………….2<br>SECONDAIRE 1 ………………………………3<br>SECONDAIRE 2 ………………………………4<br>SUPÉRIEUR…………………………………5	→**Q71**<br><br><br><br>→**Q71**
**Q70.**	Quelle est la dernière année/classe vous avez achevée ?	CLASSE /NIVEAU/ ANNÉE………………[__|__]	
**Q71.**	Quelle est votre religion?	CATHOLIQUE………………………………1<br>AUTRE RELIGION CHRÉTIENNE…………..2<br>MUSULMAN…………………………………3<br>TRADITIONNELLE…………………………4<br>AUCUNE ELIGION…………………………5<br>AUTRE………………………………………6	
**Q72.**	Quelle est votre groupe ethnique ?	WOLOF/LEBOU……………………………………01<br>POULAR……………………………………………02<br>SERER………………………………………………03<br>DIOLA………………………………………………04<br>MANDINGUE………………………………………05<br>SONINKE……………………………………………06<br>AUTRE________________________________96<br>(PRECISER)	
**Q73.**	Quel est votre situation matrimoniale actuelle?<br><br>(**INSISTEZ POUR CONNAITRE LE STATUT EXACT)**	ACTUELLEMENT MARIÉ(E)………… ………1<br>VIT AVEC UN HOMME………… ………………2<br><br>DIVORCÉE……………………………… ………3<br>SÉPARÉE……………………… ………………4<br>VEUVE…………………………………………5<br>CÉLIBATAIRE, JAMAIS MARIÉE…………………6	 <br><br>}→**Q76**
**Q74.**	Vivez-vous actuellement avec votre époux/partenaire où bien vit-il ailleurs?	VIVENT ENSEMBLE……………………………..1<br>ÉPOUX /PARTENAIRE VIT AILLEURS …………..2	
**Q75.**	Avez-vous déjà discuté de Planification Familiale avec votre partenaire ?	OUI ………………………………………………1<br>NON ………………………………………………2	
**Q76.**	Avez-vous discuté de la planification familiale avec quelqu'un [d'autre] au cours des **6 derniers mois** ?	OUI…………………………………………………1<br>NON…………………………………………………2<br>NE SAIT PAS……………………………………8	
**Q77.**	**VÉRIFIER Q4: POUR SERVICES D'ACCOUCHEMENT OU Q22 ACTUELLEMENT ENCEINTE**<br><br>SI **Q4**=PLANNING FAMILIAL **(01),** SUIVI DE LA CROISSANCE **(06),** VACCINATION DES ENFANTS **(07),** CONSULTATION IST **(08),** CONSULTATION VIH/SIDA **(09),** SERVICES CURATIFS **(10),** CDV **(11),** AUTRE **(96) ET Q22=2 OU 8** POUR **PAS** ACTUELLEMENT ENCEINTE ☐↓	SI **Q4= (02)** SOINS PRÉNATALS, **(03)** SERVICES ACCOUCHEMENT, **(04)** SOINS POSTNATALS, OU **(05)** SOINS APRÈS-AVORTEMENT, OU **Q22=1** POUR ACTUELLEMENT ENCEINTE ☐	→**Q79**
**Q78.**	Avez-vous déjà été enceinte?	OUI . . . . ……………………1<br>NON . . . . …………………… 2	 →**Q80**
**Q79.**	Combien d'enfants vivants avez-vous?<br><br>**NOTER LE NOMBRE DONNÉ**	NOMBRE D'ENFANTS ……………………………[__|__]<br>AUCUN …………………………………………..00<br>NE SAIT PAS ……………………………………..98	
**Q80.**	[Après la naissance de cet enfant] Voudriez-vous avoir un (autre) enfant dans l'avenir?	OUI . . . . ……………………1<br>NON . . . . ………………….2<br>DÉPEND DU CONJOINT . . . . ……………………3<br>DÉPEND DE DIEU . . . . ……………………4<br>NE PEUT PAS TOMBER ENCEINTE…………………5<br>NE SAIT PAS . . . . ………………………8	 <br><br>}→**Q82**

**Q81.**	[Après la naissance de cet enfant] Pendant combien de temps voudriez-vous attendre avant d'avoir un (autre) enfant ?	MOINS D'UN AN . . . . . . …. . …..............................1<br>UN A DEUX ANS . . . . ….. . . ……………………….2<br>PLUS DE DEUX ANS . . ……. ……………………….3<br>NE SAIT PAS . . . .……………………..8	
**Q82.**	Combien de fois avez-vous eu des rapports sexuels au cours des **trois (3) derniers mois**?	NOMBRE DE FOIS……………………… [__|__|__]<br><br>**OU**<br>AUCUNE………………………………………000<br>CHAQUE JOUR………………………………991<br>CHAQUE SEMAINE…………………………992<br>CHAQUE MOIS………………………………..993<br>AUTRE ____________________________996<br>(PRÉCISER)<br>NE SAIT PAS…………………………………..998	
**Q83.**	Quelqu'un vous a –t-il accompagné à votre visite d'aujourd'hui ?	OUI . . . .……………..1<br>NON . . . . ………….2	→ Q85
**Q84.**	Qui est-ce qui vous accompagné ?<br><br>**ENCERCLER TOUT CE QUI EST MENTIONNE**	ENFANT(S)...…………………………A<br>CONJOINT…………………………..B<br>MÈRE………………………………..C<br>BELLE-MÈRE……………………….D<br>AMI(E)……………………………....E<br>AUTRE __________________ X	

Je voudrais à présent vous poser des questions relatives à votre demeure/ lieu de résidence			
**Q85.**	Quel est votre lieu de résidence actuel ?	NOM VILLAGE/VILLE ______________________<br><br>NOM DÉPARTEMENT ____________________<br>[|]<br>A CODER AU BUREAU<br><br>RÉGION ______________________ [|]<br>A CODER AU BUREAU	
**Q86.**	Quel est le **principal** matériau dont est faite la toiture de votre demeure ?<br><br>**ENCERCLER UNE SEULE RÉPONSE**	**TOITURE NATURELLE**<br>AUCUN TOIT……………..……………..………………..11<br>CHAUME/FEUILLES DE PALMIER/<br>ROSEAU…………………………..……………..…12<br>HERBE/PAILLE…………….……………………...……13<br><br>**TOITURE RUDIMENTAIRE**<br>PAILLASSON RUSTIQUE ……………..……..………21<br>PALMIER/BAMBOO…………..………………….…...22<br>PLANCHES EN BOIS……………..………………..….23<br>CARTON…..……………………...…………………..……24<br><br>**TOITURE AMÉLIORÉE**<br>ARDOISE/ FIBRE DE CIMENT...…………...……......31<br>BOIS…………………………………………..…..……..32<br>TUILES …..…………………..……………….....………33<br>CIMENT………………..……..…………..…...………. 34<br>BARDEAU DE COUVERTURE...………..….…………35<br>ZINC/MÉTAL……………………………………..……..…36<br><br>**AUTRE**__________________________________96<br>(PRÉCISER)	

**Q87.**	De quel type de toilettes dispose votre ménage?	CHASSE<br>BRANCHÉE A ÉGOUT……………………….…...01<br>BRANCHÉE A FOSSE SEPTIQUE…………….…..02<br>BRANCHÉE A LATRINE…………………..………03<br>BRANCHÉE AILLEURS…………………………..04<br>BRANCHÉE A STRUCTURE INCONNUE………05<br>LATRINE VENTILÉE ET AMÉLIORÉE………………06<br>LATRINE DALLÉE (EN CIMENT, EN BOIS, ETC.)…07<br>TOILETTE COMPOSTÉE……………………………..08<br>LATRINE SANS DALLE / FOSSE OUVERTE………09<br>SEAU / BAQUET……………………………….………10<br>PAS DE TOILETTE/ LA NATURE…………………..…11<br>AUTRE_________________________________96_<br>(PRÉCISER)	11 → **Q90**
**Q88.**	Ces toilettes sont-elles situées à l'intérieur ou à l'extérieur de la maison ?	A L'INTERIEUR ....1<br>A L'EXTERIEUR……………………………..2	
**Q89.**	D'autres ménages partagent-ils ces toilettes avec vous ?	OUI .... 1<br>NON.. 2<br>NE SAIT PAS……………………………….8	
**Q90.**	Quelle est la source d'eau de boisson dans votre ménage?	**EAU ROBINET**<br>DANS LOGEMENT……....……...............................11<br>DANS COUR/CONCESSION................................12<br>ROBINET DU VOISIN........……………………...….13<br>ROBINET PUBLIC /BORNE FONTAINE.....……..14<br>**EAU DE PUITS NON COUVERT**<br>PUITS OUVERT DANS LOGEMENT……………......21<br>PUITS OUVERT DANS COURS/CONCESSION….22<br>PUITS PUBLIC OUVERT……………….………….23<br>**EAU DE PUITS PROTÈGE**<br>PUITS COUVERT PROTÈGE DANS LOGEMENT…………………………………………..31<br>PUITS COUVERT DANS COUR/CONCESSION……………………..……..32<br>PUITS PUBLIC PROTÈGE……......……….……..33<br>**EAU DE SOURCE**<br>SOURCE PROTÉGÉE..41<br>SOURCE NON-PROTÉGÉE (MARE, RIVIÈRE, ETC.)…………………………………………………...42<br>**EAU DE PLUIE...51**<br>**CAMION-CITERNE A EAU.......................................61**<br>**EAU EN BOUTEILLE…...…………………………........71**<br>**AUTRE**___________________________________96<br>(PRÉCISER)	
**Q91.**	Quel type de combustible utilise-t-on principalement dans votre ménage pour faire la cuisine ?	ÉLECTRICITÉ……….…………………………………..01<br>GAZ BOUTEILLE…..……………...............................02<br>CHARBON DE BOIS…………………...……………..03<br>BOIS À BRÛLER, PAILLE……….……….…………..04<br>BOUSE……..…………………………………………..05<br>NE CUISINE PAS…………………………….………06<br>AUTRE_________________________________96<br>(PRÉCISER)	
**Q92.**	Combien de pièces y a-t-il dans votre ménage, notamment les chambres à coucher et toutes les autres pièces (toilettes et cuisine exclues)?	PIECES (TOTAL)……………………… |__|__|	
**Q93.**	Votre ménage dispose-t-il d'électricité?	OUI ……………………………………………1<br>NON…………………………………………2	
**Q94.**	Votre ménage dispose-t-il d'un groupe électrogène?	OUI ……………………………………………1<br>NON…………………………………………2	

**Q95.**	Votre ménage dispose-t-il d'une radio?	OUI ……………………………………………1<br>NON……………………………………………2	
**Q96.**	Votre ménage dispose-t-il d'une TV?	OUI ……………………………………………1<br>NON……………………………………………2	→ **Q100**
**Q97.**	Votre ménage dispose-t-il d'un vidéo/DVD player?	OUI ……………………………………………1<br>NON……………………………………………2	
**Q98.**	Votre ménage dispose-t-il d'une antenne MMDS/TV5?	OUI ……………………………………………1<br>NON……………………………………………2	
**Q99.**	Votre ménage dispose-t-il de Canal Sat / Horizons?	OUI ……………………………………………1<br>NON……………………………………………2	
**Q100.**	Votre ménage dispose-t-il d'un téléphone fixe?	OUI ……………………………………………1<br>NON……………………………………………2	
**Q101.**	Votre ménage dispose-t-il d'un téléphone mobile?	OUI ……………………………………………1<br>NON……………………………………………2	
**Q102.**	Votre ménage dispose-t-il d'un réfrigérateur/congélateur?	OUI ……………………………………………1<br>NON……………………………………………2	
**Q103.**	Votre ménage dispose-t-il d'un ordinateur ?	OUI ……………………………………………1<br>NON……………………………………………2	
**Q104.**	Votre ménage dispose-t-il d'un climatiseur?	OUI ……………………………………………1<br>NON……………………………………………2	
**Q105.**	Votre ménage dispose-t-il d'une voiture?	OUI ……………………………………………1<br>NON……………………………………………2	
	MENTIONNER L'HEURE EXACTE DE LA FIN DE L'ENTRETIEN	__ __ : __ __	

Merci beaucoup pour avoir pris le temps de répondre à mes questions. Une fois de plus, les informations que vous m'avez données seront gardées strictement confidentielles. Bonne journée!

OBSERVATIONS DE L'ENQUÊTRICE

OBSERVATIONS DU SUPERVISEUR
